# Supplementary material for: Super-resolution microscopy reveals majorly mono- and dimeric presenilin1/γ-secretase at the cell surface
Source: eLife. 2020 Jul 7;9:e56679. doi: 10.7554/eLife.56679 (PMC7340497; doi:10.7554/eLife.56679)
Supplement: Figure 1—source data 1. [file elife-56679-fig1-data1.zip › Figure1 - Source Data 1/Figure1-Source Data1.docx]

**Source Data for Nearest Neighbor Analysis (Figure 1)**

This .zip folder contains all SIM data used for quantification of Nearest Neighbor distances related to Figure 1-I. Each folder relates to different cell line: “GFP-PSEN1 GFPnb” folder relates to sKO GFP-PSEN1 cell line stained with GFPnanobody-ATTO647n; “GFP-PSEN1 NCT-SNAP” folder relates to TKO NCT-SNAP GFP-PSEN1 cell line stained with SiR-substrate.

Each folder contains subfolders showing ROIs with background substraction (done by adjusting intensity histogram) and ROIs mask (done by H-watershed, ImageJ). The final mask was used to localize spot centorids used during nearest naighbor analysis.
