# Supplementary figures and images for: Super-resolution microscopy reveals majorly mono- and dimeric presenilin1/γ-secretase at the cell surface (part 1 of 4)
Source: eLife. 2020 Jul 7;9:e56679. doi: 10.7554/eLife.56679 (PMC7340497; doi:10.7554/eLife.56679)

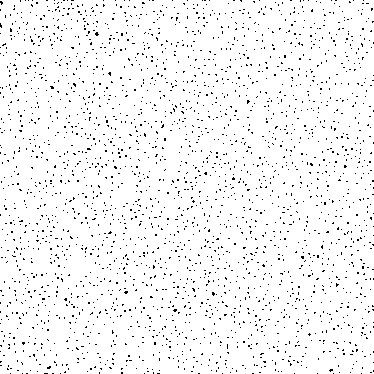

Supplement: Figure 1—source data 1. [file elife-56679-fig1-data1.zip › Figure1 - Source Data 1/GFP-PSEN1 GFP-nb/roi masks/12-PS-1.tif - watershed (h=1404,00, T=4213,00, %=20, n=2625).tif]

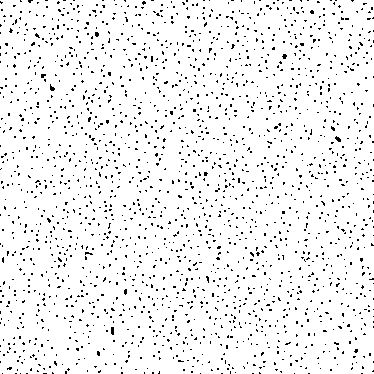

Supplement: Figure 1—source data 1. [file elife-56679-fig1-data1.zip › Figure1 - Source Data 1/GFP-PSEN1 GFP-nb/roi masks/13-Nb-1.tif - watershed (h=1404,00, T=4213,00, %=20, n=2041).tif]

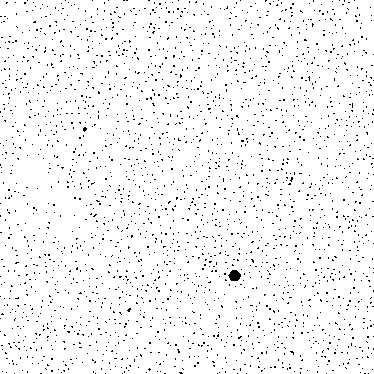

Supplement: Figure 1—source data 1. [file elife-56679-fig1-data1.zip › Figure1 - Source Data 1/GFP-PSEN1 GFP-nb/roi masks/17-PS-1.tif - watershed (h=1404,00, T=4213,00, %=20, n=2451).tif]

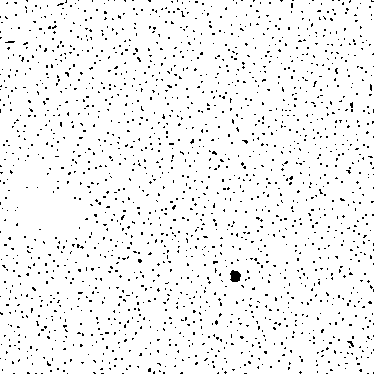

Supplement: Figure 1—source data 1. [file elife-56679-fig1-data1.zip › Figure1 - Source Data 1/GFP-PSEN1 GFP-nb/roi masks/18-Nb-1.tif - watershed (h=1404,00, T=4213,00, %=20, n=1821).tif]

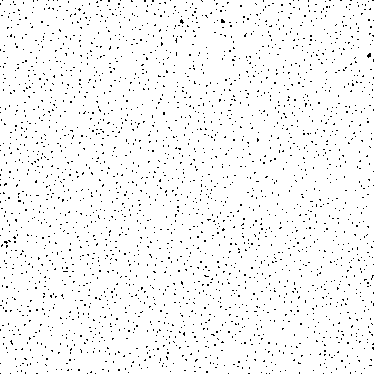

Supplement: Figure 1—source data 1. [file elife-56679-fig1-data1.zip › Figure1 - Source Data 1/GFP-PSEN1 GFP-nb/roi masks/19-PS-1.tif - watershed (h=1404,00, T=4213,00, %=20, n=2528).tif]

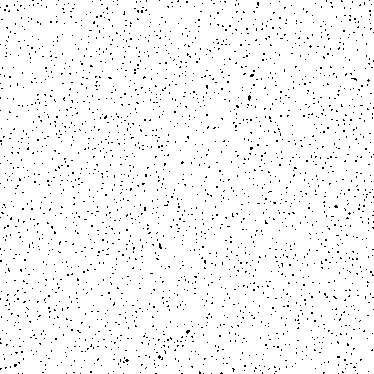

Supplement: Figure 1—source data 1. [file elife-56679-fig1-data1.zip › Figure1 - Source Data 1/GFP-PSEN1 GFP-nb/roi masks/1-PS-1.tif - watershed (h=1404,00, T=4213,00, %=20, n=2373).tif]

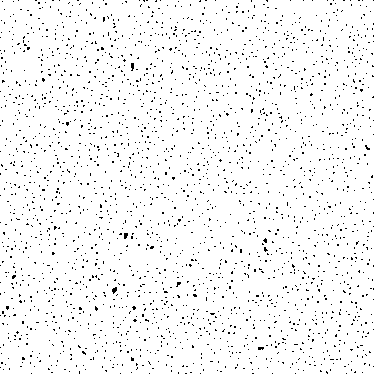

Supplement: Figure 1—source data 1. [file elife-56679-fig1-data1.zip › Figure1 - Source Data 1/GFP-PSEN1 GFP-nb/roi masks/1-PS-2.tif - watershed (h=1404,00, T=4213,00, %=20, n=2422).tif]

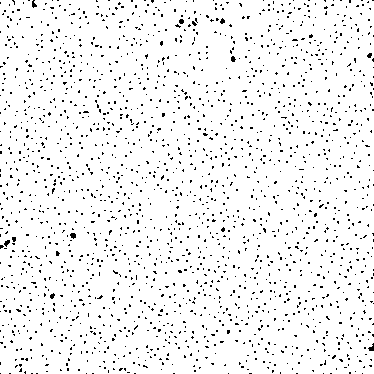

Supplement: Figure 1—source data 1. [file elife-56679-fig1-data1.zip › Figure1 - Source Data 1/GFP-PSEN1 GFP-nb/roi masks/20-Nb-1.tif - watershed (h=1404,00, T=4213,00, %=20, n=1953).tif]

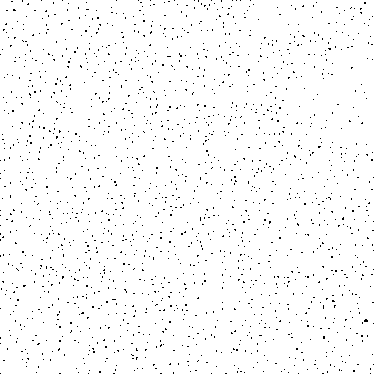

Supplement: Figure 1—source data 1. [file elife-56679-fig1-data1.zip › Figure1 - Source Data 1/GFP-PSEN1 GFP-nb/roi masks/21-ps-1.tif - watershed (h=1404,00, T=4213,00, %=20, n=1704).tif]

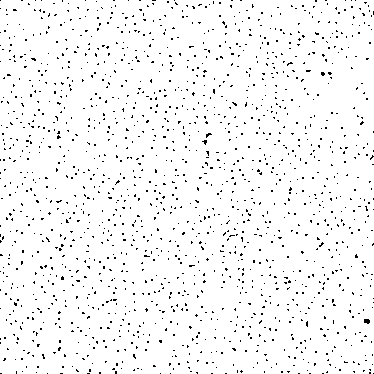

Supplement: Figure 1—source data 1. [file elife-56679-fig1-data1.zip › Figure1 - Source Data 1/GFP-PSEN1 GFP-nb/roi masks/22-nb-1.tif - watershed (h=1404,00, T=4213,00, %=20, n=1627).tif]

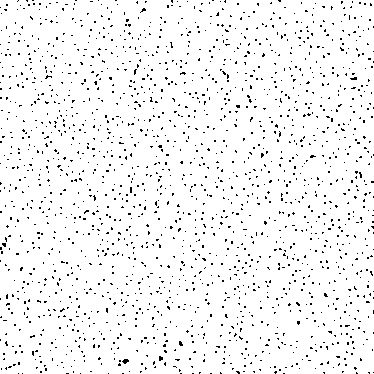

Supplement: Figure 1—source data 1. [file elife-56679-fig1-data1.zip › Figure1 - Source Data 1/GFP-PSEN1 GFP-nb/roi masks/2-Nb-1.tif - watershed (h=1404,00, T=4213,00, %=20, n=1878).tif]

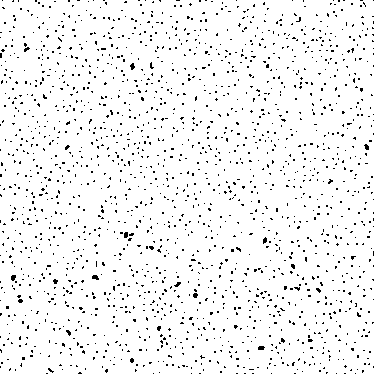

Supplement: Figure 1—source data 1. [file elife-56679-fig1-data1.zip › Figure1 - Source Data 1/GFP-PSEN1 GFP-nb/roi masks/2-Nb-2.tif - watershed (h=1404,00, T=4213,00, %=20, n=1855).tif]

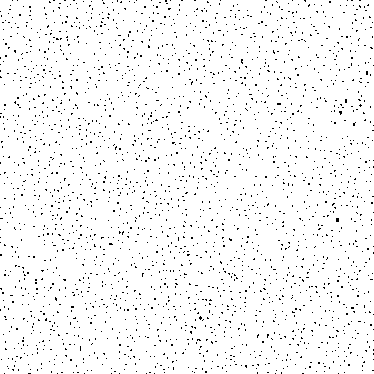

Supplement: Figure 1—source data 1. [file elife-56679-fig1-data1.zip › Figure1 - Source Data 1/GFP-PSEN1 GFP-nb/roi masks/3-PS-1.tif - watershed (h=1404,00, T=4213,00, %=20, n=2463).tif]

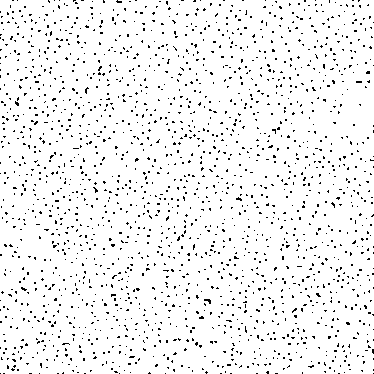

Supplement: Figure 1—source data 1. [file elife-56679-fig1-data1.zip › Figure1 - Source Data 1/GFP-PSEN1 GFP-nb/roi masks/4-Nb-1.tif - watershed (h=1404,00, T=4213,00, %=20, n=1974).tif]

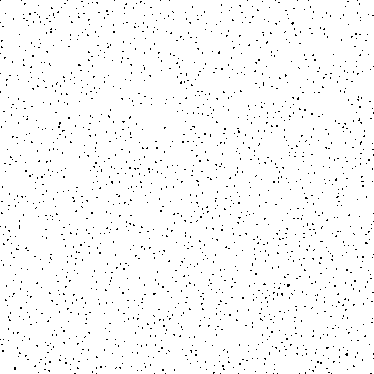

Supplement: Figure 1—source data 1. [file elife-56679-fig1-data1.zip › Figure1 - Source Data 1/GFP-PSEN1 GFP-nb/roi masks/8-PS-1.tif - watershed (h=1404,00, T=4213,00, %=20, n=1762).tif]

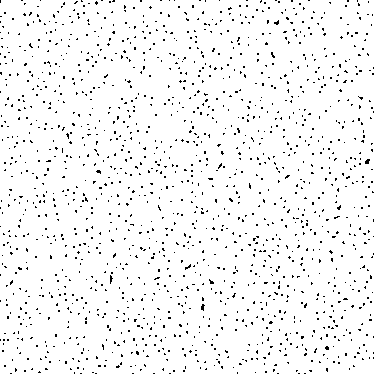

Supplement: Figure 1—source data 1. [file elife-56679-fig1-data1.zip › Figure1 - Source Data 1/GFP-PSEN1 GFP-nb/roi masks/9-NB-1.tif - watershed (h=1404,00, T=4213,00, %=20, n=1571).tif]

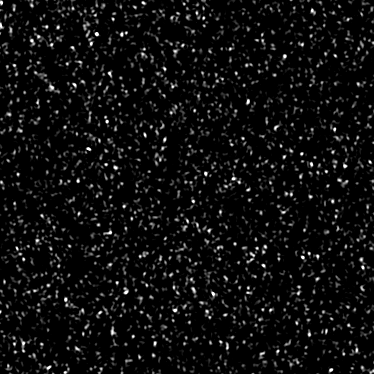

Supplement: Figure 1—source data 1. [file elife-56679-fig1-data1.zip › Figure1 - Source Data 1/GFP-PSEN1 GFP-nb/rois/12-PS-1.tif]

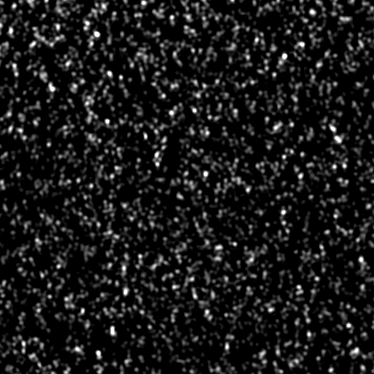

Supplement: Figure 1—source data 1. [file elife-56679-fig1-data1.zip › Figure1 - Source Data 1/GFP-PSEN1 GFP-nb/rois/13-Nb-1.tif]

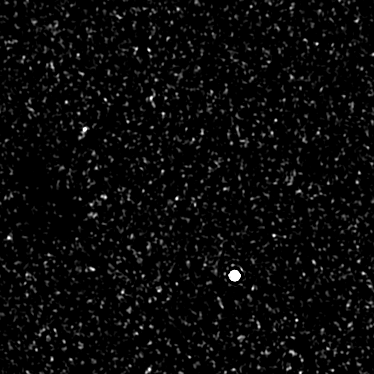

Supplement: Figure 1—source data 1. [file elife-56679-fig1-data1.zip › Figure1 - Source Data 1/GFP-PSEN1 GFP-nb/rois/17-PS-1.tif]

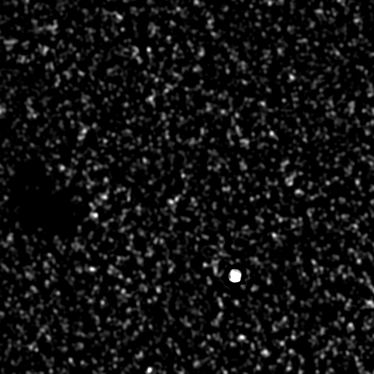

Supplement: Figure 1—source data 1. [file elife-56679-fig1-data1.zip › Figure1 - Source Data 1/GFP-PSEN1 GFP-nb/rois/18-Nb-1.tif]

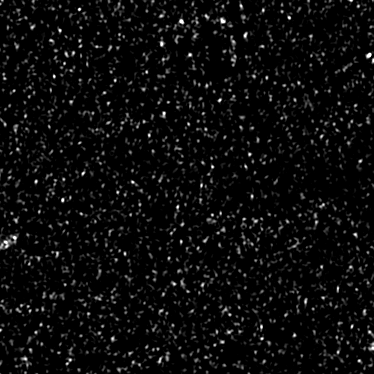

Supplement: Figure 1—source data 1. [file elife-56679-fig1-data1.zip › Figure1 - Source Data 1/GFP-PSEN1 GFP-nb/rois/19-PS-1.tif]

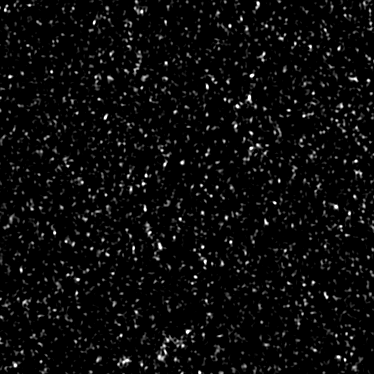

Supplement: Figure 1—source data 1. [file elife-56679-fig1-data1.zip › Figure1 - Source Data 1/GFP-PSEN1 GFP-nb/rois/1-PS-1.tif]

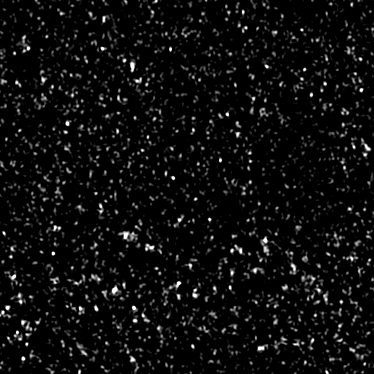

Supplement: Figure 1—source data 1. [file elife-56679-fig1-data1.zip › Figure1 - Source Data 1/GFP-PSEN1 GFP-nb/rois/1-PS-2.tif]

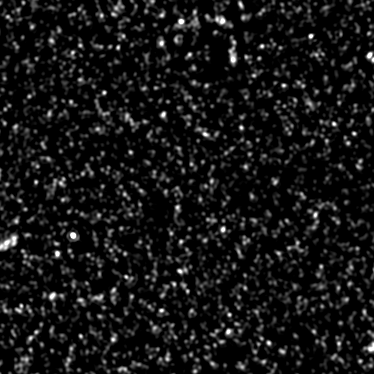

Supplement: Figure 1—source data 1. [file elife-56679-fig1-data1.zip › Figure1 - Source Data 1/GFP-PSEN1 GFP-nb/rois/20-Nb-1.tif]

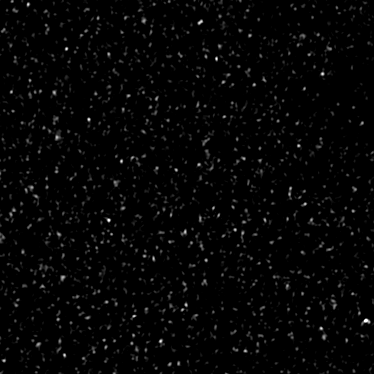

Supplement: Figure 1—source data 1. [file elife-56679-fig1-data1.zip › Figure1 - Source Data 1/GFP-PSEN1 GFP-nb/rois/21-ps-1.tif]

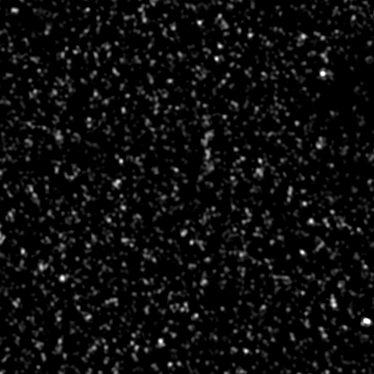

Supplement: Figure 1—source data 1. [file elife-56679-fig1-data1.zip › Figure1 - Source Data 1/GFP-PSEN1 GFP-nb/rois/22-nb-1.tif]

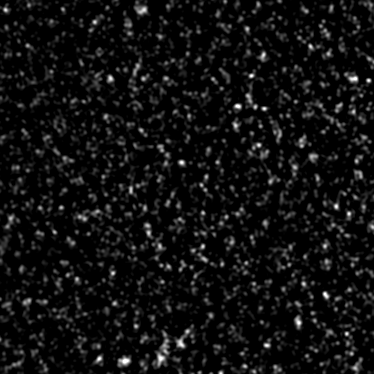

Supplement: Figure 1—source data 1. [file elife-56679-fig1-data1.zip › Figure1 - Source Data 1/GFP-PSEN1 GFP-nb/rois/2-Nb-1.tif]

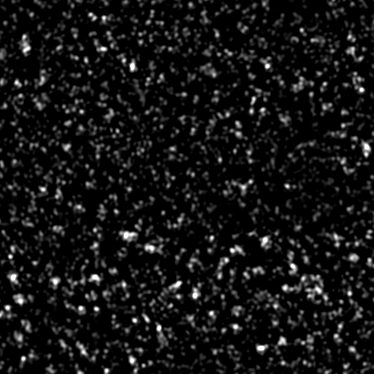

Supplement: Figure 1—source data 1. [file elife-56679-fig1-data1.zip › Figure1 - Source Data 1/GFP-PSEN1 GFP-nb/rois/2-Nb-2.tif]

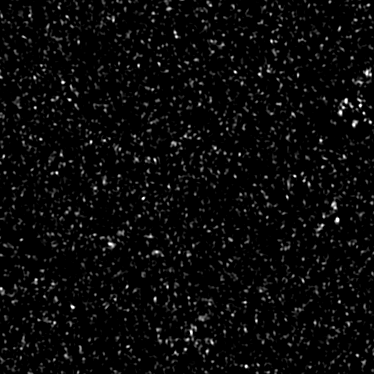

Supplement: Figure 1—source data 1. [file elife-56679-fig1-data1.zip › Figure1 - Source Data 1/GFP-PSEN1 GFP-nb/rois/3-PS-1.tif]

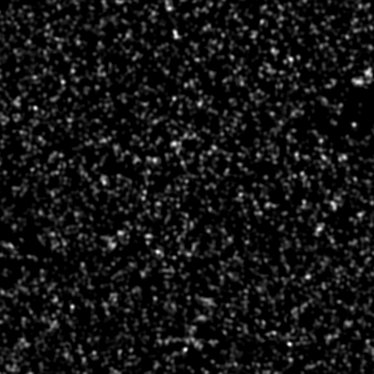

Supplement: Figure 1—source data 1. [file elife-56679-fig1-data1.zip › Figure1 - Source Data 1/GFP-PSEN1 GFP-nb/rois/4-Nb-1.tif]

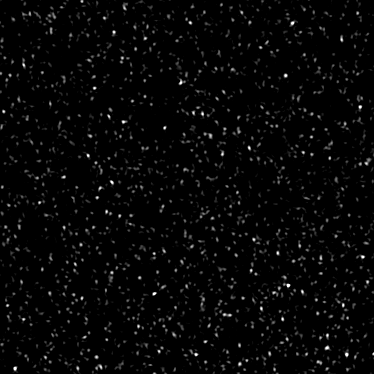

Supplement: Figure 1—source data 1. [file elife-56679-fig1-data1.zip › Figure1 - Source Data 1/GFP-PSEN1 GFP-nb/rois/8-PS-1.tif]

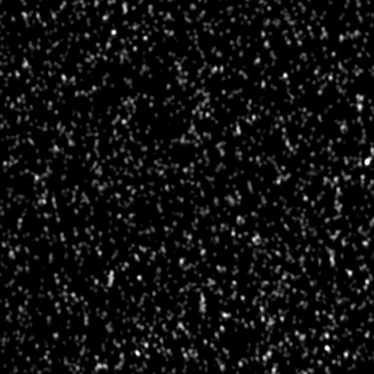

Supplement: Figure 1—source data 1. [file elife-56679-fig1-data1.zip › Figure1 - Source Data 1/GFP-PSEN1 GFP-nb/rois/9-NB-1.tif]

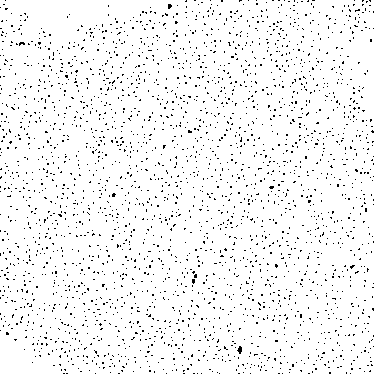

Supplement: Figure 1—source data 1. [file elife-56679-fig1-data1.zip › Figure1 - Source Data 1/GFP-PSEN1 NCT-SNAP/roi mask/1_PS-1.tif - watershed (h=1404,00, T=4213,00, %=20, n=2727).tif]

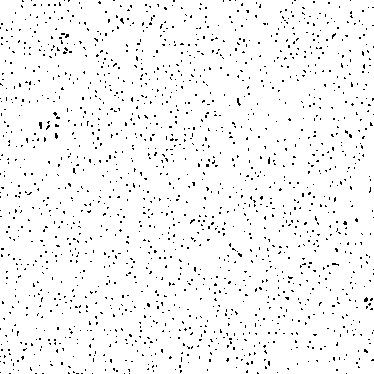

Supplement: Figure 1—source data 1. [file elife-56679-fig1-data1.zip › Figure1 - Source Data 1/GFP-PSEN1 NCT-SNAP/roi mask/10_NCT-1.tif - watershed (h=1404,00, T=4213,00, %=20, n=1660).tif]

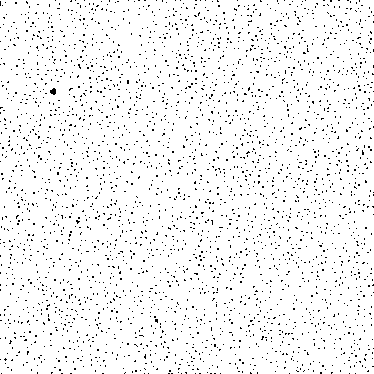

Supplement: Figure 1—source data 1. [file elife-56679-fig1-data1.zip › Figure1 - Source Data 1/GFP-PSEN1 NCT-SNAP/roi mask/11_PS-1.tif - watershed (h=1404,00, T=4213,00, %=20, n=2988).tif]

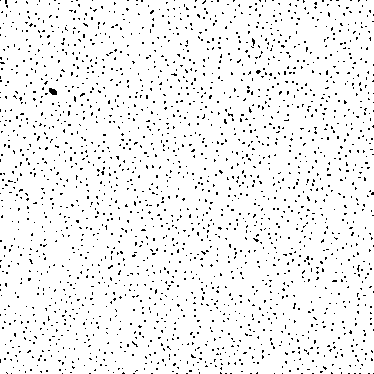

Supplement: Figure 1—source data 1. [file elife-56679-fig1-data1.zip › Figure1 - Source Data 1/GFP-PSEN1 NCT-SNAP/roi mask/12_NCt-1.tif - watershed (h=1404,00, T=4213,00, %=20, n=2174).tif]

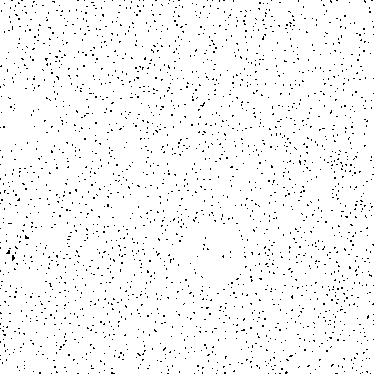

Supplement: Figure 1—source data 1. [file elife-56679-fig1-data1.zip › Figure1 - Source Data 1/GFP-PSEN1 NCT-SNAP/roi mask/13_PS-1.tif - watershed (h=1404,00, T=4213,00, %=20, n=2247).tif]

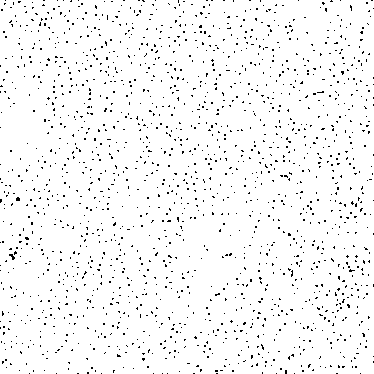

Supplement: Figure 1—source data 1. [file elife-56679-fig1-data1.zip › Figure1 - Source Data 1/GFP-PSEN1 NCT-SNAP/roi mask/14_NCT-1.tif - watershed (h=1404,00, T=4213,00, %=20, n=1742).tif]

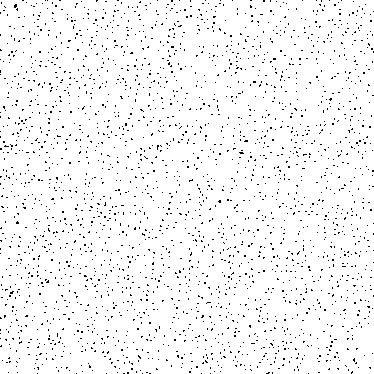

Supplement: Figure 1—source data 1. [file elife-56679-fig1-data1.zip › Figure1 - Source Data 1/GFP-PSEN1 NCT-SNAP/roi mask/15_PS-1.tif - watershed (h=1404,00, T=4213,00, %=20, n=2540).tif]

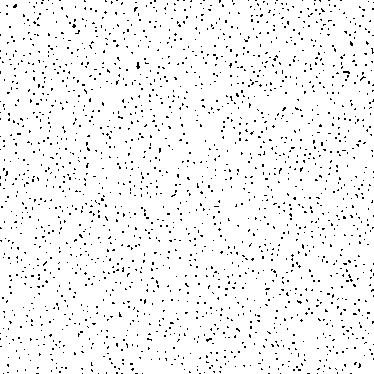

Supplement: Figure 1—source data 1. [file elife-56679-fig1-data1.zip › Figure1 - Source Data 1/GFP-PSEN1 NCT-SNAP/roi mask/16_NCT-1.tif - watershed (h=1404,00, T=4213,00, %=20, n=1991).tif]

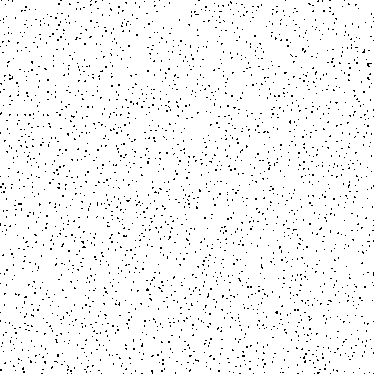

Supplement: Figure 1—source data 1. [file elife-56679-fig1-data1.zip › Figure1 - Source Data 1/GFP-PSEN1 NCT-SNAP/roi mask/17_PS-1.tif - watershed (h=1404,00, T=4213,00, %=20, n=2436).tif]

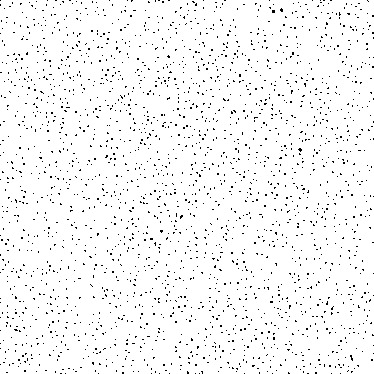

Supplement: Figure 1—source data 1. [file elife-56679-fig1-data1.zip › Figure1 - Source Data 1/GFP-PSEN1 NCT-SNAP/roi mask/17_PS-2.tif - watershed (h=1404,00, T=4213,00, %=20, n=2318).tif]

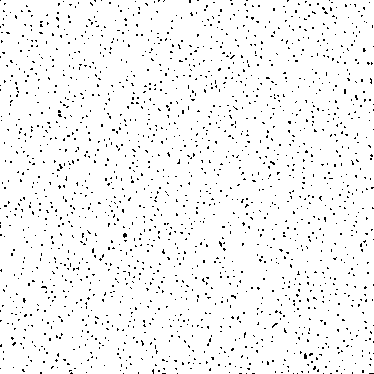

Supplement: Figure 1—source data 1. [file elife-56679-fig1-data1.zip › Figure1 - Source Data 1/GFP-PSEN1 NCT-SNAP/roi mask/18_NCT-1.tif - watershed (h=1404,00, T=4213,00, %=20, n=1842).tif]

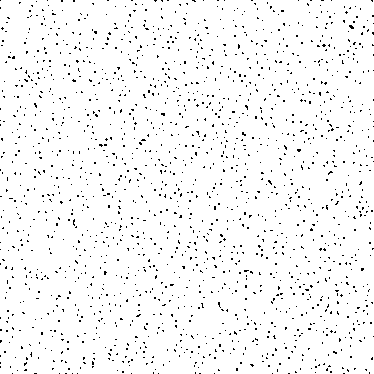

Supplement: Figure 1—source data 1. [file elife-56679-fig1-data1.zip › Figure1 - Source Data 1/GFP-PSEN1 NCT-SNAP/roi mask/18_NCT-2.tif - watershed (h=1404,00, T=4213,00, %=20, n=1709).tif]

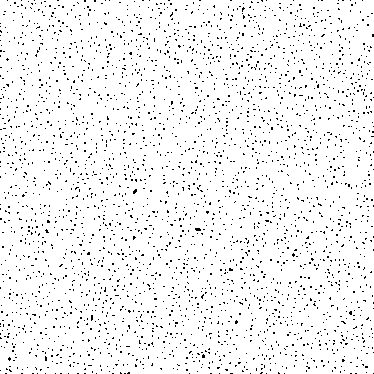

Supplement: Figure 1—source data 1. [file elife-56679-fig1-data1.zip › Figure1 - Source Data 1/GFP-PSEN1 NCT-SNAP/roi mask/19_PS-1.tif - watershed (h=1404,00, T=4213,00, %=20, n=2967).tif]

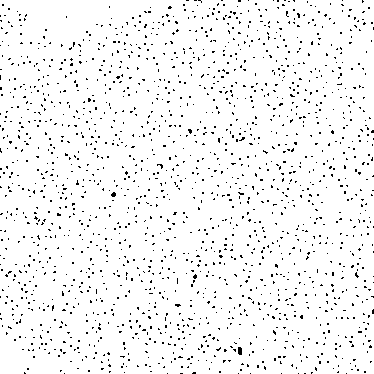

Supplement: Figure 1—source data 1. [file elife-56679-fig1-data1.zip › Figure1 - Source Data 1/GFP-PSEN1 NCT-SNAP/roi mask/2_NCt-1.tif - watershed (h=1404,00, T=4213,00, %=20, n=1793).tif]

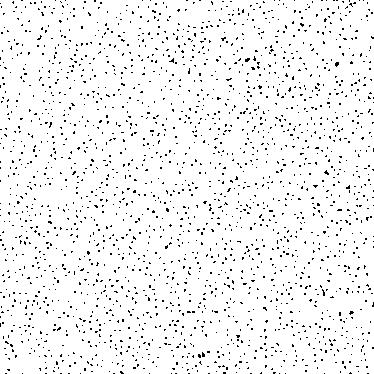

Supplement: Figure 1—source data 1. [file elife-56679-fig1-data1.zip › Figure1 - Source Data 1/GFP-PSEN1 NCT-SNAP/roi mask/20_NCT-1.tif - watershed (h=1404,00, T=4213,00, %=20, n=2157).tif]

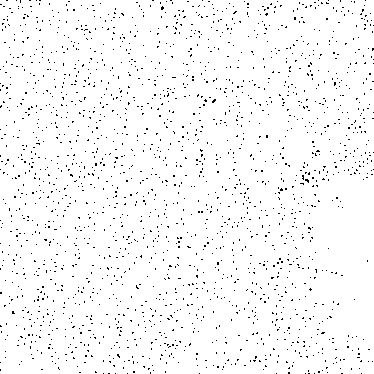

Supplement: Figure 1—source data 1. [file elife-56679-fig1-data1.zip › Figure1 - Source Data 1/GFP-PSEN1 NCT-SNAP/roi mask/3_PS-1.tif - watershed (h=1404,00, T=4213,00, %=20, n=2063).tif]

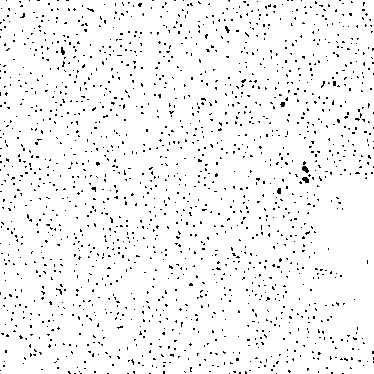

Supplement: Figure 1—source data 1. [file elife-56679-fig1-data1.zip › Figure1 - Source Data 1/GFP-PSEN1 NCT-SNAP/roi mask/4_NCT-1.tif - watershed (h=1404,00, T=4213,00, %=20, n=1704).tif]

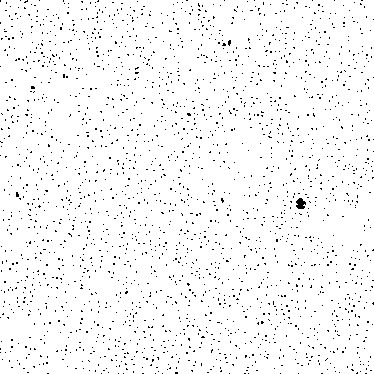

Supplement: Figure 1—source data 1. [file elife-56679-fig1-data1.zip › Figure1 - Source Data 1/GFP-PSEN1 NCT-SNAP/roi mask/5_PS-1.tif - watershed (h=1404,00, T=4213,00, %=20, n=2240).tif]

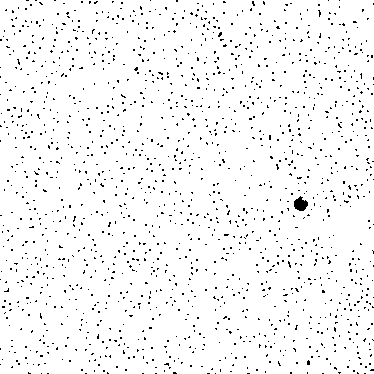

Supplement: Figure 1—source data 1. [file elife-56679-fig1-data1.zip › Figure1 - Source Data 1/GFP-PSEN1 NCT-SNAP/roi mask/6_NCT-1.tif - watershed (h=1404,00, T=4213,00, %=20, n=1560).tif]

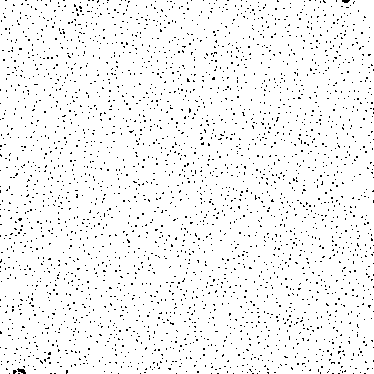

Supplement: Figure 1—source data 1. [file elife-56679-fig1-data1.zip › Figure1 - Source Data 1/GFP-PSEN1 NCT-SNAP/roi mask/7_PS-1.tif - watershed (h=1404,00, T=4213,00, %=20, n=2886).tif]

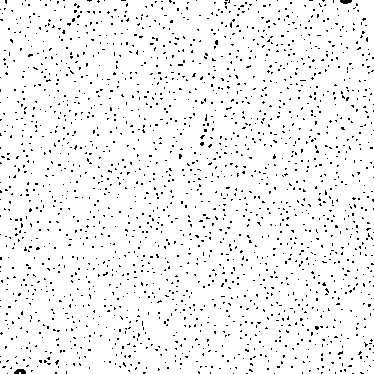

Supplement: Figure 1—source data 1. [file elife-56679-fig1-data1.zip › Figure1 - Source Data 1/GFP-PSEN1 NCT-SNAP/roi mask/8_NCT-1.tif - watershed (h=1404,00, T=4213,00, %=20, n=2041).tif]

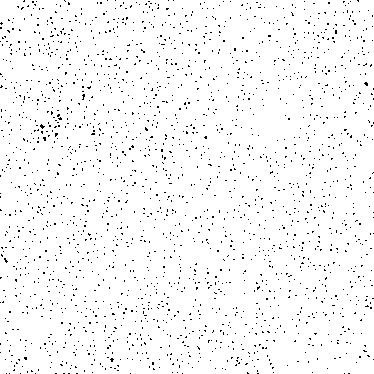

Supplement: Figure 1—source data 1. [file elife-56679-fig1-data1.zip › Figure1 - Source Data 1/GFP-PSEN1 NCT-SNAP/roi mask/9_PS-1.tif - watershed (h=1404,00, T=4213,00, %=20, n=2118).tif]

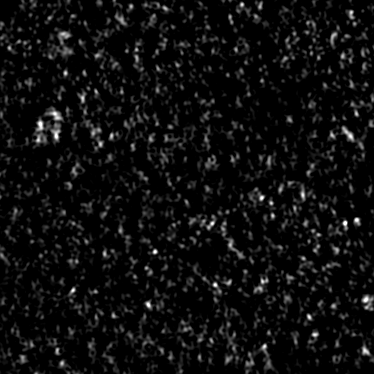

Supplement: Figure 1—source data 1. [file elife-56679-fig1-data1.zip › Figure1 - Source Data 1/GFP-PSEN1 NCT-SNAP/rois/nct/10_NCT-1.tif]

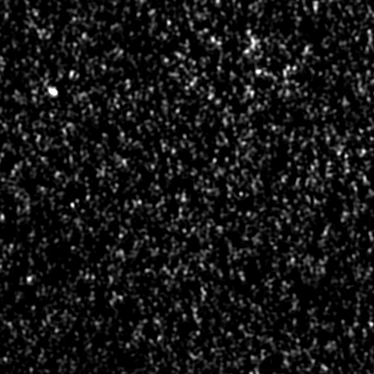

Supplement: Figure 1—source data 1. [file elife-56679-fig1-data1.zip › Figure1 - Source Data 1/GFP-PSEN1 NCT-SNAP/rois/nct/12_NCt-1.tif]

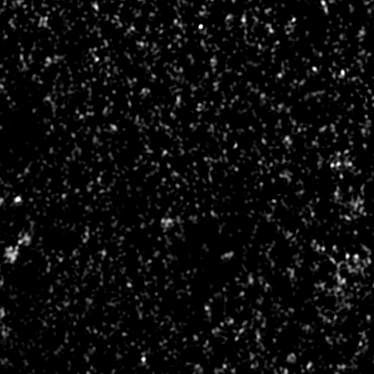

Supplement: Figure 1—source data 1. [file elife-56679-fig1-data1.zip › Figure1 - Source Data 1/GFP-PSEN1 NCT-SNAP/rois/nct/14_NCT-1.tif]

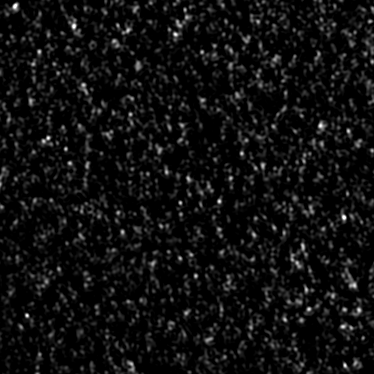

Supplement: Figure 1—source data 1. [file elife-56679-fig1-data1.zip › Figure1 - Source Data 1/GFP-PSEN1 NCT-SNAP/rois/nct/16_NCT-1.tif]

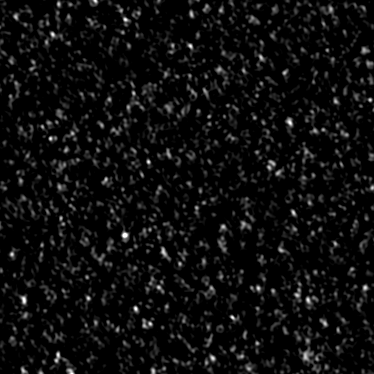

Supplement: Figure 1—source data 1. [file elife-56679-fig1-data1.zip › Figure1 - Source Data 1/GFP-PSEN1 NCT-SNAP/rois/nct/18_NCT-1.tif]

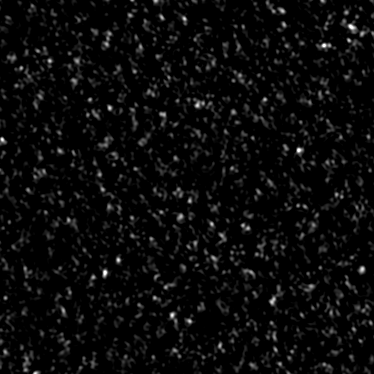

Supplement: Figure 1—source data 1. [file elife-56679-fig1-data1.zip › Figure1 - Source Data 1/GFP-PSEN1 NCT-SNAP/rois/nct/18_NCT-2.tif]

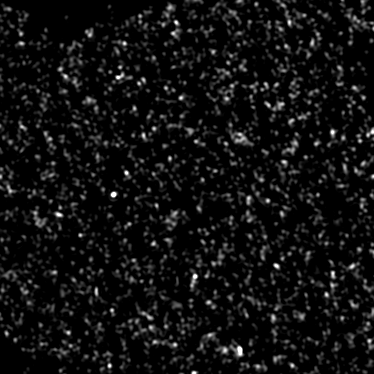

Supplement: Figure 1—source data 1. [file elife-56679-fig1-data1.zip › Figure1 - Source Data 1/GFP-PSEN1 NCT-SNAP/rois/nct/2_NCt-1.tif]

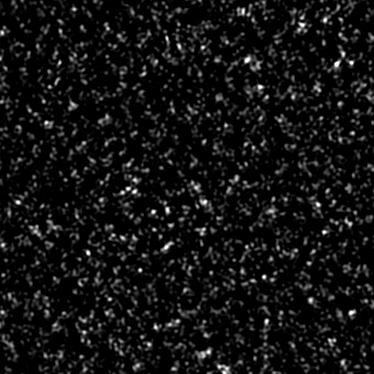

Supplement: Figure 1—source data 1. [file elife-56679-fig1-data1.zip › Figure1 - Source Data 1/GFP-PSEN1 NCT-SNAP/rois/nct/20_NCT-1.tif]

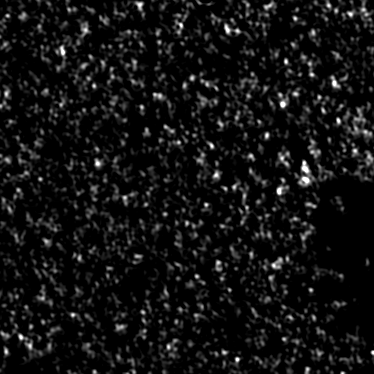

Supplement: Figure 1—source data 1. [file elife-56679-fig1-data1.zip › Figure1 - Source Data 1/GFP-PSEN1 NCT-SNAP/rois/nct/4_NCT-1.tif]

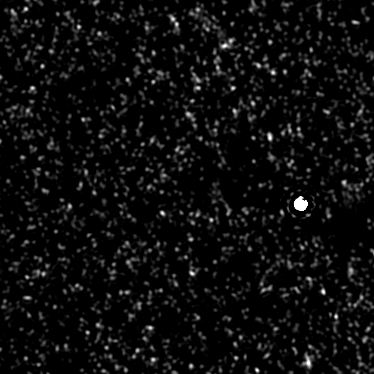

Supplement: Figure 1—source data 1. [file elife-56679-fig1-data1.zip › Figure1 - Source Data 1/GFP-PSEN1 NCT-SNAP/rois/nct/6_NCT-1.tif]

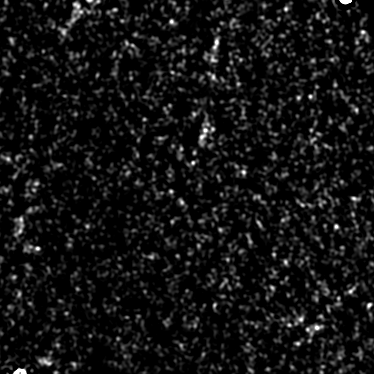

Supplement: Figure 1—source data 1. [file elife-56679-fig1-data1.zip › Figure1 - Source Data 1/GFP-PSEN1 NCT-SNAP/rois/nct/8_NCT-1.tif]

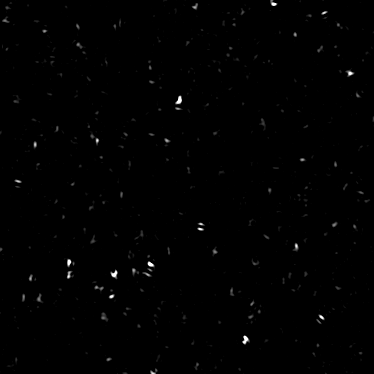

Supplement: Figure 1—source data 1. [file elife-56679-fig1-data1.zip › Figure1 - Source Data 1/GFP-PSEN1 NCT-SNAP/rois/ps/1.tif]

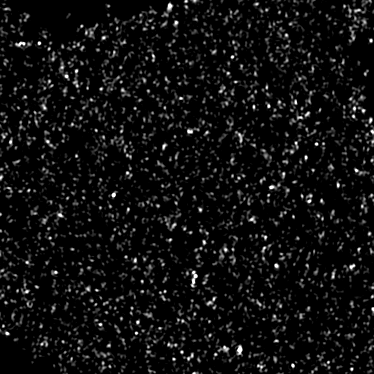

Supplement: Figure 1—source data 1. [file elife-56679-fig1-data1.zip › Figure1 - Source Data 1/GFP-PSEN1 NCT-SNAP/rois/ps/1_PS-1.tif]

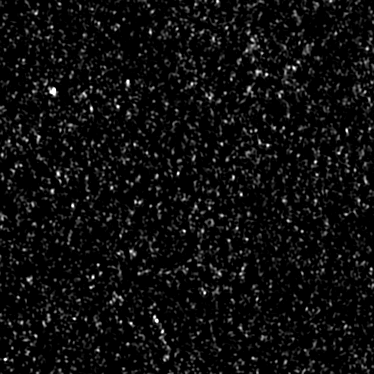

Supplement: Figure 1—source data 1. [file elife-56679-fig1-data1.zip › Figure1 - Source Data 1/GFP-PSEN1 NCT-SNAP/rois/ps/11_PS-1.tif]

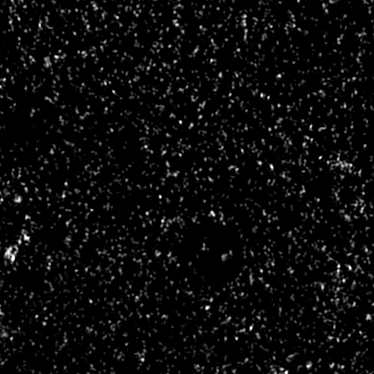

Supplement: Figure 1—source data 1. [file elife-56679-fig1-data1.zip › Figure1 - Source Data 1/GFP-PSEN1 NCT-SNAP/rois/ps/13_PS-1.tif]

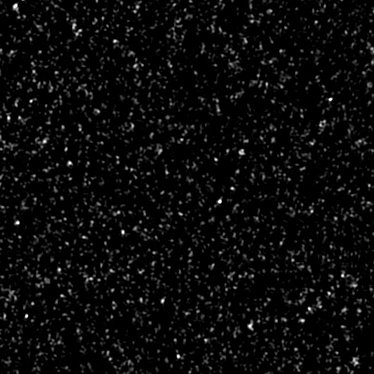

Supplement: Figure 1—source data 1. [file elife-56679-fig1-data1.zip › Figure1 - Source Data 1/GFP-PSEN1 NCT-SNAP/rois/ps/15_PS-1.tif]

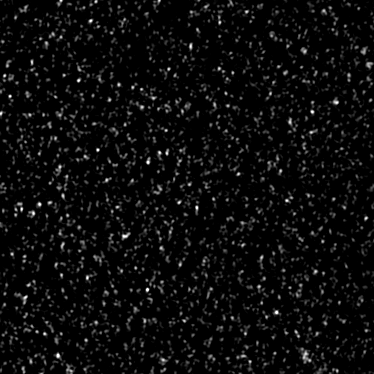

Supplement: Figure 1—source data 1. [file elife-56679-fig1-data1.zip › Figure1 - Source Data 1/GFP-PSEN1 NCT-SNAP/rois/ps/17_PS-1.tif]

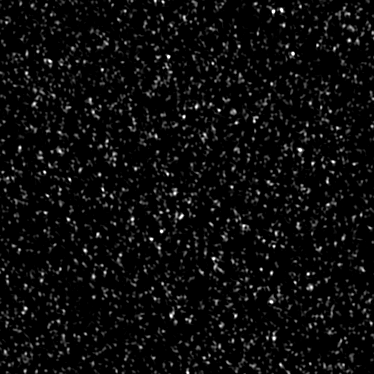

Supplement: Figure 1—source data 1. [file elife-56679-fig1-data1.zip › Figure1 - Source Data 1/GFP-PSEN1 NCT-SNAP/rois/ps/17_PS-2.tif]

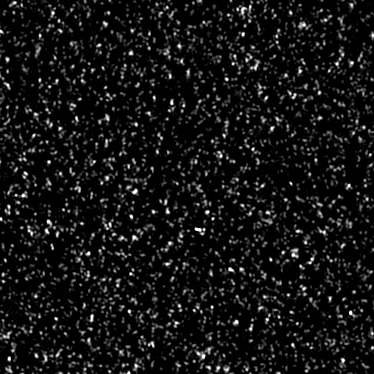

Supplement: Figure 1—source data 1. [file elife-56679-fig1-data1.zip › Figure1 - Source Data 1/GFP-PSEN1 NCT-SNAP/rois/ps/19_PS-1.tif]

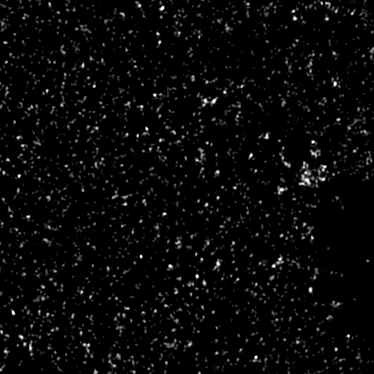

Supplement: Figure 1—source data 1. [file elife-56679-fig1-data1.zip › Figure1 - Source Data 1/GFP-PSEN1 NCT-SNAP/rois/ps/3_PS-1.tif]

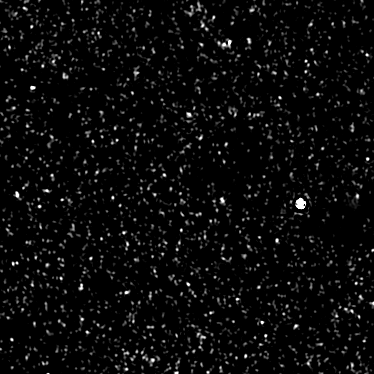

Supplement: Figure 1—source data 1. [file elife-56679-fig1-data1.zip › Figure1 - Source Data 1/GFP-PSEN1 NCT-SNAP/rois/ps/5_PS-1.tif]

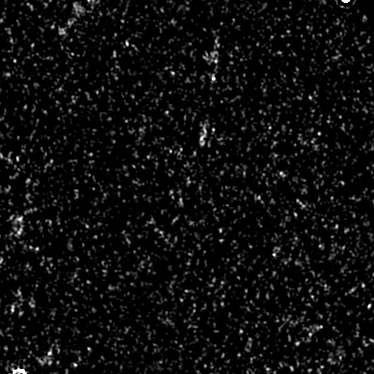

Supplement: Figure 1—source data 1. [file elife-56679-fig1-data1.zip › Figure1 - Source Data 1/GFP-PSEN1 NCT-SNAP/rois/ps/7_PS-1.tif]

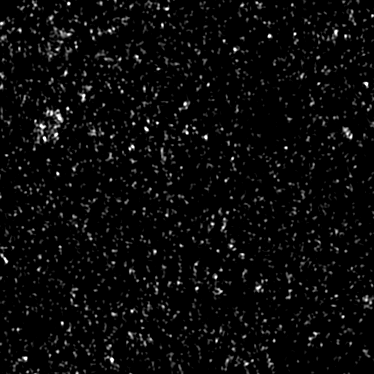

Supplement: Figure 1—source data 1. [file elife-56679-fig1-data1.zip › Figure1 - Source Data 1/GFP-PSEN1 NCT-SNAP/rois/ps/9_PS-1.tif]

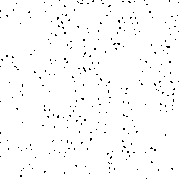

Supplement: Figure 2—source data 1. [file elife-56679-fig2-data1.zip › Figure2 - Source Data 1/NCT-GFP NCT-SNAP/roi mask/005_NCTSNAP-1.tif - watershed (h= 0,00, T= 0,00, %=20, n=282).tif]

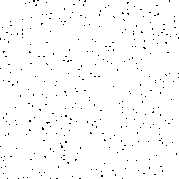

Supplement: Figure 2—source data 1. [file elife-56679-fig2-data1.zip › Figure2 - Source Data 1/NCT-GFP NCT-SNAP/roi mask/006_NCTGFP-1.tif - watershed (h=1404,00, T=4213,00, %=20, n=329).tif]

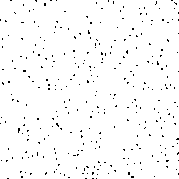

Supplement: Figure 2—source data 1. [file elife-56679-fig2-data1.zip › Figure2 - Source Data 1/NCT-GFP NCT-SNAP/roi mask/009_NCTSNAP-1.tif - watershed (h= 0,00, T= 0,00, %=20, n=311).tif]

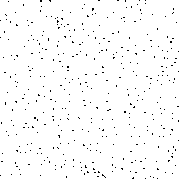

Supplement: Figure 2—source data 1. [file elife-56679-fig2-data1.zip › Figure2 - Source Data 1/NCT-GFP NCT-SNAP/roi mask/010_NCTGFP-1.tif - watershed (h=1404,00, T=4213,00, %=20, n=390).tif]

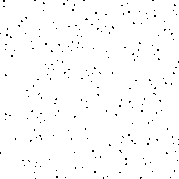

Supplement: Figure 2—source data 1. [file elife-56679-fig2-data1.zip › Figure2 - Source Data 1/NCT-GFP NCT-SNAP/roi mask/012_NCTSNAP-1.tif - watershed (h= 0,00, T= 0,00, %=20, n=220).tif]

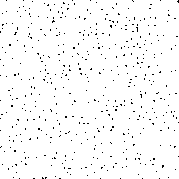

Supplement: Figure 2—source data 1. [file elife-56679-fig2-data1.zip › Figure2 - Source Data 1/NCT-GFP NCT-SNAP/roi mask/013_NCTGFP-1.tif - watershed (h=1404,00, T=4213,00, %=20, n=460).tif]

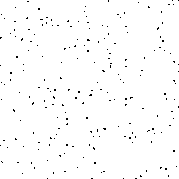

Supplement: Figure 2—source data 1. [file elife-56679-fig2-data1.zip › Figure2 - Source Data 1/NCT-GFP NCT-SNAP/roi mask/015_NCTSNAP-1.tif - watershed (h= 0,00, T= 0,00, %=20, n=202).tif]

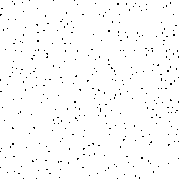

Supplement: Figure 2—source data 1. [file elife-56679-fig2-data1.zip › Figure2 - Source Data 1/NCT-GFP NCT-SNAP/roi mask/016_NCTGFP-1.tif - watershed (h=1404,00, T=4213,00, %=20, n=340).tif]

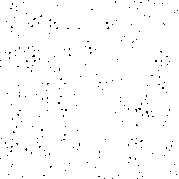

Supplement: Figure 2—source data 1. [file elife-56679-fig2-data1.zip › Figure2 - Source Data 1/NCT-GFP NCT-SNAP/roi mask/018_NCTSNAP-1.tif - watershed (h= 0,00, T= 0,00, %=20, n=228).tif]

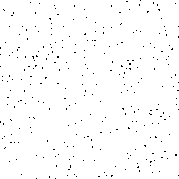

Supplement: Figure 2—source data 1. [file elife-56679-fig2-data1.zip › Figure2 - Source Data 1/NCT-GFP NCT-SNAP/roi mask/019_NCTGFP-1.tif - watershed (h=1404,00, T=4213,00, %=20, n=283).tif]

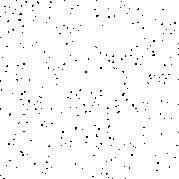

Supplement: Figure 2—source data 1. [file elife-56679-fig2-data1.zip › Figure2 - Source Data 1/NCT-GFP NCT-SNAP/roi mask/021_NCTSNAP-1.tif - watershed (h= 0,00, T= 0,00, %=20, n=288).tif]

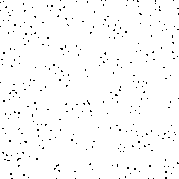

Supplement: Figure 2—source data 1. [file elife-56679-fig2-data1.zip › Figure2 - Source Data 1/NCT-GFP NCT-SNAP/roi mask/021_NCTSNAP-2.tif - watershed (h= 0,00, T= 0,00, %=20, n=308).tif]

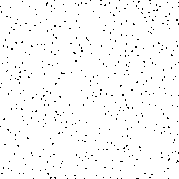

Supplement: Figure 2—source data 1. [file elife-56679-fig2-data1.zip › Figure2 - Source Data 1/NCT-GFP NCT-SNAP/roi mask/022_NCTGFP-1.tif - watershed (h=1404,00, T=4213,00, %=20, n=445).tif]

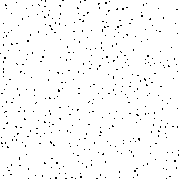

Supplement: Figure 2—source data 1. [file elife-56679-fig2-data1.zip › Figure2 - Source Data 1/NCT-GFP NCT-SNAP/roi mask/022_NCTGFP-2.tif - watershed (h=1404,00, T=4213,00, %=20, n=419).tif]

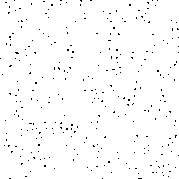

Supplement: Figure 2—source data 1. [file elife-56679-fig2-data1.zip › Figure2 - Source Data 1/NCT-GFP NCT-SNAP/roi mask/025_NCTSNAP-1.tif - watershed (h= 0,00, T= 0,00, %=20, n=294).tif]

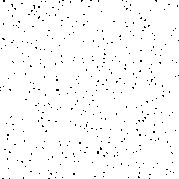

Supplement: Figure 2—source data 1. [file elife-56679-fig2-data1.zip › Figure2 - Source Data 1/NCT-GFP NCT-SNAP/roi mask/026_NCTGFP-1.tif - watershed (h=1404,00, T=4213,00, %=20, n=380).tif]

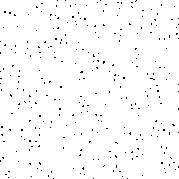

Supplement: Figure 2—source data 1. [file elife-56679-fig2-data1.zip › Figure2 - Source Data 1/NCT-GFP NCT-SNAP/roi mask/029_NCTSNAP-1.tif - watershed (h= 0,00, T= 0,00, %=20, n=294).tif]

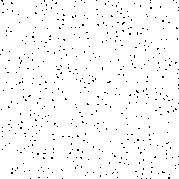

Supplement: Figure 2—source data 1. [file elife-56679-fig2-data1.zip › Figure2 - Source Data 1/NCT-GFP NCT-SNAP/roi mask/030_NCTGFP-1.tif - watershed (h=1404,00, T=4213,00, %=20, n=490).tif]

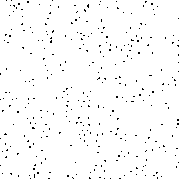

Supplement: Figure 2—source data 1. [file elife-56679-fig2-data1.zip › Figure2 - Source Data 1/NCT-GFP NCT-SNAP/roi mask/035_NCTSNAP-1.tif - watershed (h= 0,00, T= 0,00, %=20, n=324).tif]

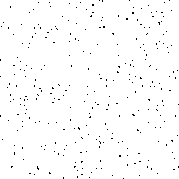

Supplement: Figure 2—source data 1. [file elife-56679-fig2-data1.zip › Figure2 - Source Data 1/NCT-GFP NCT-SNAP/roi mask/036_NCTGFP-1.tif - watershed (h=1404,00, T=4213,00, %=20, n=283).tif]

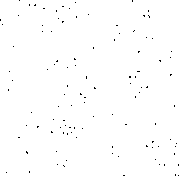

Supplement: Figure 2—source data 1. [file elife-56679-fig2-data1.zip › Figure2 - Source Data 1/NCT-GFP NCT-SNAP/roi mask/040_NCTSNAP-1.tif - watershed (h= 0,00, T= 0,00, %=20, n=107).tif]

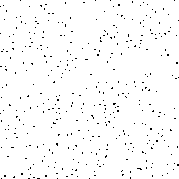

Supplement: Figure 2—source data 1. [file elife-56679-fig2-data1.zip › Figure2 - Source Data 1/NCT-GFP NCT-SNAP/roi mask/041_NCTGFP-1.tif - watershed (h=1404,00, T=4213,00, %=20, n=373).tif]

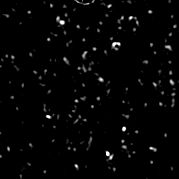

Supplement: Figure 2—source data 1. [file elife-56679-fig2-data1.zip › Figure2 - Source Data 1/NCT-GFP NCT-SNAP/rois/005_NCTSNAP-1.tif]
