# Supplementary figures and images for: Super-resolution microscopy reveals majorly mono- and dimeric presenilin1/γ-secretase at the cell surface (part 2 of 4)
Source: eLife. 2020 Jul 7;9:e56679. doi: 10.7554/eLife.56679 (PMC7340497; doi:10.7554/eLife.56679)

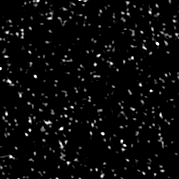

Supplement: Figure 2—source data 1. [file elife-56679-fig2-data1.zip › Figure2 - Source Data 1/NCT-GFP NCT-SNAP/rois/006_NCTGFP-1.tif]

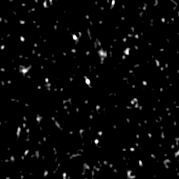

Supplement: Figure 2—source data 1. [file elife-56679-fig2-data1.zip › Figure2 - Source Data 1/NCT-GFP NCT-SNAP/rois/009_NCTSNAP-1.tif]

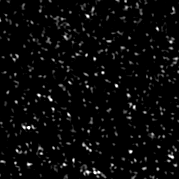

Supplement: Figure 2—source data 1. [file elife-56679-fig2-data1.zip › Figure2 - Source Data 1/NCT-GFP NCT-SNAP/rois/010_NCTGFP-1.tif]

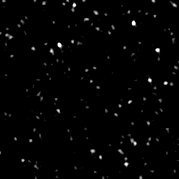

Supplement: Figure 2—source data 1. [file elife-56679-fig2-data1.zip › Figure2 - Source Data 1/NCT-GFP NCT-SNAP/rois/012_NCTSNAP-1.tif]

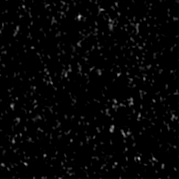

Supplement: Figure 2—source data 1. [file elife-56679-fig2-data1.zip › Figure2 - Source Data 1/NCT-GFP NCT-SNAP/rois/013_NCTGFP-1.tif]

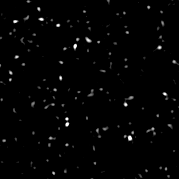

Supplement: Figure 2—source data 1. [file elife-56679-fig2-data1.zip › Figure2 - Source Data 1/NCT-GFP NCT-SNAP/rois/015_NCTSNAP-1.tif]

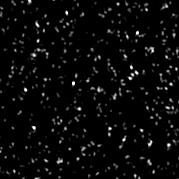

Supplement: Figure 2—source data 1. [file elife-56679-fig2-data1.zip › Figure2 - Source Data 1/NCT-GFP NCT-SNAP/rois/016_NCTGFP-1.tif]

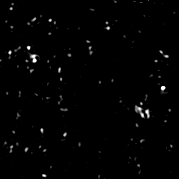

Supplement: Figure 2—source data 1. [file elife-56679-fig2-data1.zip › Figure2 - Source Data 1/NCT-GFP NCT-SNAP/rois/018_NCTSNAP-1.tif]

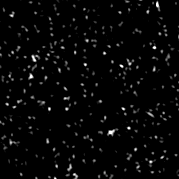

Supplement: Figure 2—source data 1. [file elife-56679-fig2-data1.zip › Figure2 - Source Data 1/NCT-GFP NCT-SNAP/rois/019_NCTGFP-1.tif]

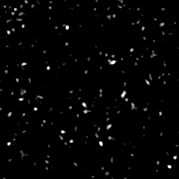

Supplement: Figure 2—source data 1. [file elife-56679-fig2-data1.zip › Figure2 - Source Data 1/NCT-GFP NCT-SNAP/rois/021_NCTSNAP-1.tif]

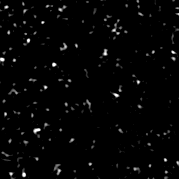

Supplement: Figure 2—source data 1. [file elife-56679-fig2-data1.zip › Figure2 - Source Data 1/NCT-GFP NCT-SNAP/rois/021_NCTSNAP-2.tif]

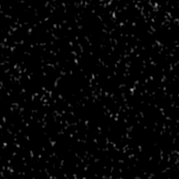

Supplement: Figure 2—source data 1. [file elife-56679-fig2-data1.zip › Figure2 - Source Data 1/NCT-GFP NCT-SNAP/rois/022_NCTGFP-1.tif]

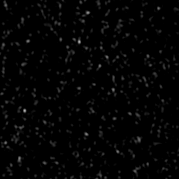

Supplement: Figure 2—source data 1. [file elife-56679-fig2-data1.zip › Figure2 - Source Data 1/NCT-GFP NCT-SNAP/rois/022_NCTGFP-2.tif]

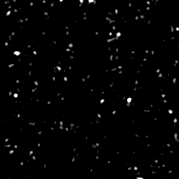

Supplement: Figure 2—source data 1. [file elife-56679-fig2-data1.zip › Figure2 - Source Data 1/NCT-GFP NCT-SNAP/rois/025_NCTSNAP-1.tif]

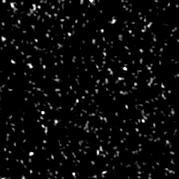

Supplement: Figure 2—source data 1. [file elife-56679-fig2-data1.zip › Figure2 - Source Data 1/NCT-GFP NCT-SNAP/rois/026_NCTGFP-1.tif]

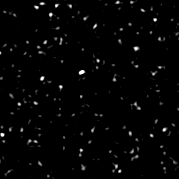

Supplement: Figure 2—source data 1. [file elife-56679-fig2-data1.zip › Figure2 - Source Data 1/NCT-GFP NCT-SNAP/rois/029_NCTSNAP-1.tif]

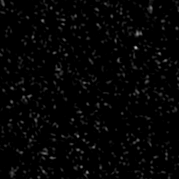

Supplement: Figure 2—source data 1. [file elife-56679-fig2-data1.zip › Figure2 - Source Data 1/NCT-GFP NCT-SNAP/rois/030_NCTGFP-1.tif]

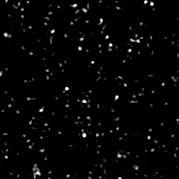

Supplement: Figure 2—source data 1. [file elife-56679-fig2-data1.zip › Figure2 - Source Data 1/NCT-GFP NCT-SNAP/rois/035_NCTSNAP-1.tif]

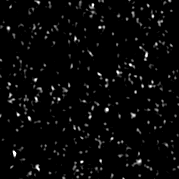

Supplement: Figure 2—source data 1. [file elife-56679-fig2-data1.zip › Figure2 - Source Data 1/NCT-GFP NCT-SNAP/rois/036_NCTGFP-1.tif]

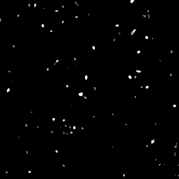

Supplement: Figure 2—source data 1. [file elife-56679-fig2-data1.zip › Figure2 - Source Data 1/NCT-GFP NCT-SNAP/rois/040_NCTSNAP-1.tif]

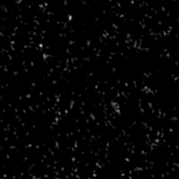

Supplement: Figure 2—source data 1. [file elife-56679-fig2-data1.zip › Figure2 - Source Data 1/NCT-GFP NCT-SNAP/rois/041_NCTGFP-1.tif]

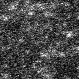

Supplement: Figure 2—source data 2. [file elife-56679-fig2-data2.zip › Figure2 - Source Data 2/ROIs/a_LP1.0-0.5-0.5_sub_bgsubs-1.tif]

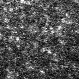

Supplement: Figure 2—source data 2. [file elife-56679-fig2-data2.zip › Figure2 - Source Data 2/ROIs/b_LP1.0-0.5-0.5_sub_bgsub-1.tif]

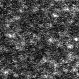

Supplement: Figure 2—source data 2. [file elife-56679-fig2-data2.zip › Figure2 - Source Data 2/ROIs/c_LP1.0-0.5-0.5_sub_bgsubs-1.tif]

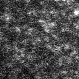

Supplement: Figure 2—source data 2. [file elife-56679-fig2-data2.zip › Figure2 - Source Data 2/ROIs/d_LP1.0-0.5-0.5_sub_bgsub-1.tif]

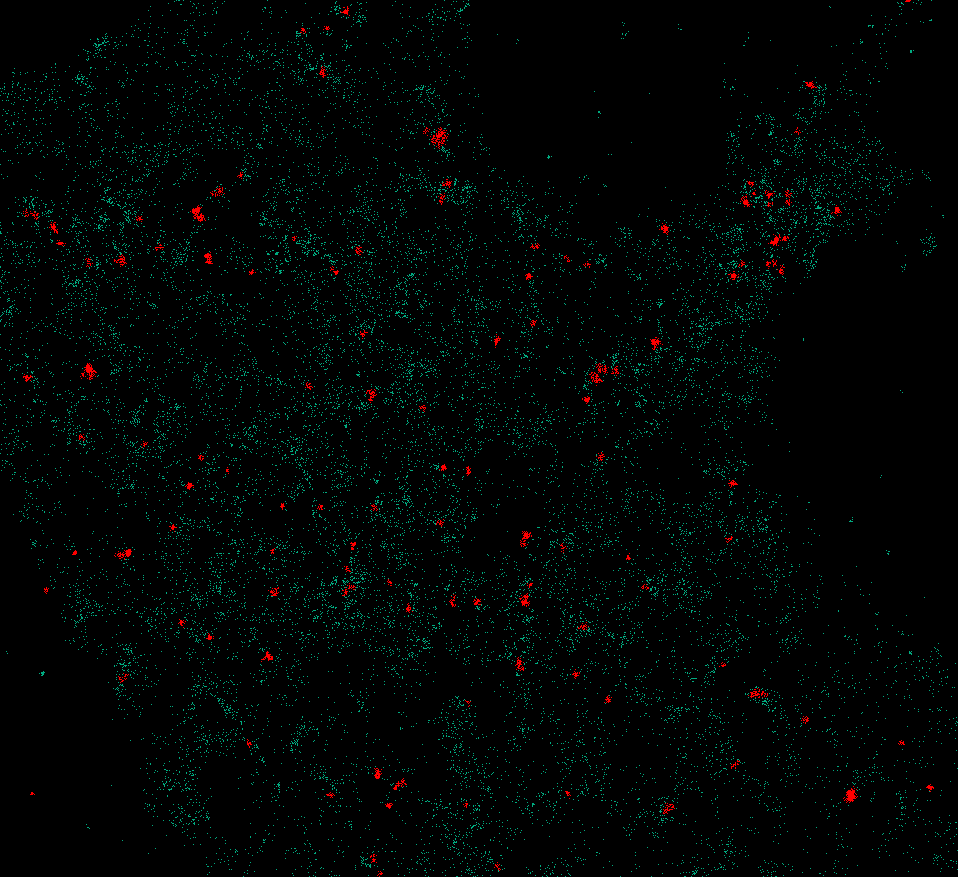

Supplement: Figure 3—source data 1. [file elife-56679-fig3-data1.zip › Figure3 - Source Data 1/DAPT/DBSCAN hotspots/DAPT_1_DBSCAN.png]

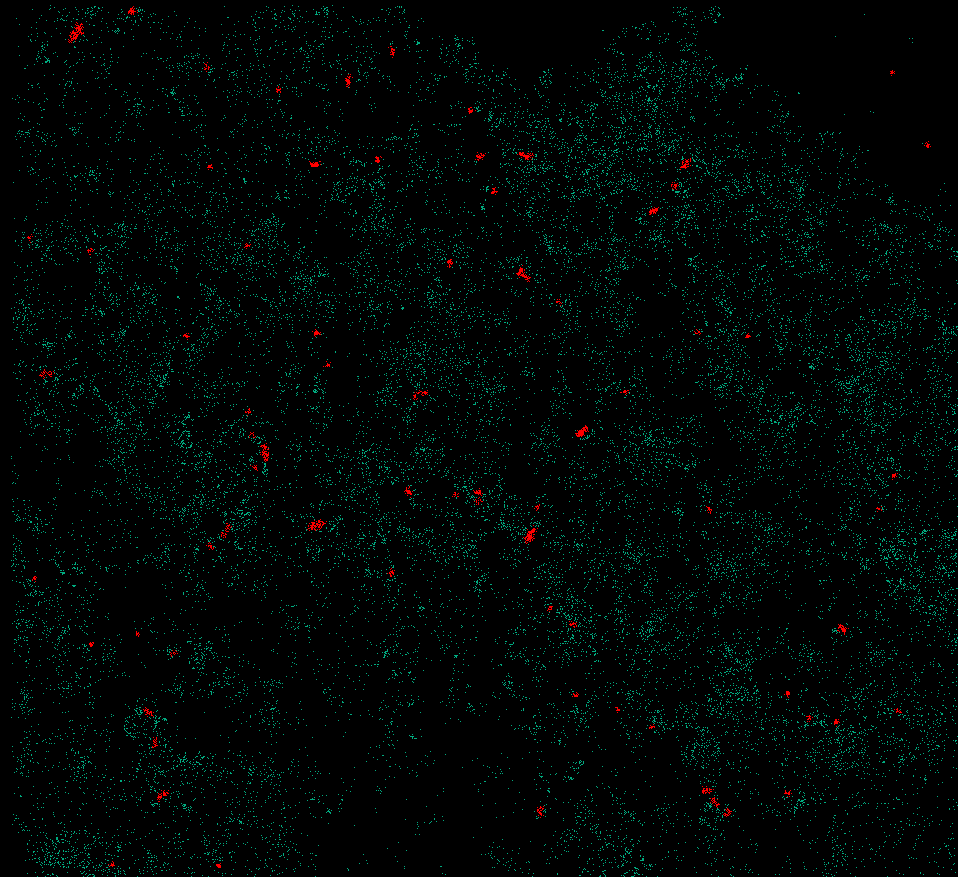

Supplement: Figure 3—source data 1. [file elife-56679-fig3-data1.zip › Figure3 - Source Data 1/DAPT/DBSCAN hotspots/DAPT_2_DBSCAN.png]

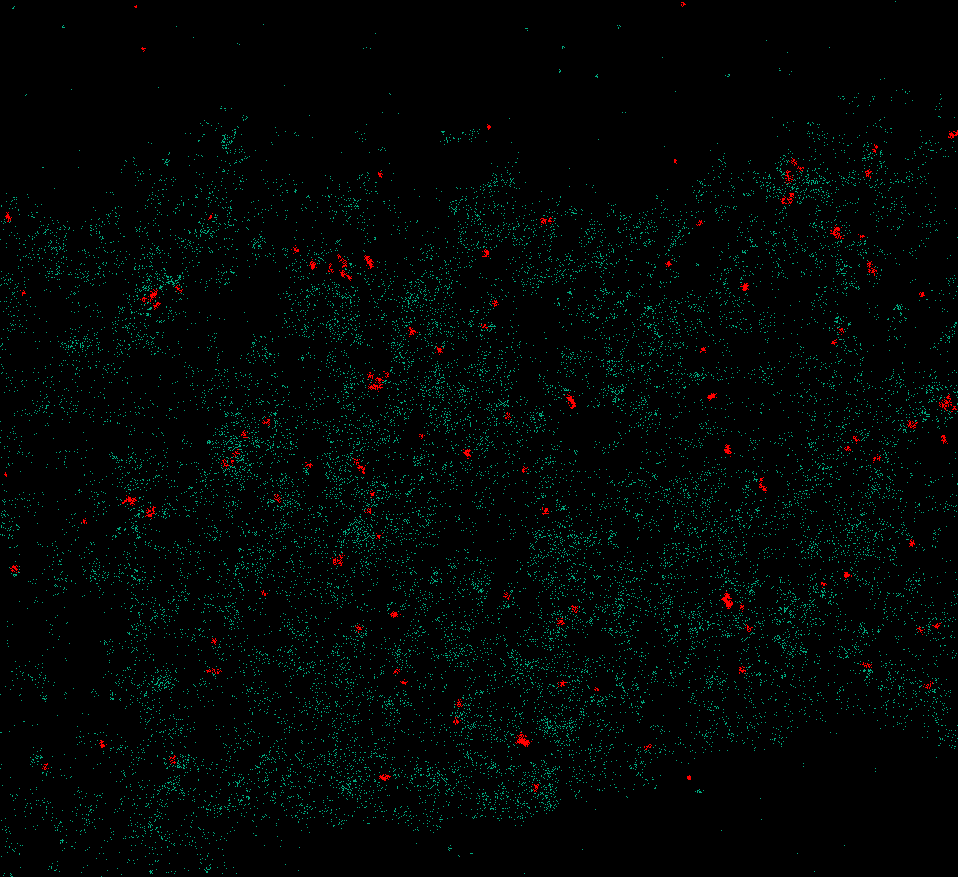

Supplement: Figure 3—source data 1. [file elife-56679-fig3-data1.zip › Figure3 - Source Data 1/DAPT/DBSCAN hotspots/DAPT_3_DBSCAN.png]

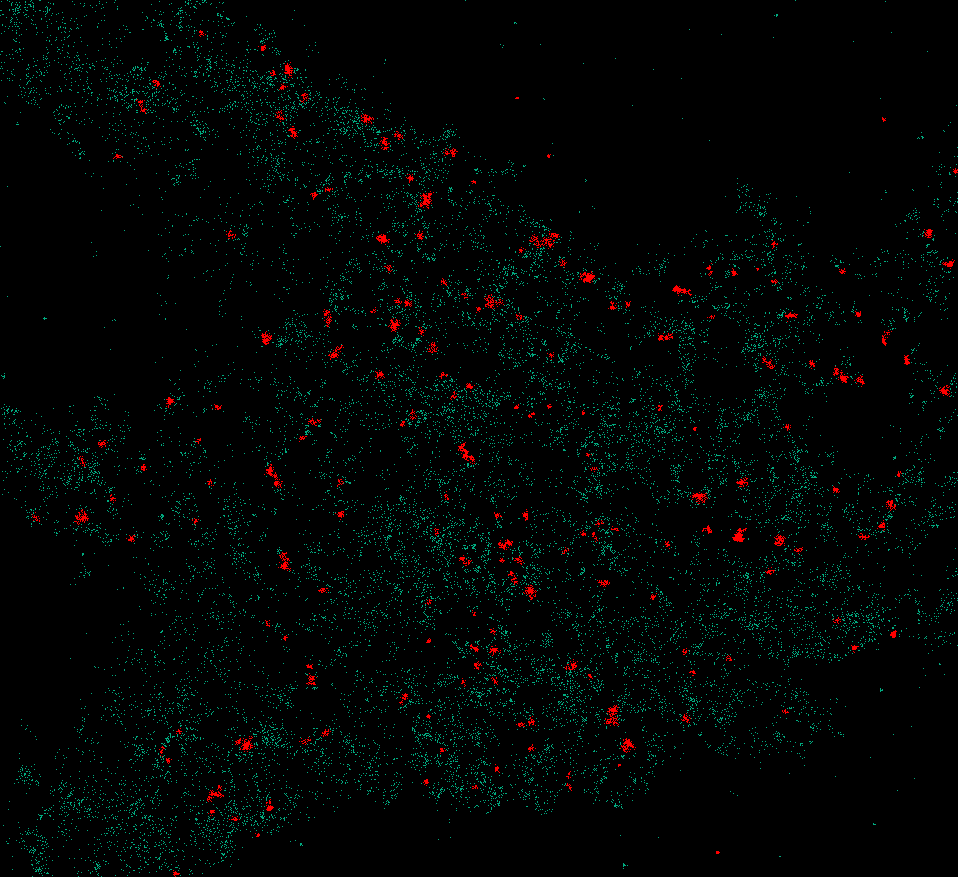

Supplement: Figure 3—source data 1. [file elife-56679-fig3-data1.zip › Figure3 - Source Data 1/DAPT/DBSCAN hotspots/DAPT_6_DBSCAN.png]

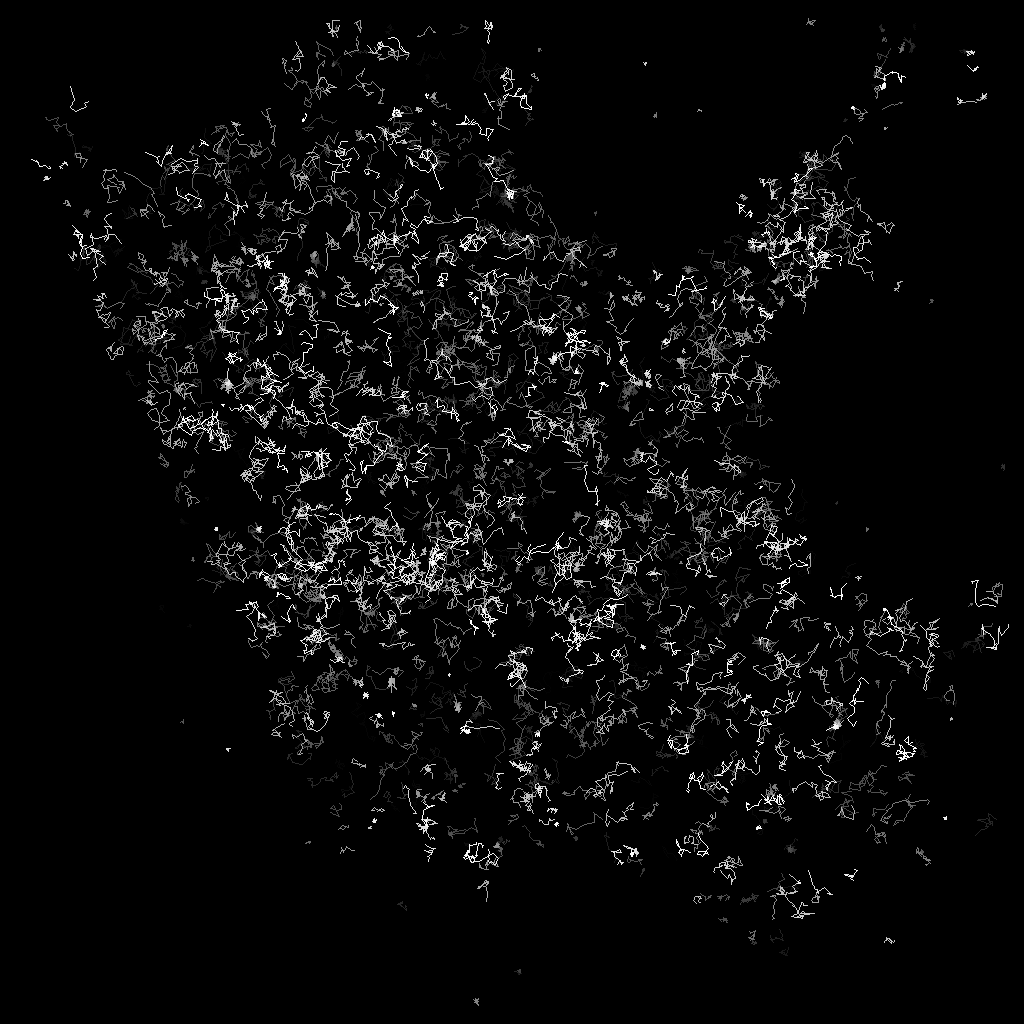

Supplement: Figure 3—source data 1. [file elife-56679-fig3-data1.zip › Figure3 - Source Data 1/DAPT/tracks/Cell_1_SPT-EOS_1-Tracks-Z4-Tracks.tif]

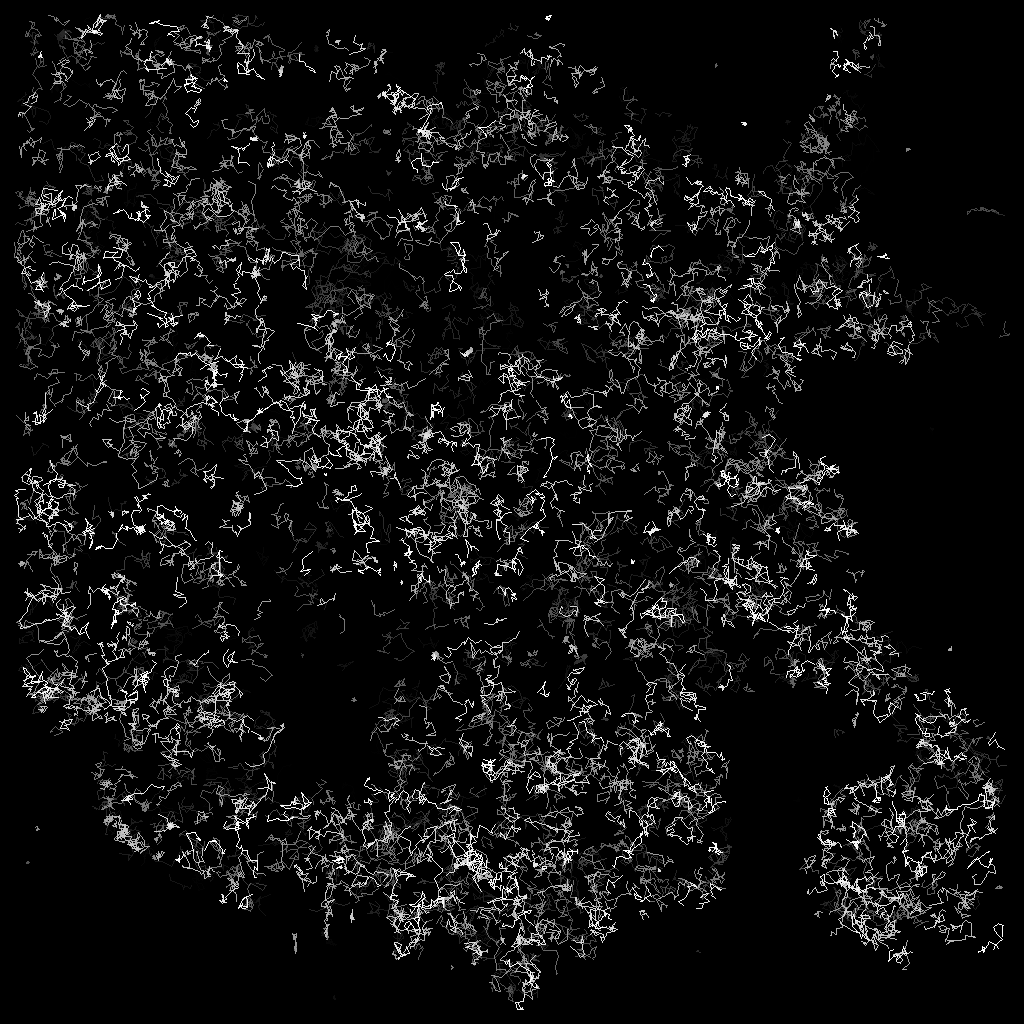

Supplement: Figure 3—source data 1. [file elife-56679-fig3-data1.zip › Figure3 - Source Data 1/DAPT/tracks/Cell_2_SPT-EOS_1-Tracks-Z4-Tracks.tif]

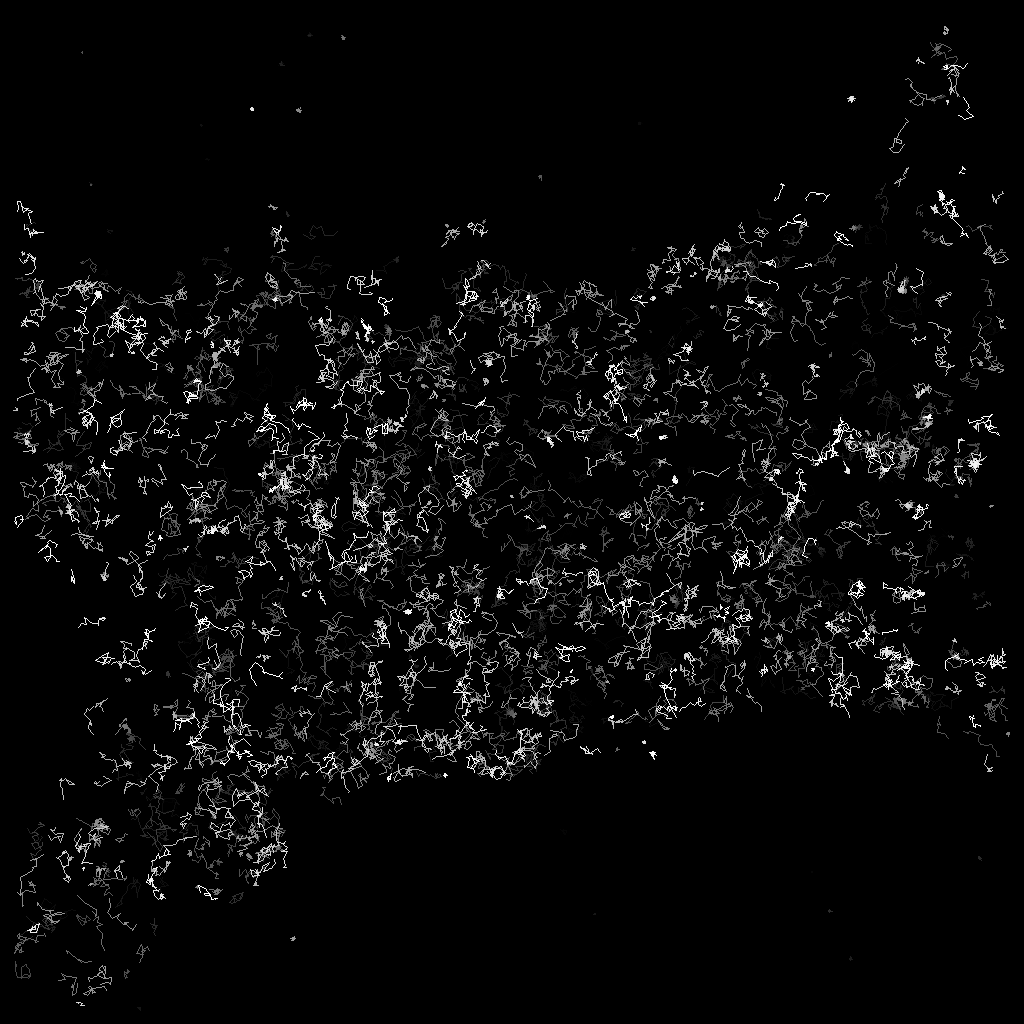

Supplement: Figure 3—source data 1. [file elife-56679-fig3-data1.zip › Figure3 - Source Data 1/DAPT/tracks/Cell_3_SPT-EOS_1-Tracks-Z4-Tracks.tif]

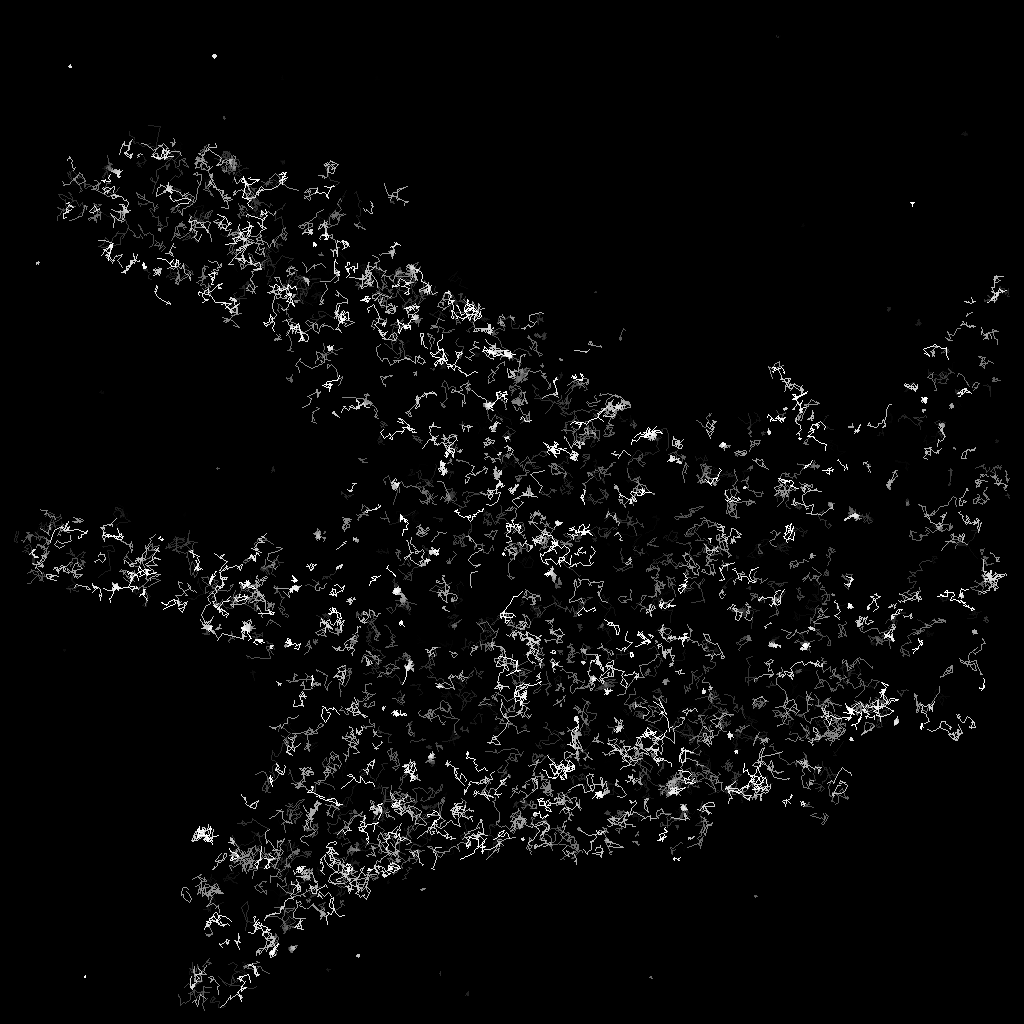

Supplement: Figure 3—source data 1. [file elife-56679-fig3-data1.zip › Figure3 - Source Data 1/DAPT/tracks/Cell_6_SPT-EOS_1-Tracks-Z4-Tracks.tif]

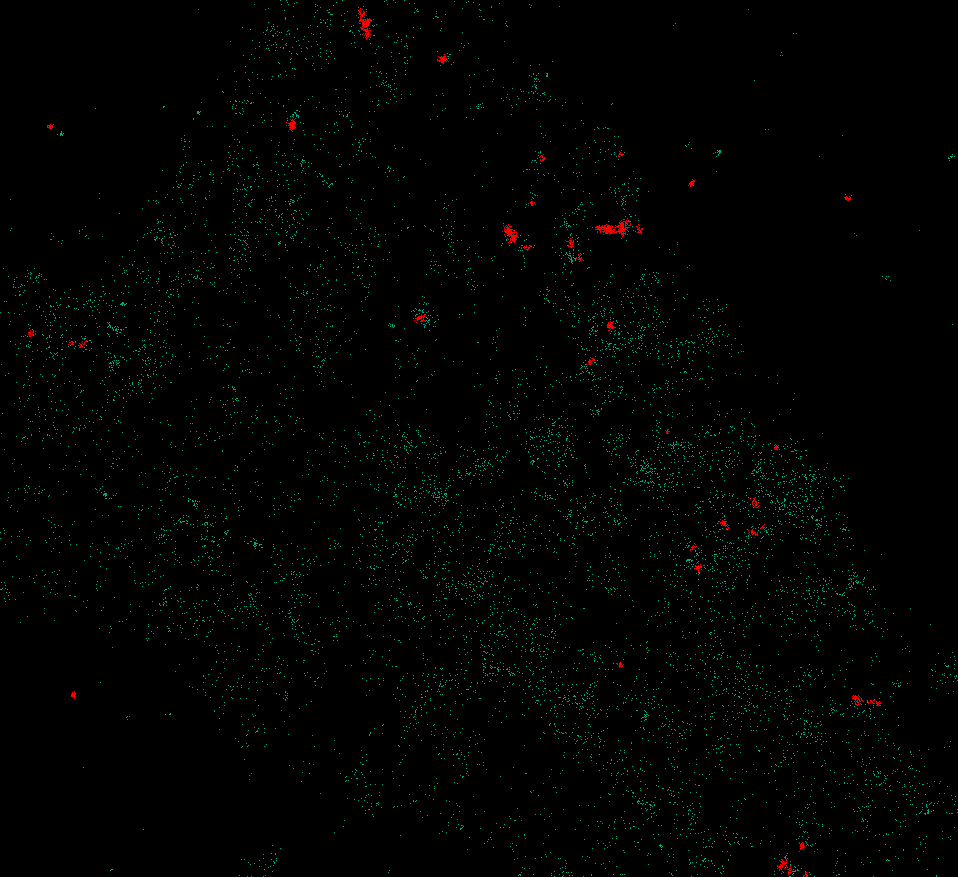

Supplement: Figure 3—source data 1. [file elife-56679-fig3-data1.zip › Figure3 - Source Data 1/DMSO/DBSCAN hotspots/DMSO_1_DBSCAN.png]

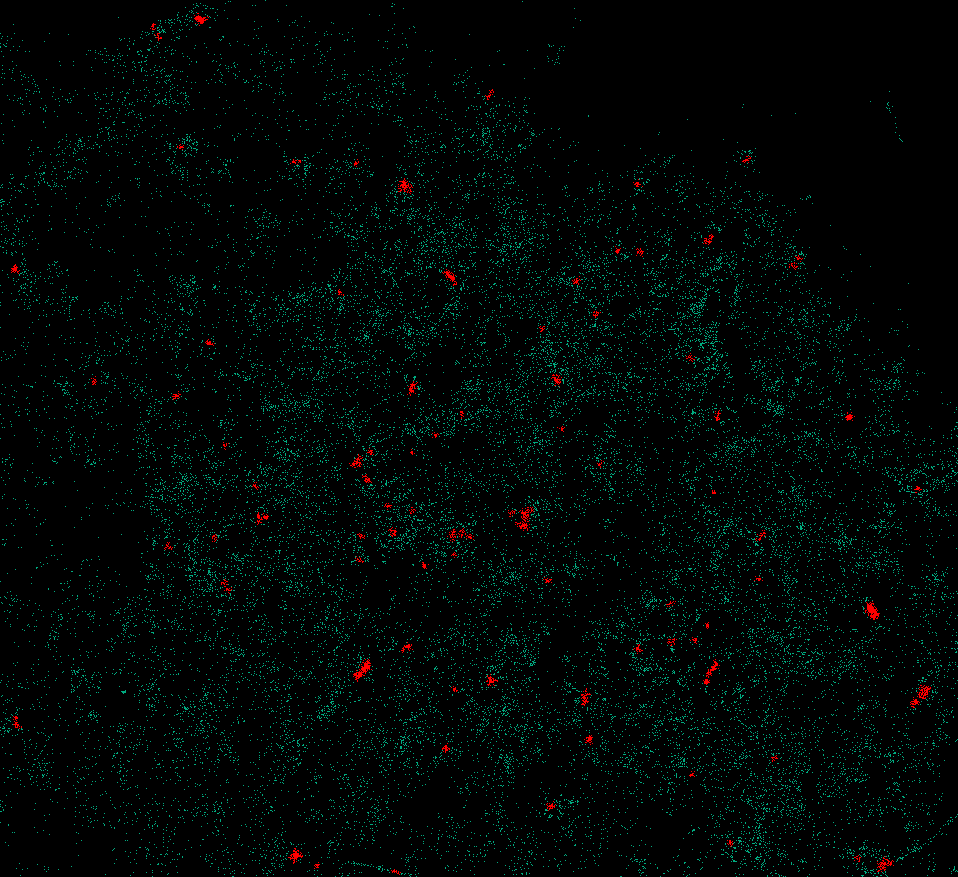

Supplement: Figure 3—source data 1. [file elife-56679-fig3-data1.zip › Figure3 - Source Data 1/DMSO/DBSCAN hotspots/DMSO_2_DBSCAN.png]

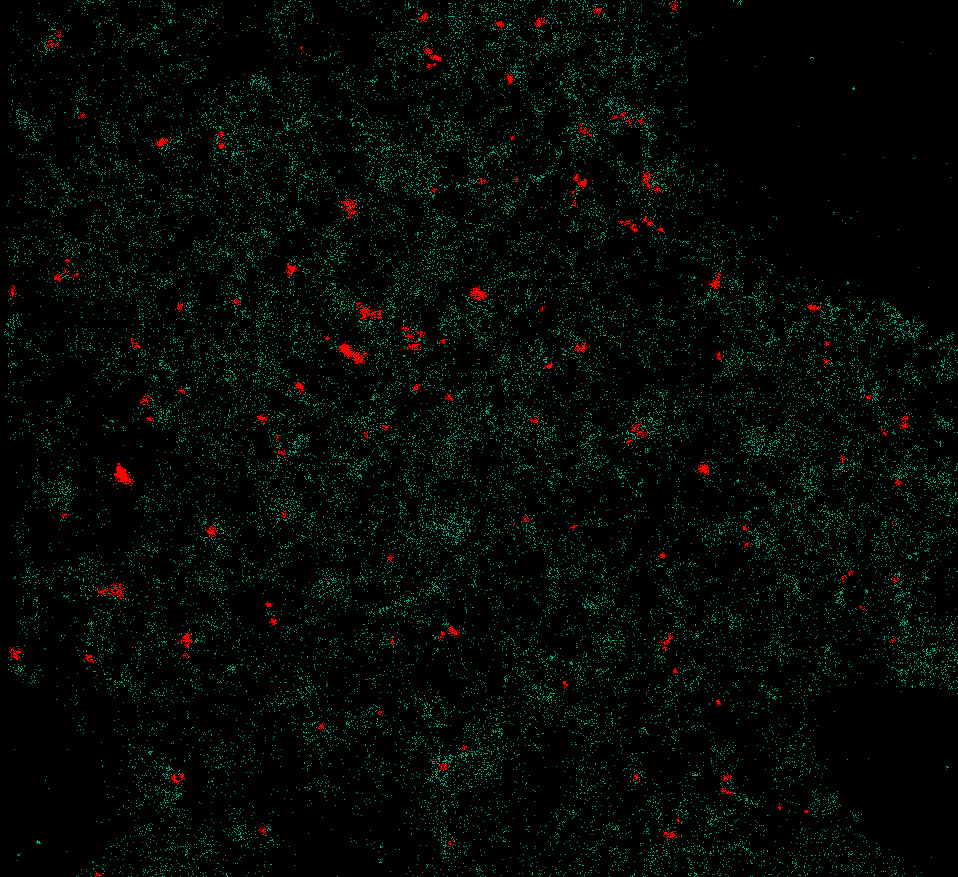

Supplement: Figure 3—source data 1. [file elife-56679-fig3-data1.zip › Figure3 - Source Data 1/DMSO/DBSCAN hotspots/DMSO_3_DBSCAN.png]

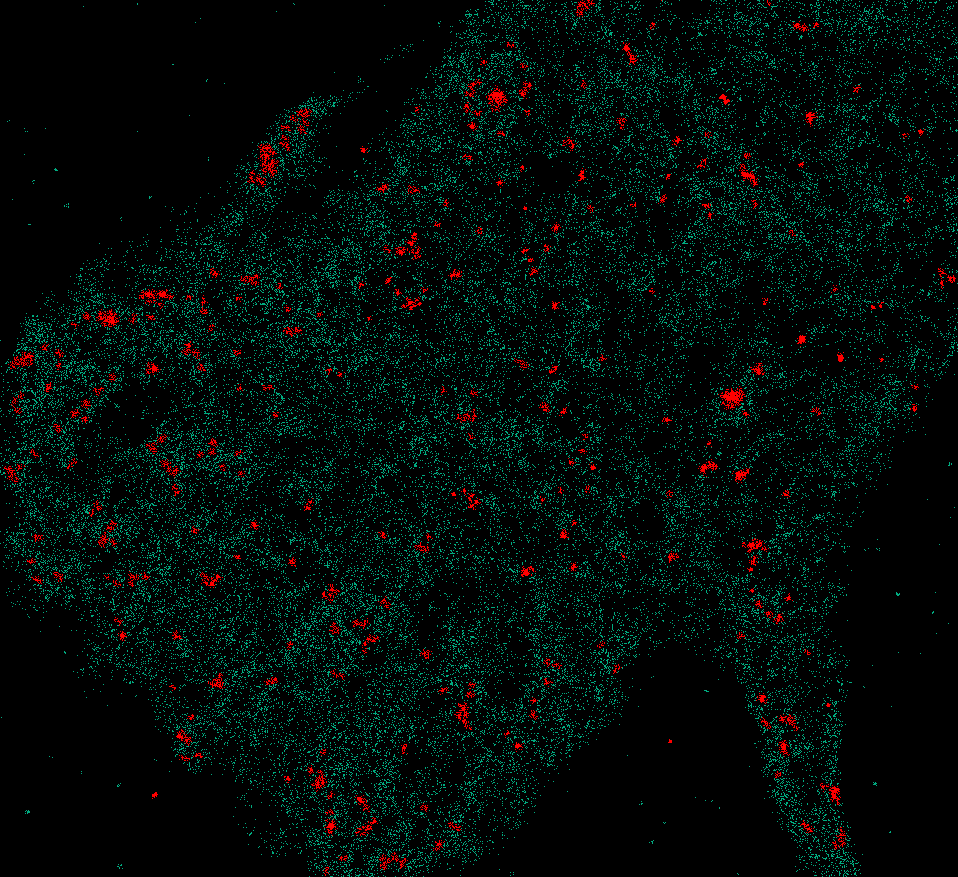

Supplement: Figure 3—source data 1. [file elife-56679-fig3-data1.zip › Figure3 - Source Data 1/DMSO/DBSCAN hotspots/DMSO_7_DBSCAN.png]

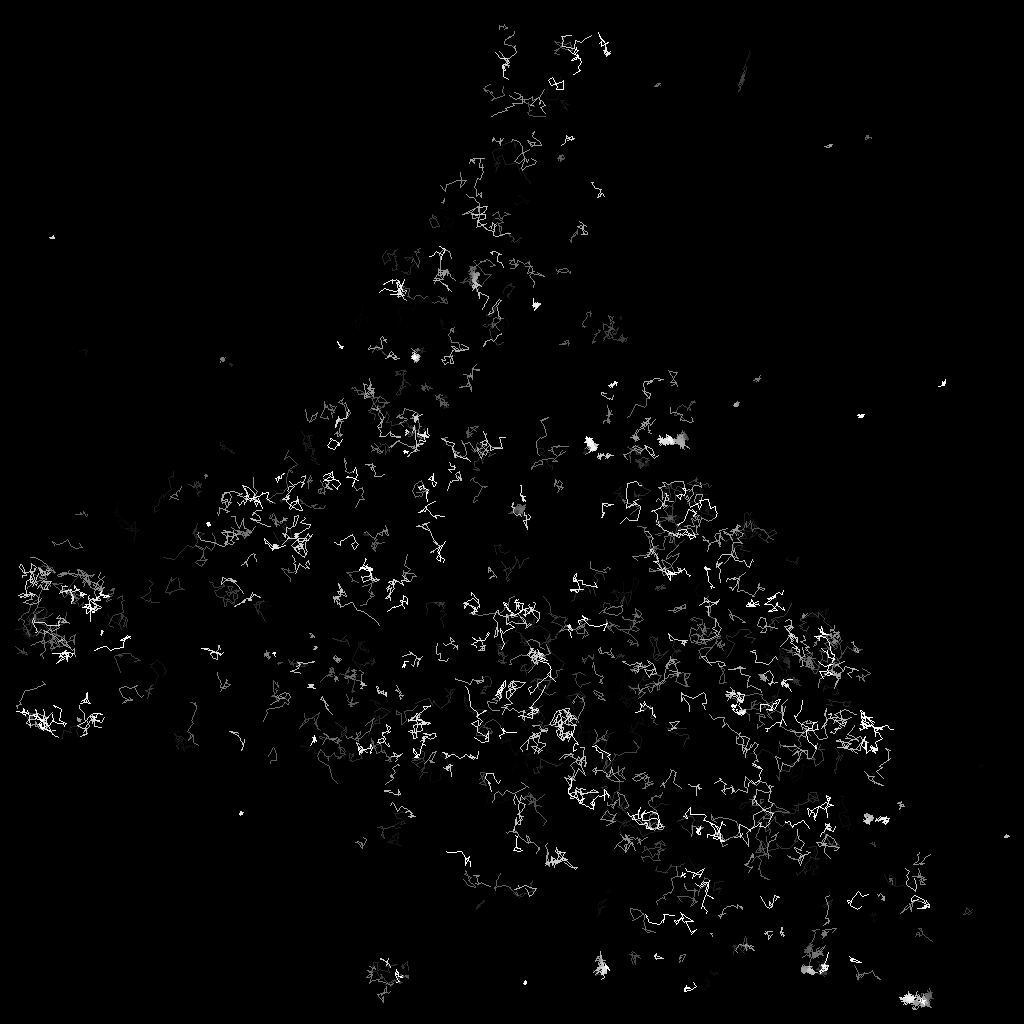

Supplement: Figure 3—source data 1. [file elife-56679-fig3-data1.zip › Figure3 - Source Data 1/DMSO/tracks/Cell_1_SPT-EOS_1-Tracks-Z4-Tracks.tif]

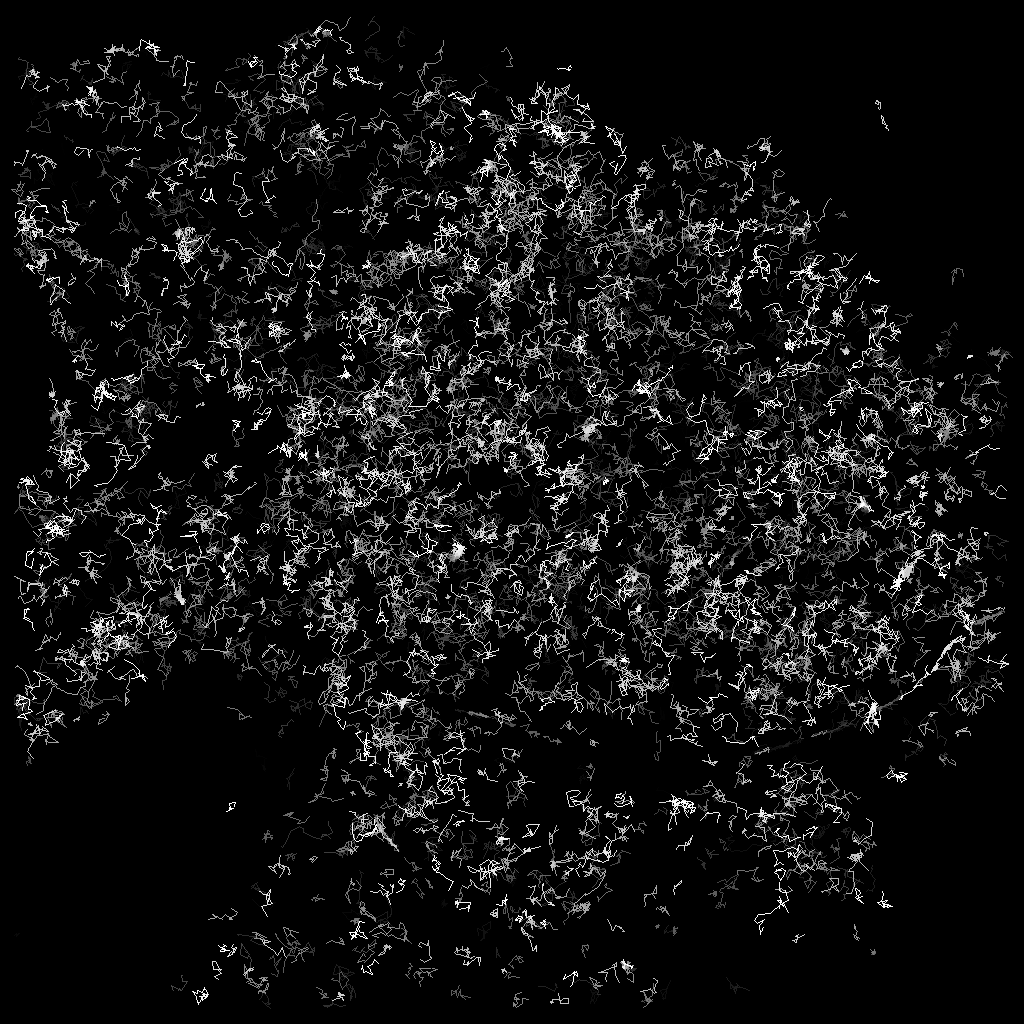

Supplement: Figure 3—source data 1. [file elife-56679-fig3-data1.zip › Figure3 - Source Data 1/DMSO/tracks/Cell_2_SPT-EOS_1-Tracks-Z4-Tracks.tif]

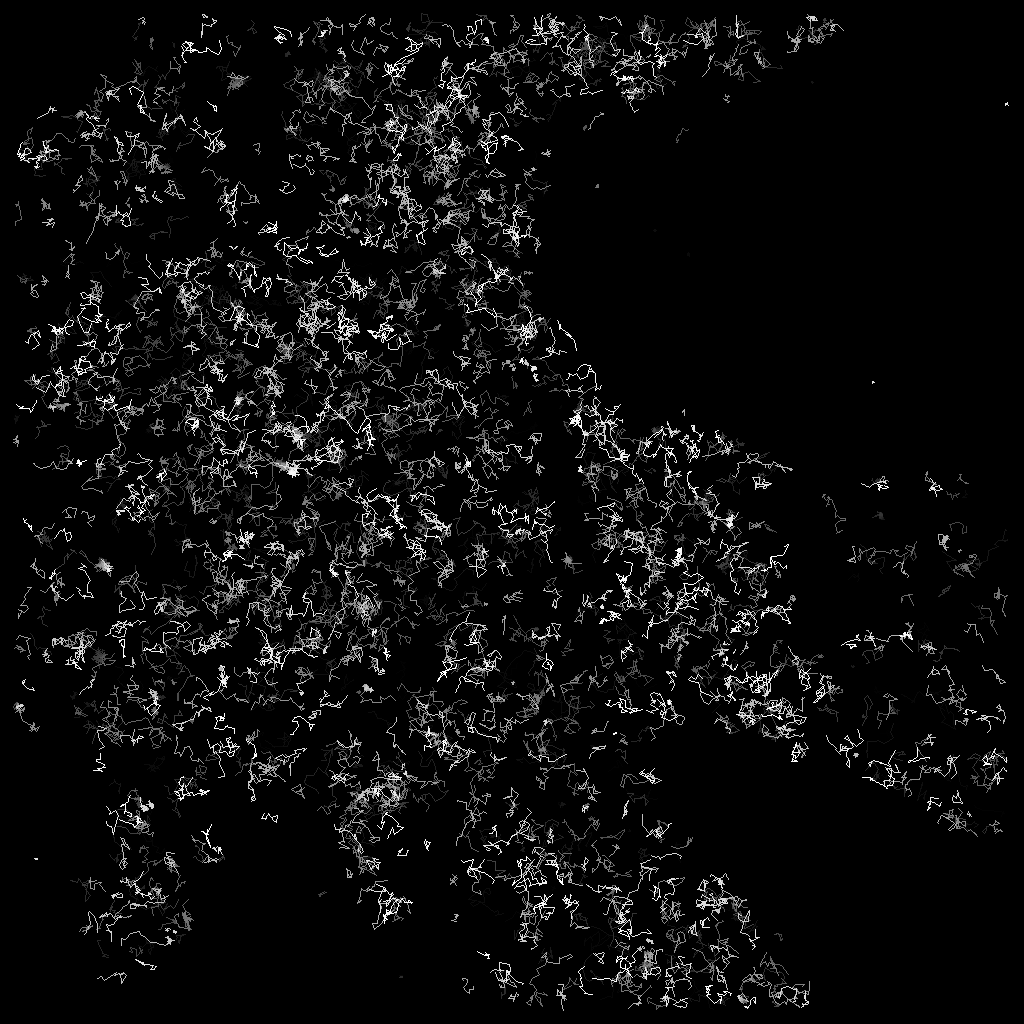

Supplement: Figure 3—source data 1. [file elife-56679-fig3-data1.zip › Figure3 - Source Data 1/DMSO/tracks/Cell_3_SPT-EOS_1-Tracks-Z4-Tracks.tif]

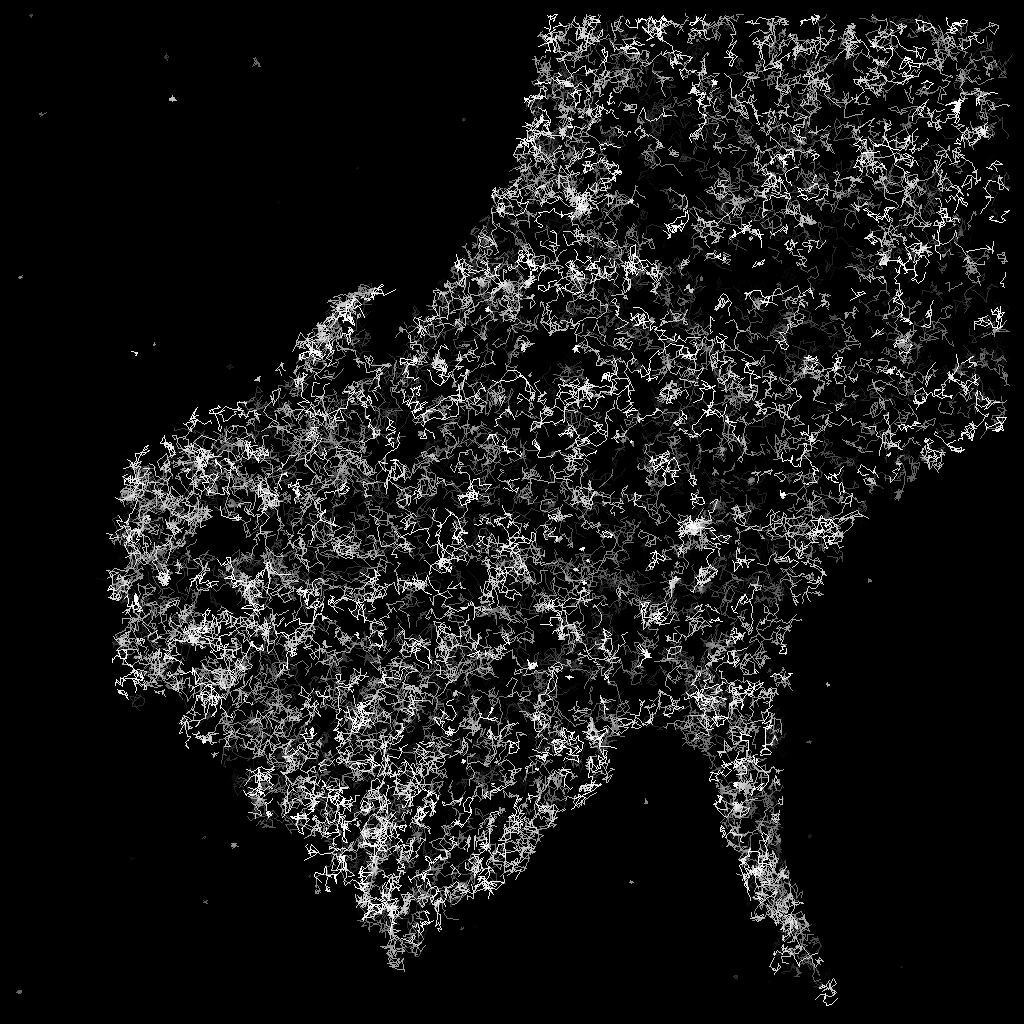

Supplement: Figure 3—source data 1. [file elife-56679-fig3-data1.zip › Figure3 - Source Data 1/DMSO/tracks/Cell_7_SPT-EOS_1-Tracks-Z4-Tracks.tif]

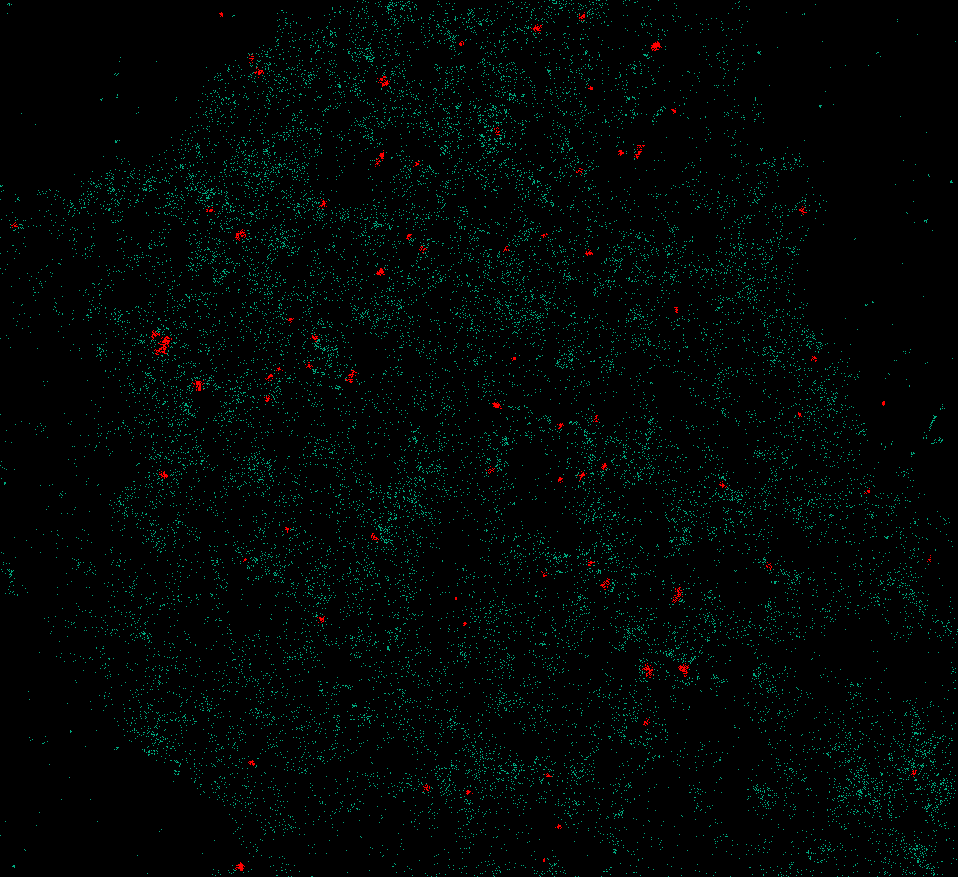

Supplement: Figure 3—source data 1. [file elife-56679-fig3-data1.zip › Figure3 - Source Data 1/InhibitorX/DBSCAN hotspots/InhX_1_DBSCAN.png]

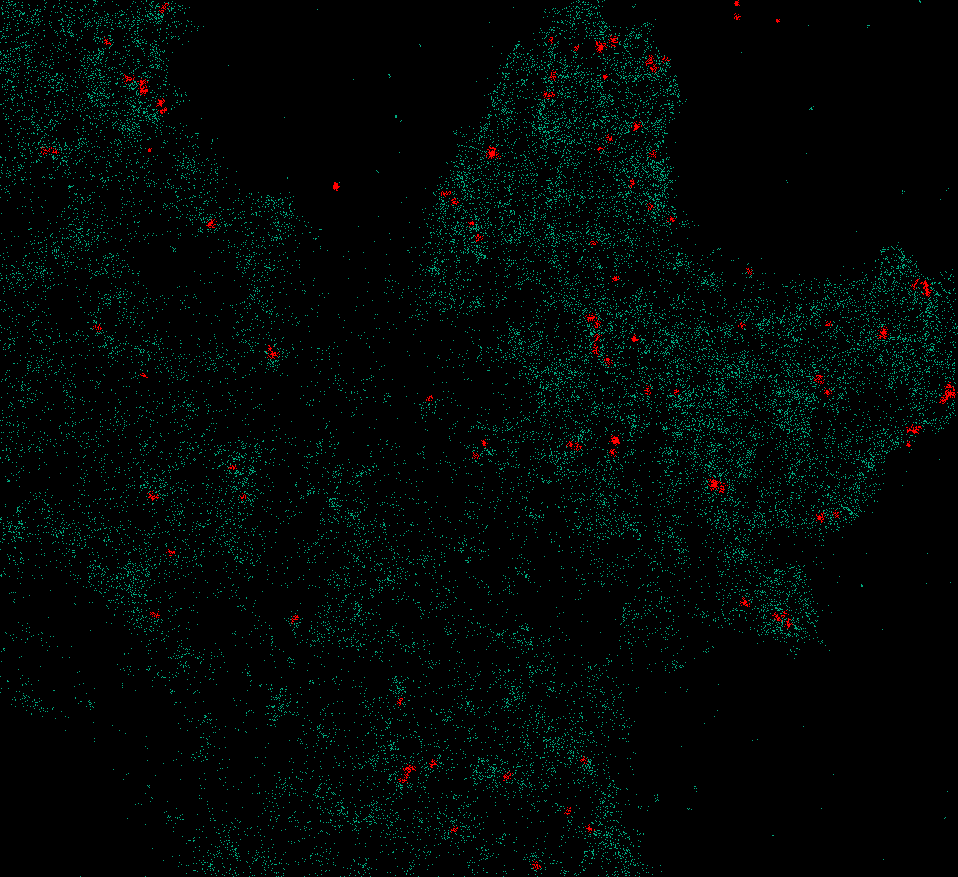

Supplement: Figure 3—source data 1. [file elife-56679-fig3-data1.zip › Figure3 - Source Data 1/InhibitorX/DBSCAN hotspots/InhX_2_DBSCAN.png]

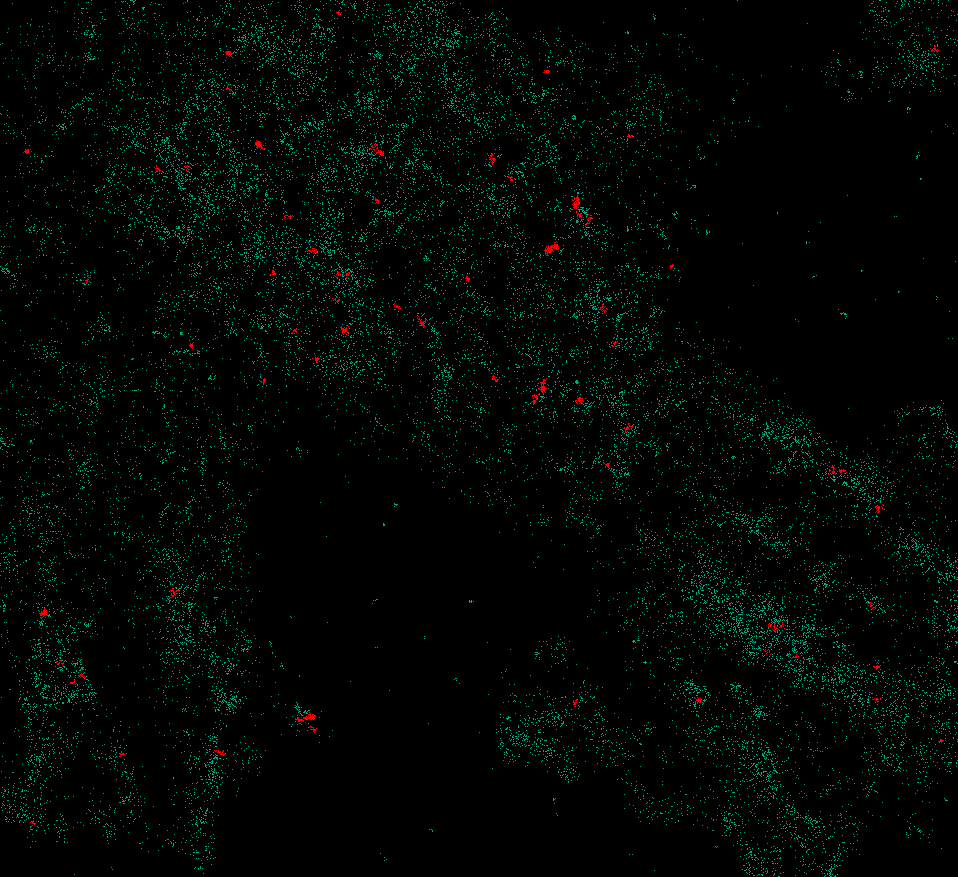

Supplement: Figure 3—source data 1. [file elife-56679-fig3-data1.zip › Figure3 - Source Data 1/InhibitorX/DBSCAN hotspots/InhX_5_DBSCAN.png]

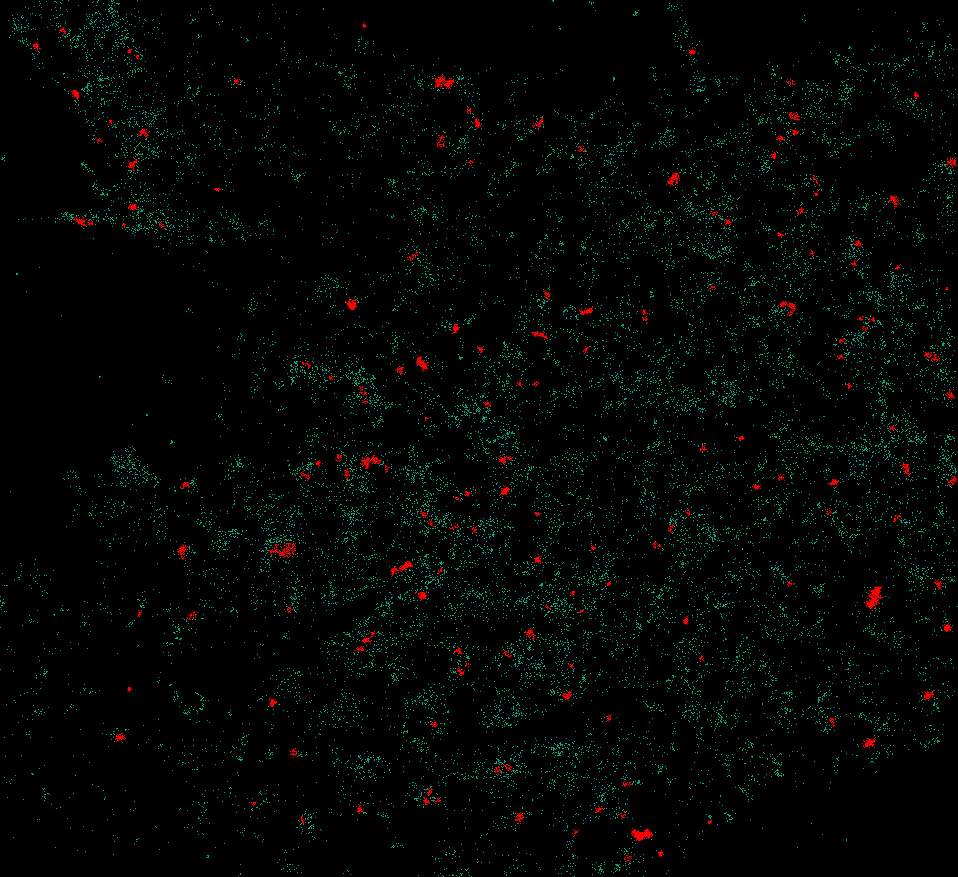

Supplement: Figure 3—source data 1. [file elife-56679-fig3-data1.zip › Figure3 - Source Data 1/InhibitorX/DBSCAN hotspots/InhX_7_DBSCAN.png]

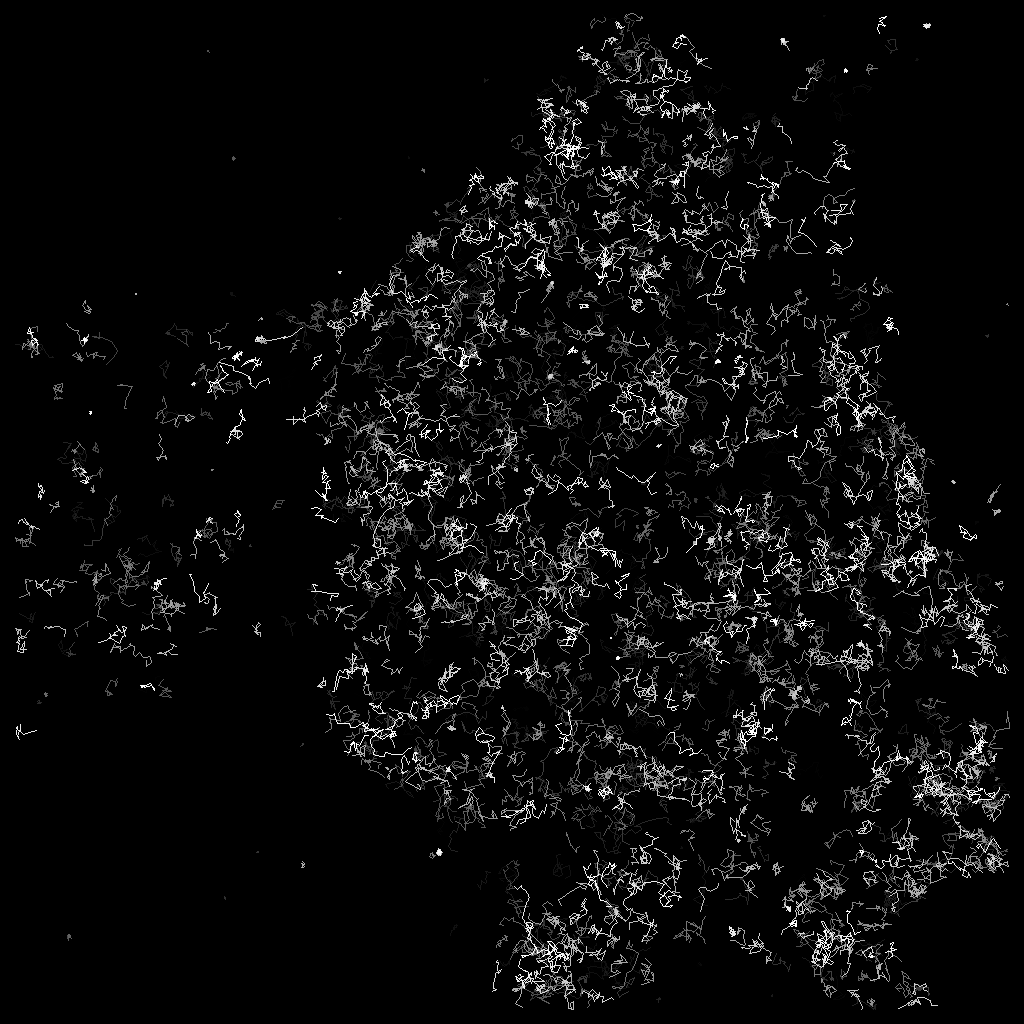

Supplement: Figure 3—source data 1. [file elife-56679-fig3-data1.zip › Figure3 - Source Data 1/InhibitorX/tracks/Cell_1_SPT-EOS_1-Tracks-Z4-Tracks.tif]

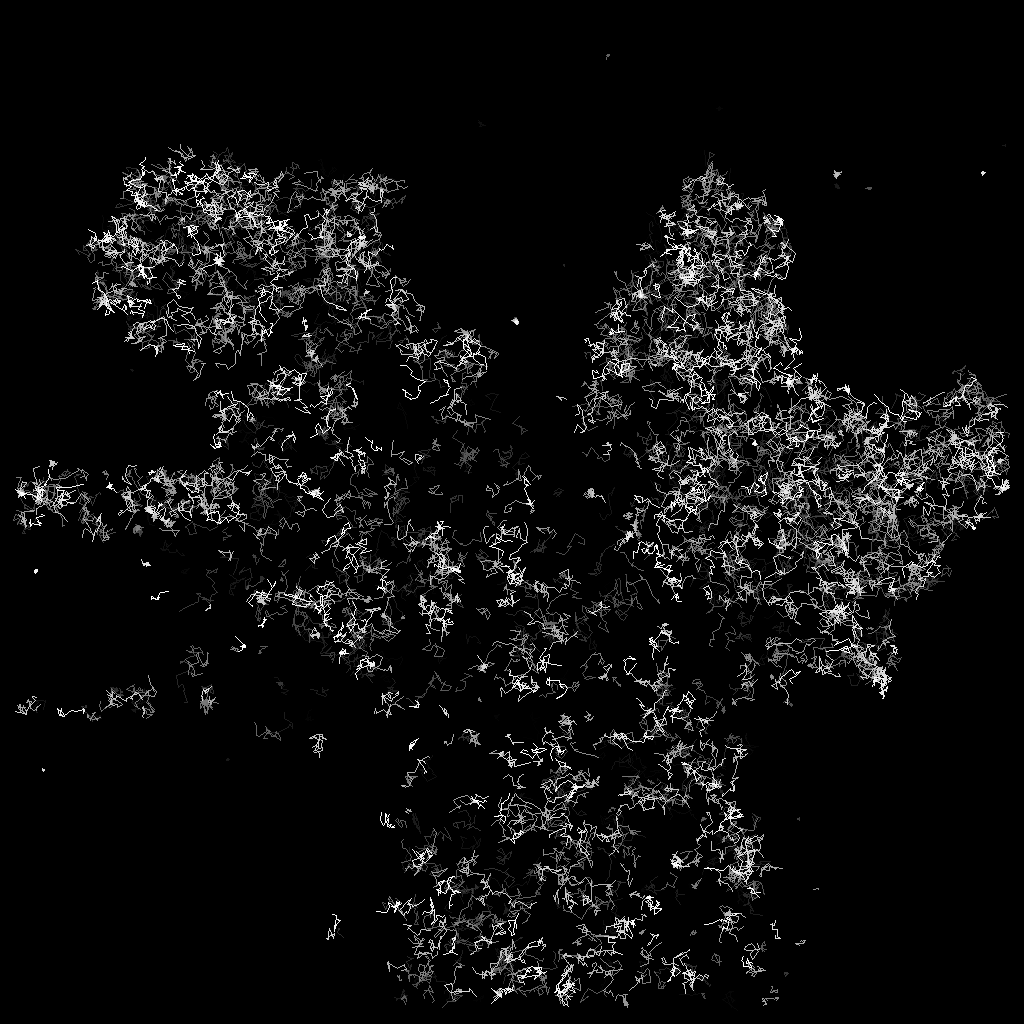

Supplement: Figure 3—source data 1. [file elife-56679-fig3-data1.zip › Figure3 - Source Data 1/InhibitorX/tracks/Cell_2_SPT-EOS_1-Tracks-Z4-Tracks.tif]

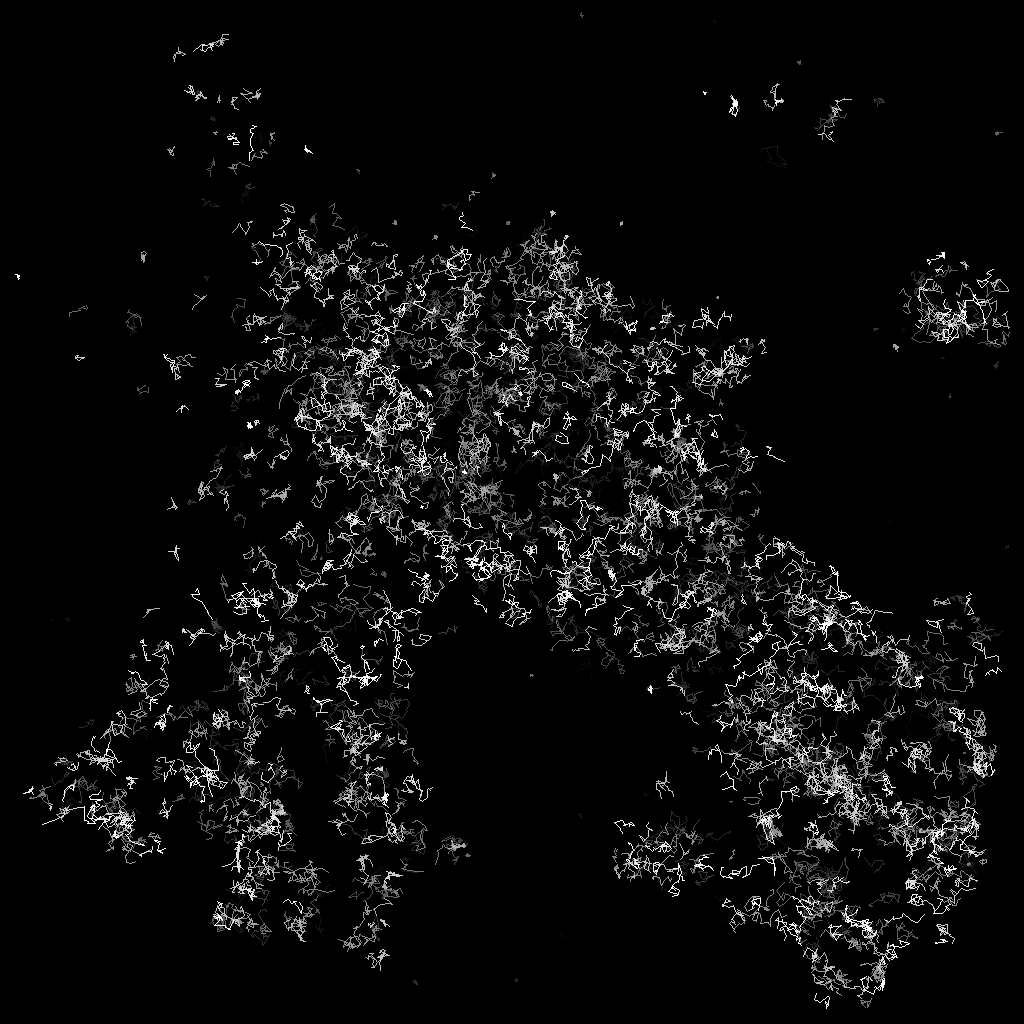

Supplement: Figure 3—source data 1. [file elife-56679-fig3-data1.zip › Figure3 - Source Data 1/InhibitorX/tracks/Cell_5_SPT-EOS_1-Tracks-Z4-Tracks.tif]

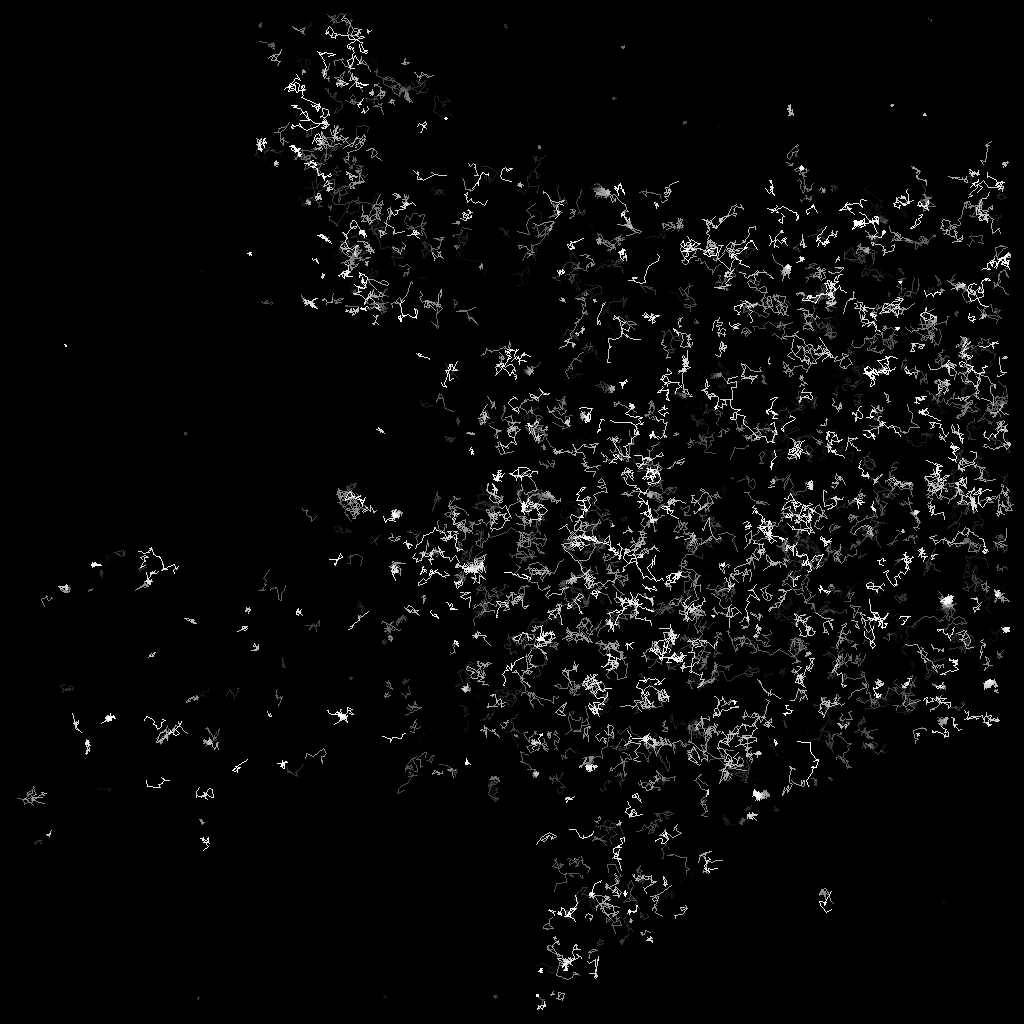

Supplement: Figure 3—source data 1. [file elife-56679-fig3-data1.zip › Figure3 - Source Data 1/InhibitorX/tracks/Cell_7_SPT-EOS_1-Tracks-Z4-Tracks.tif]

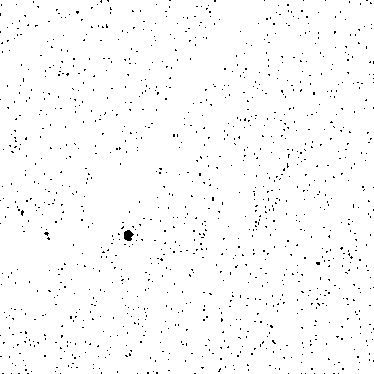

Supplement: Figure 4—source data 1. [file elife-56679-fig4-data1.zip › Figure4 - Source Data1/GFP-PSEN1 ADAM10/roi masks/21_PS-1.tif - watershed (h=1404,00, T=4213,00, %=20, n=1157).tif]

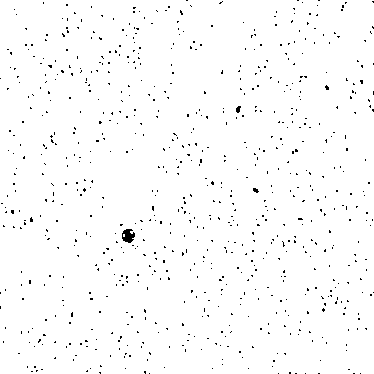

Supplement: Figure 4—source data 1. [file elife-56679-fig4-data1.zip › Figure4 - Source Data1/GFP-PSEN1 ADAM10/roi masks/22_A10-1.tif - watershed (h=1404,00, T=4213,00, %=20, n=759).tif]

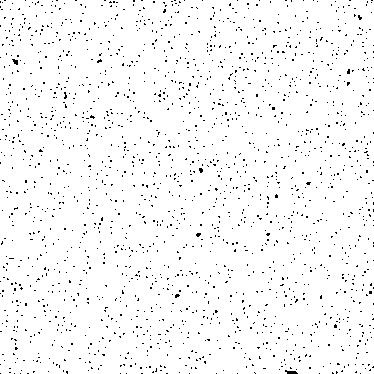

Supplement: Figure 4—source data 1. [file elife-56679-fig4-data1.zip › Figure4 - Source Data1/GFP-PSEN1 ADAM10/roi masks/23_PS-1.tif - watershed (h=1404,00, T=4213,00, %=20, n=1891).tif]

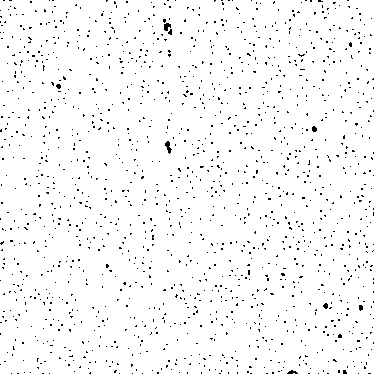

Supplement: Figure 4—source data 1. [file elife-56679-fig4-data1.zip › Figure4 - Source Data1/GFP-PSEN1 ADAM10/roi masks/24_A10-1.tif - watershed (h=1404,00, T=4213,00, %=20, n=1491).tif]

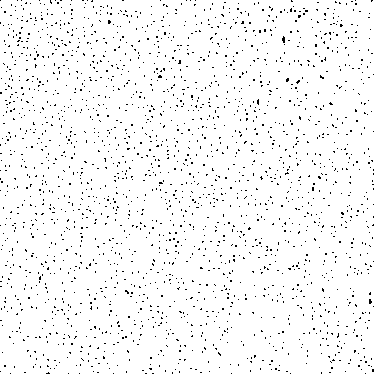

Supplement: Figure 4—source data 1. [file elife-56679-fig4-data1.zip › Figure4 - Source Data1/GFP-PSEN1 ADAM10/roi masks/25_PS-1.tif - watershed (h=1404,00, T=4213,00, %=20, n=2204).tif]

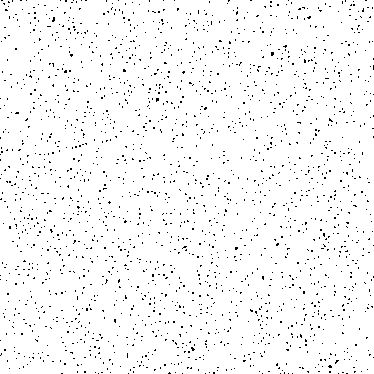

Supplement: Figure 4—source data 1. [file elife-56679-fig4-data1.zip › Figure4 - Source Data1/GFP-PSEN1 ADAM10/roi masks/25_PS-2.tif - watershed (h=1404,00, T=4213,00, %=20, n=2057).tif]

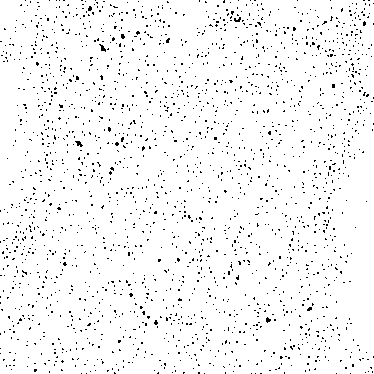

Supplement: Figure 4—source data 1. [file elife-56679-fig4-data1.zip › Figure4 - Source Data1/GFP-PSEN1 ADAM10/roi masks/25_PS-3.tif - watershed (h=1404,00, T=4213,00, %=20, n=2118).tif]

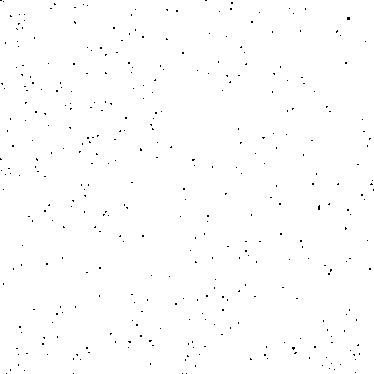

Supplement: Figure 4—source data 1. [file elife-56679-fig4-data1.zip › Figure4 - Source Data1/GFP-PSEN1 ADAM10/roi masks/26_A10-1.tif - watershed (h=1404,00, T=4213,00, %=20, n=359).tif]

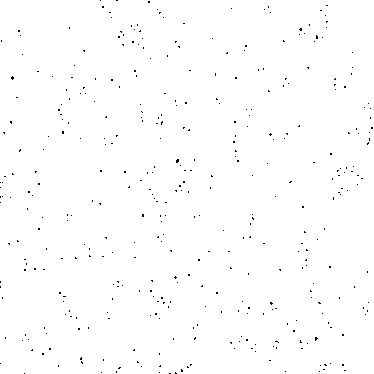

Supplement: Figure 4—source data 1. [file elife-56679-fig4-data1.zip › Figure4 - Source Data1/GFP-PSEN1 ADAM10/roi masks/26_A10-2.tif - watershed (h=1404,00, T=4213,00, %=20, n=387).tif]

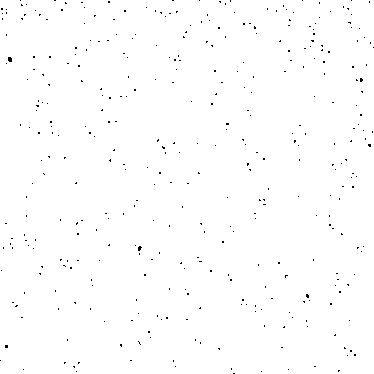

Supplement: Figure 4—source data 1. [file elife-56679-fig4-data1.zip › Figure4 - Source Data1/GFP-PSEN1 ADAM10/roi masks/26_A10-3.tif - watershed (h=1404,00, T=4213,00, %=20, n=373).tif]

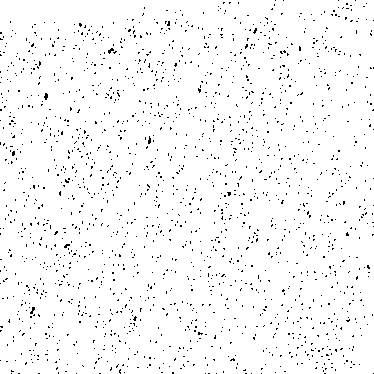

Supplement: Figure 4—source data 1. [file elife-56679-fig4-data1.zip › Figure4 - Source Data1/GFP-PSEN1 ADAM10/roi masks/27_PS-1.tif - watershed (h=1404,00, T=4213,00, %=20, n=1833).tif]

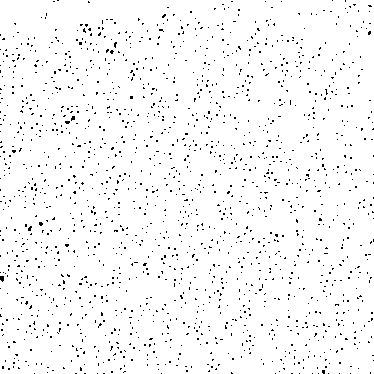

Supplement: Figure 4—source data 1. [file elife-56679-fig4-data1.zip › Figure4 - Source Data1/GFP-PSEN1 ADAM10/roi masks/28_A10-1.tif - watershed (h=1404,00, T=4213,00, %=20, n=1684).tif]

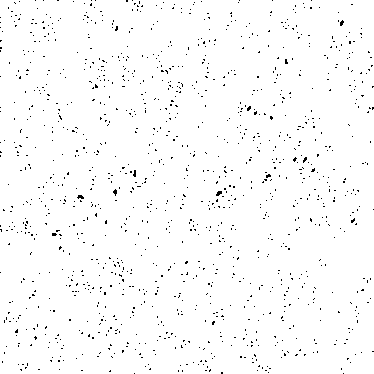

Supplement: Figure 4—source data 1. [file elife-56679-fig4-data1.zip › Figure4 - Source Data1/GFP-PSEN1 ADAM10/roi masks/29-PS-1.tif - watershed (h=1404,00, T=4213,00, %=20, n=1231).tif]

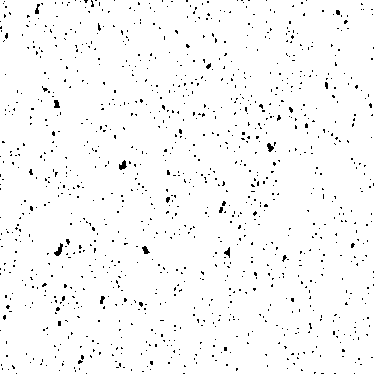

Supplement: Figure 4—source data 1. [file elife-56679-fig4-data1.zip › Figure4 - Source Data1/GFP-PSEN1 ADAM10/roi masks/30_A10-1.tif - watershed (h=1404,00, T=4213,00, %=20, n=1095).tif]

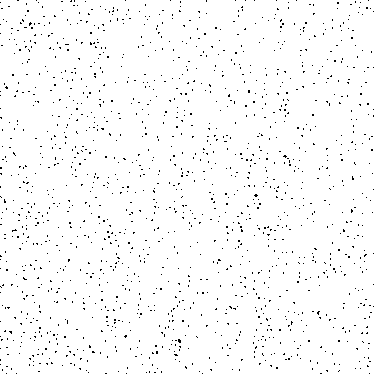

Supplement: Figure 4—source data 1. [file elife-56679-fig4-data1.zip › Figure4 - Source Data1/GFP-PSEN1 ADAM10/roi masks/31_PS-1.tif - watershed (h=1404,00, T=4213,00, %=20, n=1506).tif]

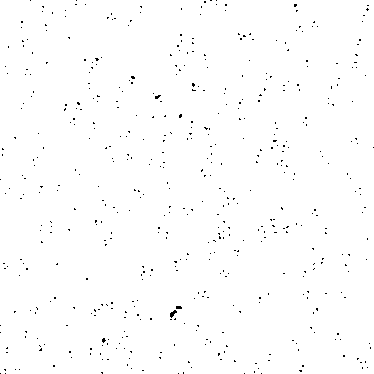

Supplement: Figure 4—source data 1. [file elife-56679-fig4-data1.zip › Figure4 - Source Data1/GFP-PSEN1 ADAM10/roi masks/32_A10-1.tif - watershed (h=1404,00, T=4213,00, %=20, n=510).tif]

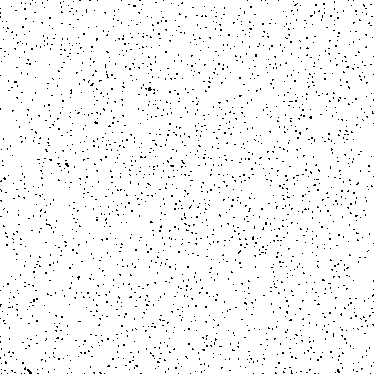

Supplement: Figure 4—source data 1. [file elife-56679-fig4-data1.zip › Figure4 - Source Data1/GFP-PSEN1 ADAM10/roi masks/33_PS-1.tif - watershed (h=1404,00, T=4213,00, %=20, n=2195).tif]

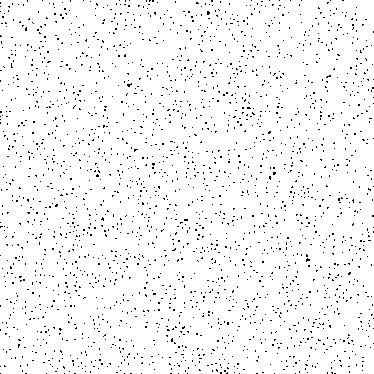

Supplement: Figure 4—source data 1. [file elife-56679-fig4-data1.zip › Figure4 - Source Data1/GFP-PSEN1 ADAM10/roi masks/33_PS-2.tif - watershed (h=1404,00, T=4213,00, %=20, n=2370).tif]

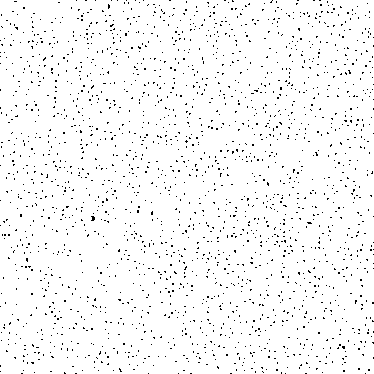

Supplement: Figure 4—source data 1. [file elife-56679-fig4-data1.zip › Figure4 - Source Data1/GFP-PSEN1 ADAM10/roi masks/33_PS-3.tif - watershed (h=1404,00, T=4213,00, %=20, n=2115).tif]

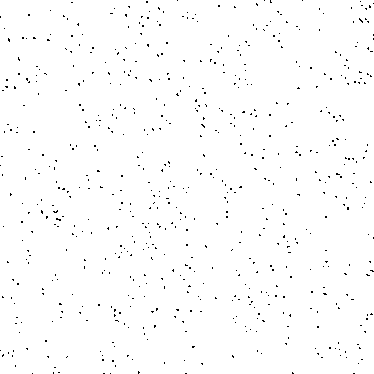

Supplement: Figure 4—source data 1. [file elife-56679-fig4-data1.zip › Figure4 - Source Data1/GFP-PSEN1 ADAM10/roi masks/34_A10-1.tif - watershed (h=1404,00, T=4213,00, %=20, n=646).tif]

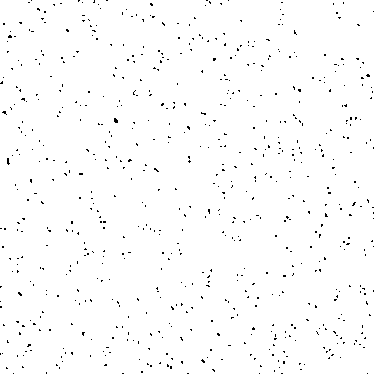

Supplement: Figure 4—source data 1. [file elife-56679-fig4-data1.zip › Figure4 - Source Data1/GFP-PSEN1 ADAM10/roi masks/34_A10-2.tif - watershed (h=1404,00, T=4213,00, %=20, n=674).tif]

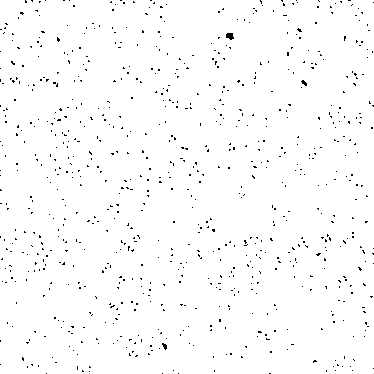

Supplement: Figure 4—source data 1. [file elife-56679-fig4-data1.zip › Figure4 - Source Data1/GFP-PSEN1 ADAM10/roi masks/34_A10-3.tif - watershed (h=1404,00, T=4213,00, %=20, n=803).tif]

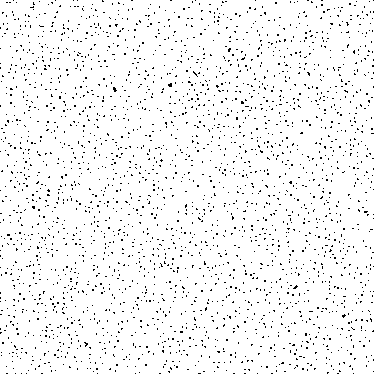

Supplement: Figure 4—source data 1. [file elife-56679-fig4-data1.zip › Figure4 - Source Data1/GFP-PSEN1 ADAM10/roi masks/35_PS-1.tif - watershed (h=1404,00, T=4213,00, %=20, n=2656).tif]

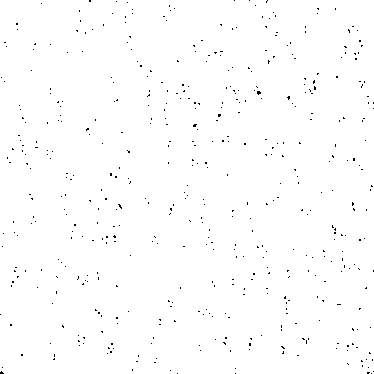

Supplement: Figure 4—source data 1. [file elife-56679-fig4-data1.zip › Figure4 - Source Data1/GFP-PSEN1 ADAM10/roi masks/36_A10-1.tif - watershed (h=1404,00, T=4213,00, %=20, n=481).tif]

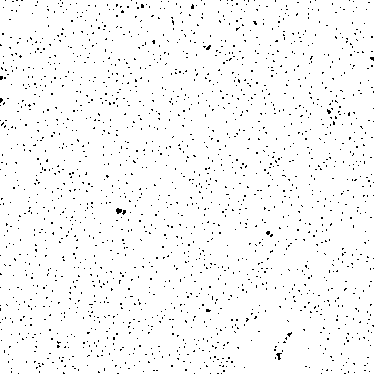

Supplement: Figure 4—source data 1. [file elife-56679-fig4-data1.zip › Figure4 - Source Data1/GFP-PSEN1 ADAM10/roi masks/37_PS-1.tif - watershed (h=1404,00, T=4213,00, %=20, n=1850).tif]

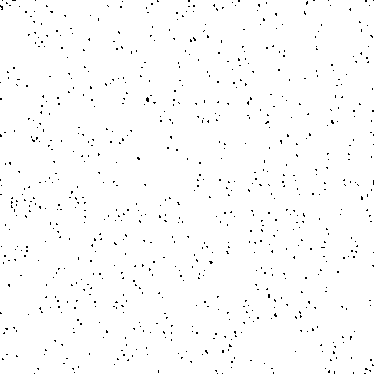

Supplement: Figure 4—source data 1. [file elife-56679-fig4-data1.zip › Figure4 - Source Data1/GFP-PSEN1 ADAM10/roi masks/38_A10-1.tif - watershed (h=1404,00, T=4213,00, %=20, n=743).tif]

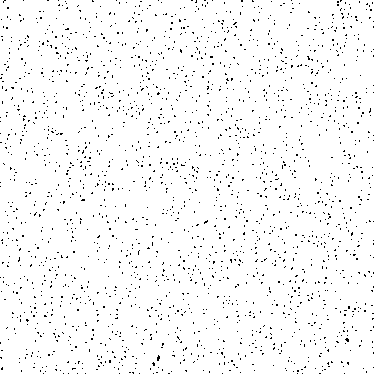

Supplement: Figure 4—source data 1. [file elife-56679-fig4-data1.zip › Figure4 - Source Data1/GFP-PSEN1 ADAM10/roi masks/39_PS-1.tif - watershed (h=1404,00, T=4213,00, %=20, n=2121).tif]

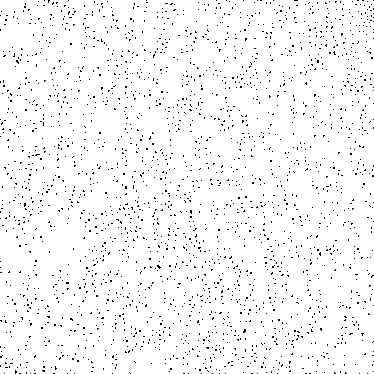

Supplement: Figure 4—source data 1. [file elife-56679-fig4-data1.zip › Figure4 - Source Data1/GFP-PSEN1 ADAM10/roi masks/39_PS-2.tif - watershed (h=1404,00, T=4213,00, %=20, n=2408).tif]

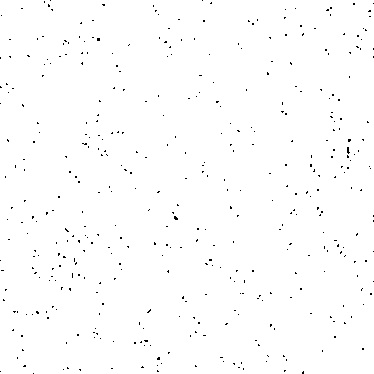

Supplement: Figure 4—source data 1. [file elife-56679-fig4-data1.zip › Figure4 - Source Data1/GFP-PSEN1 ADAM10/roi masks/40_A10-1.tif - watershed (h=1404,00, T=4213,00, %=20, n=417).tif]

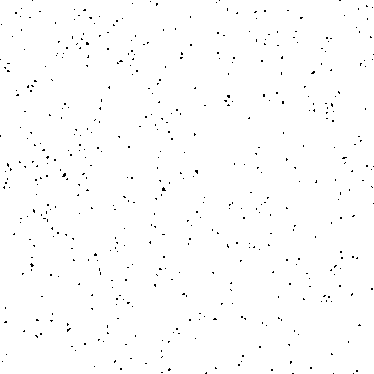

Supplement: Figure 4—source data 1. [file elife-56679-fig4-data1.zip › Figure4 - Source Data1/GFP-PSEN1 ADAM10/roi masks/40_A10-2.tif - watershed (h=1404,00, T=4213,00, %=20, n=484).tif]

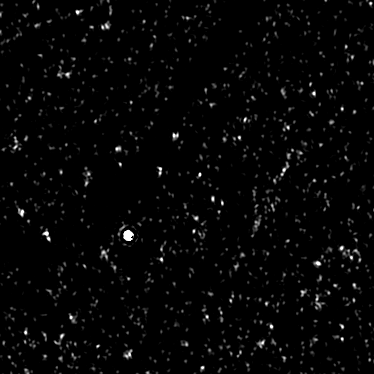

Supplement: Figure 4—source data 1. [file elife-56679-fig4-data1.zip › Figure4 - Source Data1/GFP-PSEN1 ADAM10/rois/21_PS-1.tif]

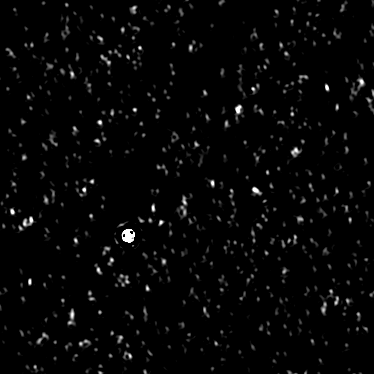

Supplement: Figure 4—source data 1. [file elife-56679-fig4-data1.zip › Figure4 - Source Data1/GFP-PSEN1 ADAM10/rois/22_A10-1.tif]

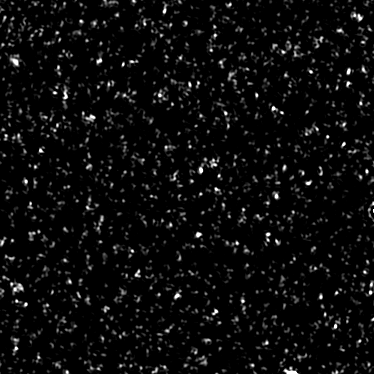

Supplement: Figure 4—source data 1. [file elife-56679-fig4-data1.zip › Figure4 - Source Data1/GFP-PSEN1 ADAM10/rois/23_PS-1.tif]

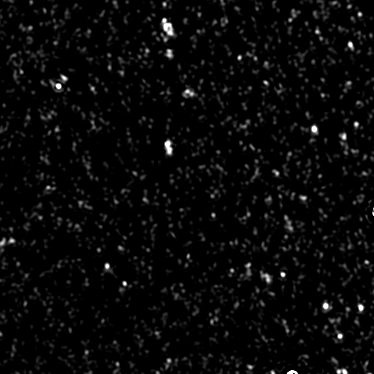

Supplement: Figure 4—source data 1. [file elife-56679-fig4-data1.zip › Figure4 - Source Data1/GFP-PSEN1 ADAM10/rois/24_A10-1.tif]

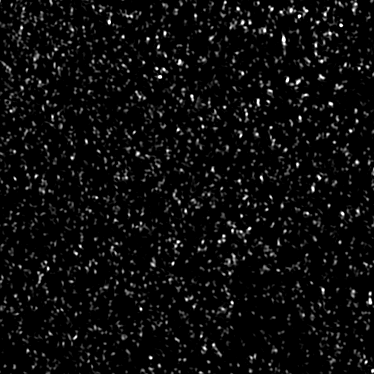

Supplement: Figure 4—source data 1. [file elife-56679-fig4-data1.zip › Figure4 - Source Data1/GFP-PSEN1 ADAM10/rois/25_PS-1.tif]

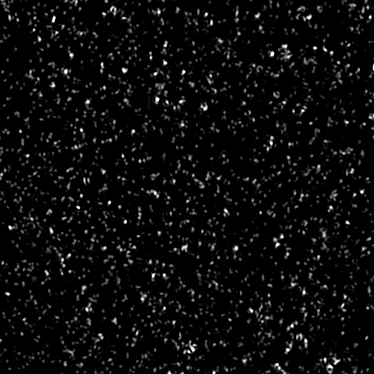

Supplement: Figure 4—source data 1. [file elife-56679-fig4-data1.zip › Figure4 - Source Data1/GFP-PSEN1 ADAM10/rois/25_PS-2.tif]

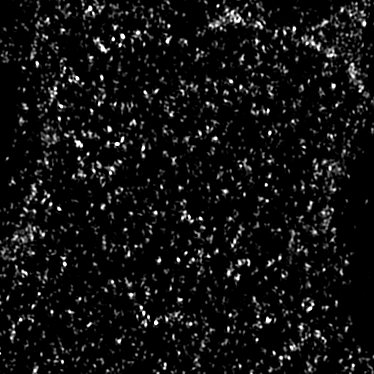

Supplement: Figure 4—source data 1. [file elife-56679-fig4-data1.zip › Figure4 - Source Data1/GFP-PSEN1 ADAM10/rois/25_PS-3.tif]

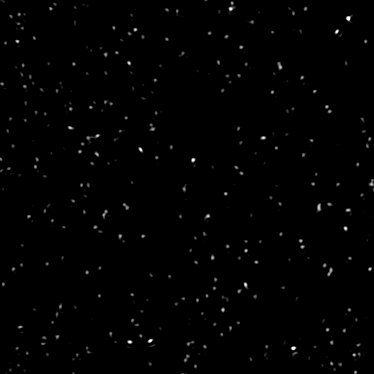

Supplement: Figure 4—source data 1. [file elife-56679-fig4-data1.zip › Figure4 - Source Data1/GFP-PSEN1 ADAM10/rois/26_A10-1.tif]

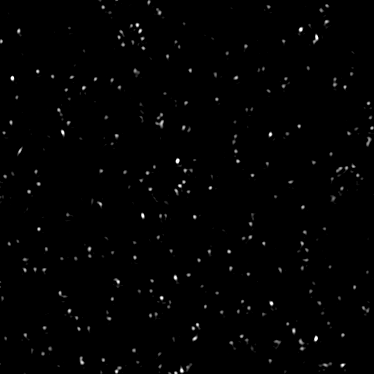

Supplement: Figure 4—source data 1. [file elife-56679-fig4-data1.zip › Figure4 - Source Data1/GFP-PSEN1 ADAM10/rois/26_A10-2.tif]

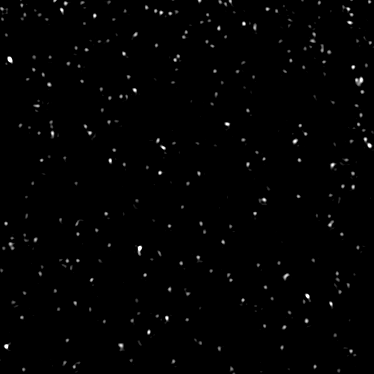

Supplement: Figure 4—source data 1. [file elife-56679-fig4-data1.zip › Figure4 - Source Data1/GFP-PSEN1 ADAM10/rois/26_A10-3.tif]

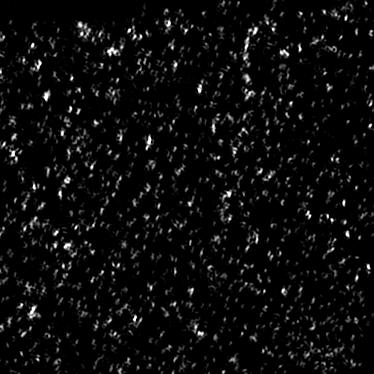

Supplement: Figure 4—source data 1. [file elife-56679-fig4-data1.zip › Figure4 - Source Data1/GFP-PSEN1 ADAM10/rois/27_PS-1.tif]

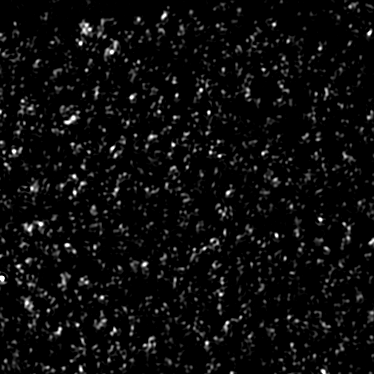

Supplement: Figure 4—source data 1. [file elife-56679-fig4-data1.zip › Figure4 - Source Data1/GFP-PSEN1 ADAM10/rois/28_A10-1.tif]

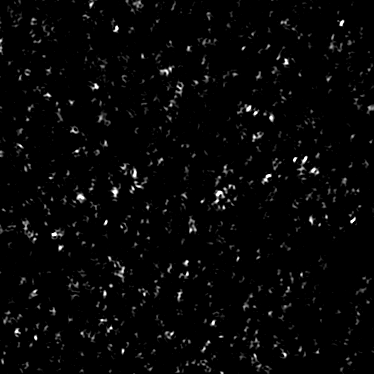

Supplement: Figure 4—source data 1. [file elife-56679-fig4-data1.zip › Figure4 - Source Data1/GFP-PSEN1 ADAM10/rois/29-PS-1.tif]

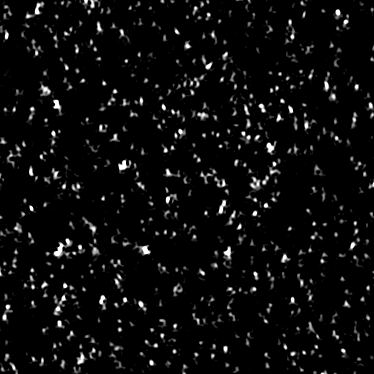

Supplement: Figure 4—source data 1. [file elife-56679-fig4-data1.zip › Figure4 - Source Data1/GFP-PSEN1 ADAM10/rois/30_A10-1.tif]

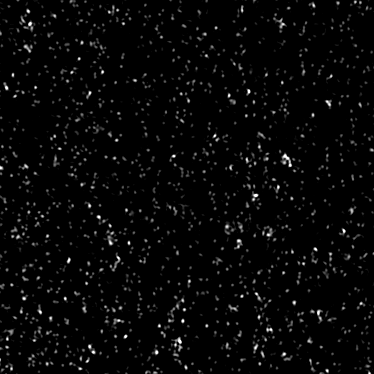

Supplement: Figure 4—source data 1. [file elife-56679-fig4-data1.zip › Figure4 - Source Data1/GFP-PSEN1 ADAM10/rois/31_PS-1.tif]

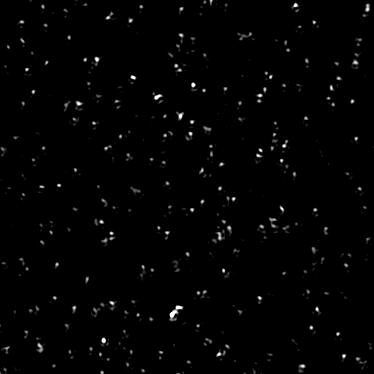

Supplement: Figure 4—source data 1. [file elife-56679-fig4-data1.zip › Figure4 - Source Data1/GFP-PSEN1 ADAM10/rois/32_A10-1.tif]

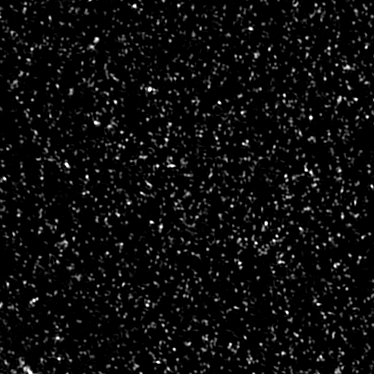

Supplement: Figure 4—source data 1. [file elife-56679-fig4-data1.zip › Figure4 - Source Data1/GFP-PSEN1 ADAM10/rois/33_PS-1.tif]

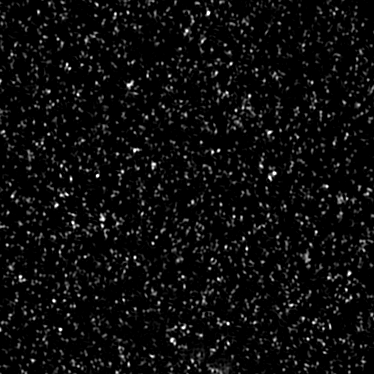

Supplement: Figure 4—source data 1. [file elife-56679-fig4-data1.zip › Figure4 - Source Data1/GFP-PSEN1 ADAM10/rois/33_PS-2.tif]

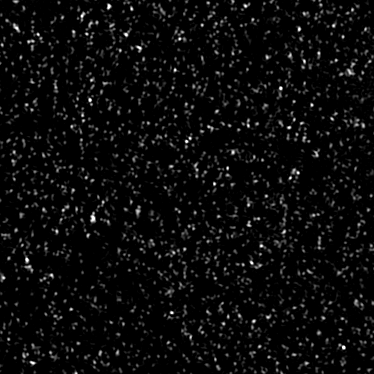

Supplement: Figure 4—source data 1. [file elife-56679-fig4-data1.zip › Figure4 - Source Data1/GFP-PSEN1 ADAM10/rois/33_PS-3.tif]

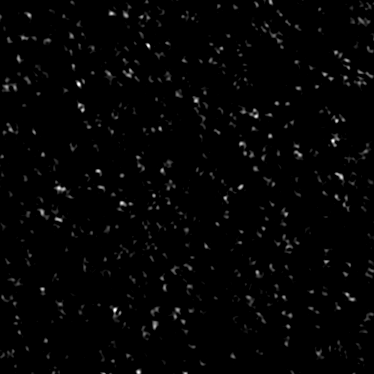

Supplement: Figure 4—source data 1. [file elife-56679-fig4-data1.zip › Figure4 - Source Data1/GFP-PSEN1 ADAM10/rois/34_A10-1.tif]

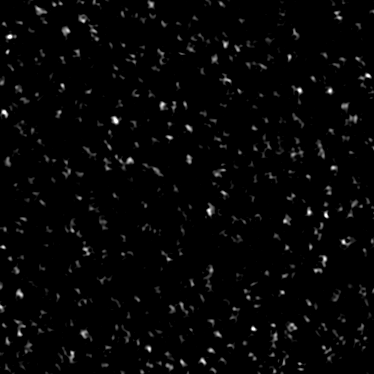

Supplement: Figure 4—source data 1. [file elife-56679-fig4-data1.zip › Figure4 - Source Data1/GFP-PSEN1 ADAM10/rois/34_A10-2.tif]
