# Supplementary material for: Super-resolution microscopy reveals majorly mono- and dimeric presenilin1/γ-secretase at the cell surface
Source: eLife. 2020 Jul 7;9:e56679. doi: 10.7554/eLife.56679 (PMC7340497; doi:10.7554/eLife.56679)
Supplement: Figure 2—source data 2. [file elife-56679-fig2-data2.zip › Figure2 - Source Data 2/Figure2-Source Data2.docx]

**Source Data for Photobleaching analysis (Figure 2)**

This .zip folder contains all widefield data used for quantification of photobleaching steps related to Figure 2-B.

The folder ROI contains processed stacks used for quantifications.

The cell line used for this experiment was TKO NCT-SNAP GFP-PSEN1 that was FACSorted for low levels of both tags as shown in Supplementary Figure1. This was to ensure minmium overlap of spots. Spots that overlap and intensity traces with no clear steps were discarded from quantififcations. Typically, around 5-8 traces from a total of ~70 traces had to be discarded.

Data recording was at 0.1s/frame. Background was substracted by ImageJ sliding paraboloid 50px rolling ball radius. Spots were defined by ImageJ Spot Intensity Analysis with a spot radous of 2px, a noise tolerance of 200 and a background estimation by Median-40.
