# Supplementary material for: Super-resolution microscopy reveals majorly mono- and dimeric presenilin1/γ-secretase at the cell surface
Source: eLife. 2020 Jul 7;9:e56679. doi: 10.7554/eLife.56679 (PMC7340497; doi:10.7554/eLife.56679)
Supplement: Figure 3—source data 1. [file elife-56679-fig3-data1.zip › Figure3 - Source Data 1/Figure3-Source Data1.docx]

**Source Data for Hotspot analysis (Figure 3)**

This .zip folder contains all SPT data used for quantification of hotspots related to Figure 3-L.

Folder names go accordingly to the treatment applied to the cells. The cell line used for this experiment was TKO NCT-SNAP GFP-PSEN1 FACSorted for high levels of both tags as shown in Supplementary Figure1. Each folder contains subfolders “DBSCAN hotspots” folder provides output images of clusters found per cell from localizations; “Tracks” folder contains SPT output of tracks as analyzed by Metamorph PALMtracer, which were overlaied on to the DBSCAN hotspot data to found how many tracks have each hotspot.
