# Supplementary material for: Super-resolution microscopy reveals majorly mono- and dimeric presenilin1/γ-secretase at the cell surface
Source: eLife. 2020 Jul 7;9:e56679. doi: 10.7554/eLife.56679 (PMC7340497; doi:10.7554/eLife.56679)
Supplement: Figure 3—source data 2. [file elife-56679-fig3-data2.zip › Figure3 - Source Data 2/Figure3-Source Data2.docx]

**Source Data for SPT analysis (Figure 3)**

This .zip folder contains all SPT data used for quantification of diffusion coefficient, Log10 D, MSD, Mobile/immobile ratio of inside/Edge, and alpha classification of Figure 3.

The Excel file has the information across different tabs, one with the summary of results, followed by statistics of the raw data per treatment. The cell line used for this experiment was TKO NCT-SNAP GFP-PSEN1 FACSorted for high levels of both tags as shown in Figure1- Figure supplemental 1.
