# Supplementary material for: Super-resolution microscopy reveals majorly mono- and dimeric presenilin1/γ-secretase at the cell surface
Source: eLife. 2020 Jul 7;9:e56679. doi: 10.7554/eLife.56679 (PMC7340497; doi:10.7554/eLife.56679)
Supplement: Figure 4—source data 1. [file elife-56679-fig4-data1.zip › Figure4 - Source Data1/Figure4-Source Data1.docx]

**Source Data for Nearest neighbor analysis (Figure 4)**

This .zip folder contains representative SIM data used for quantification of nearest neighbor distances related to Figure 4.

Each folder relates to different cell line: “GFP-PSEN1 APP” folder relates to sKO GFP-PSEN1 cell line probed with APP antibody; “GFP-PSEN1 N-cadherin” folder relates to sKO GFP-PSEN1 cell line probed with N-cadherin antibody; “GFP-PSEN1 ADAM10” folder relates to sKO GFP-PSEN1 cell line transiently expressing ADAM10-SNAP and stained with SiR-substrate; “GFP-PSEN1 BACE1” folder relates to sKO GFP-PSEN1 cell line stably expressing BACE1-SNAP FACSorted for low levels and stained with SiR-substrate.

Each folder contains subfolders showing ROIs with background substraction (done by adjusting intensity histogram) and ROIs mask (done by H-watershed, ImageJ). The final mask was used to localize spot centorids used during nearest naighbor analysis.
