# Supplementary figures and images for: Super-resolution microscopy reveals majorly mono- and dimeric presenilin1/γ-secretase at the cell surface (part 4 of 4)
Source: eLife. 2020 Jul 7;9:e56679. doi: 10.7554/eLife.56679 (PMC7340497; doi:10.7554/eLife.56679)

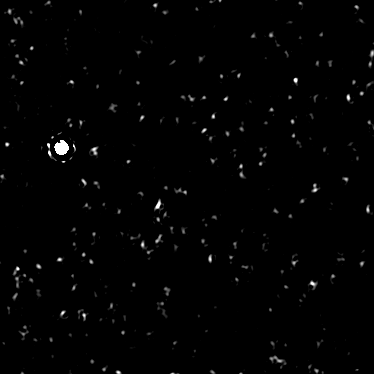

Supplement: Figure 4—source data 1. [file elife-56679-fig4-data1.zip › Figure4 - Source Data1/GFP-PSEN1 BACE1/rois/66-bace-1.tif]

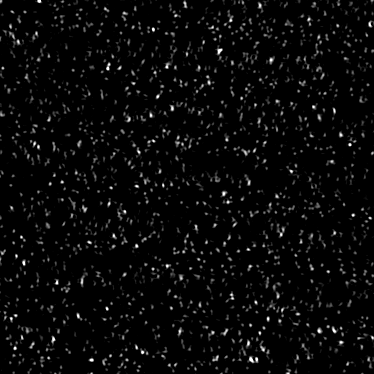

Supplement: Figure 4—source data 1. [file elife-56679-fig4-data1.zip › Figure4 - Source Data1/GFP-PSEN1 BACE1/rois/68-ps-1.tif]

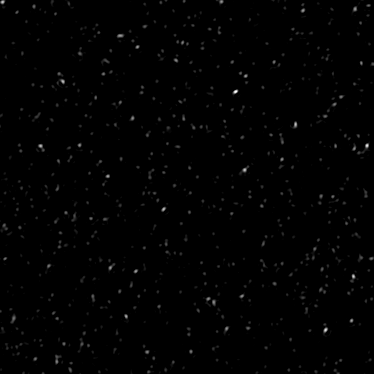

Supplement: Figure 4—source data 1. [file elife-56679-fig4-data1.zip › Figure4 - Source Data1/GFP-PSEN1 BACE1/rois/69-bace-1.tif]

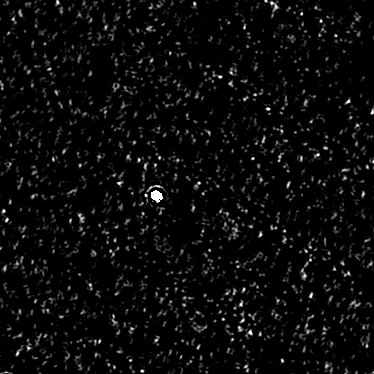

Supplement: Figure 4—source data 1. [file elife-56679-fig4-data1.zip › Figure4 - Source Data1/GFP-PSEN1 BACE1/rois/71-ps-1.tif]

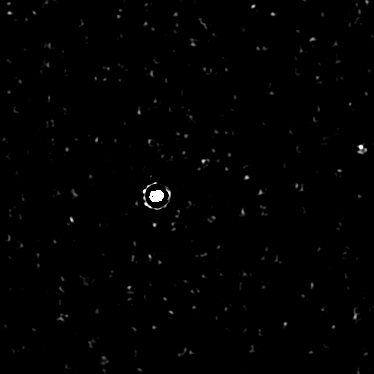

Supplement: Figure 4—source data 1. [file elife-56679-fig4-data1.zip › Figure4 - Source Data1/GFP-PSEN1 BACE1/rois/72-bace-1.tif]

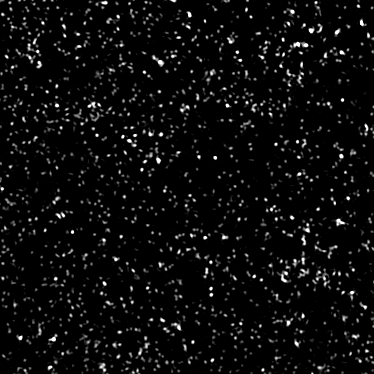

Supplement: Figure 4—source data 1. [file elife-56679-fig4-data1.zip › Figure4 - Source Data1/GFP-PSEN1 BACE1/rois/75-ps-1.tif]

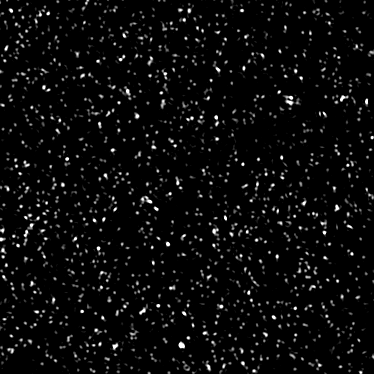

Supplement: Figure 4—source data 1. [file elife-56679-fig4-data1.zip › Figure4 - Source Data1/GFP-PSEN1 BACE1/rois/75-ps-2.tif]

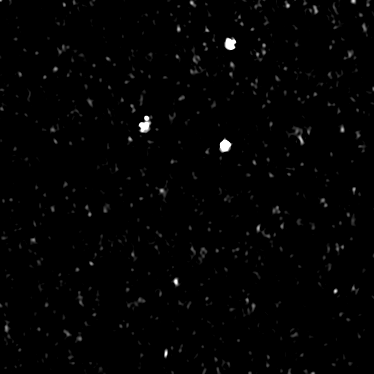

Supplement: Figure 4—source data 1. [file elife-56679-fig4-data1.zip › Figure4 - Source Data1/GFP-PSEN1 BACE1/rois/76-bace-1.tif]

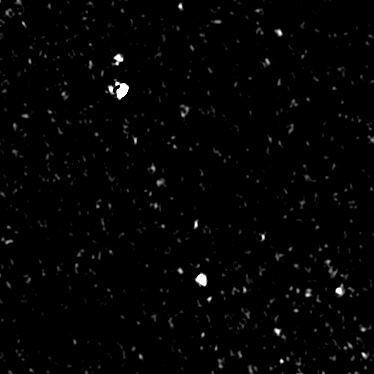

Supplement: Figure 4—source data 1. [file elife-56679-fig4-data1.zip › Figure4 - Source Data1/GFP-PSEN1 BACE1/rois/76-bace-2.tif]

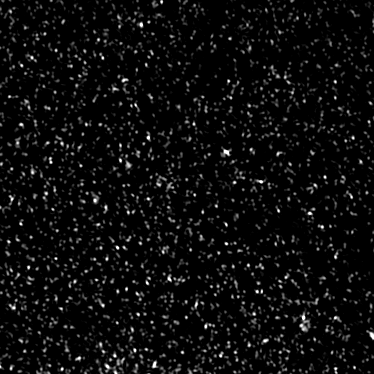

Supplement: Figure 4—source data 1. [file elife-56679-fig4-data1.zip › Figure4 - Source Data1/GFP-PSEN1 BACE1/rois/78-ps-1.tif]

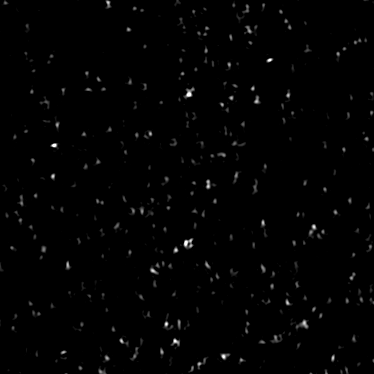

Supplement: Figure 4—source data 1. [file elife-56679-fig4-data1.zip › Figure4 - Source Data1/GFP-PSEN1 BACE1/rois/79-bace-1.tif]

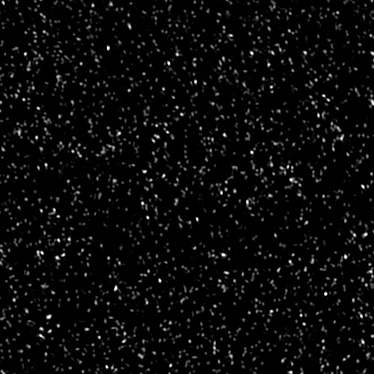

Supplement: Figure 4—source data 1. [file elife-56679-fig4-data1.zip › Figure4 - Source Data1/GFP-PSEN1 BACE1/rois/84-ps-1.tif]

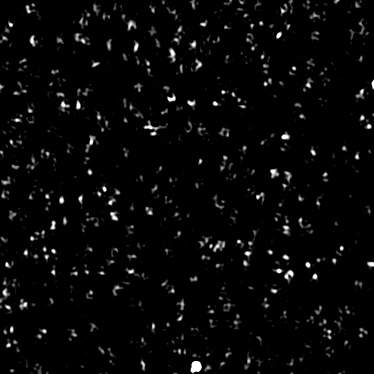

Supplement: Figure 4—source data 1. [file elife-56679-fig4-data1.zip › Figure4 - Source Data1/GFP-PSEN1 BACE1/rois/85-bace-1.tif]

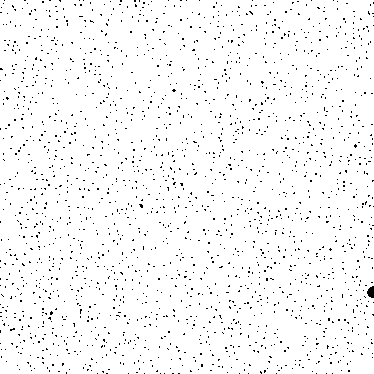

Supplement: Figure 4—source data 1. [file elife-56679-fig4-data1.zip › Figure4 - Source Data1/GFP-PSEN1 N-cadherin/roi masks/005_SIM_PS_bg-1.tif - watershed (h=1404,00, T=4213,00, %=20, n=2018).tif]

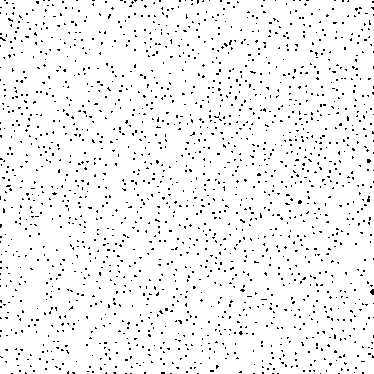

Supplement: Figure 4—source data 1. [file elife-56679-fig4-data1.zip › Figure4 - Source Data1/GFP-PSEN1 N-cadherin/roi masks/006_SIM_Ncadh_bg-1.tif - watershed (h=1404,00, T=4213,00, %=20, n=1832).tif]

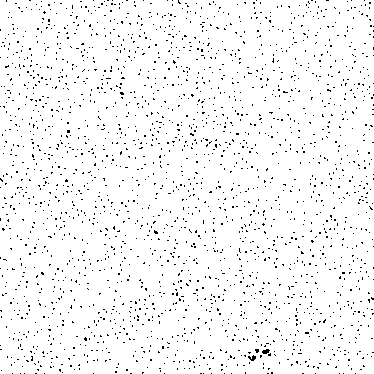

Supplement: Figure 4—source data 1. [file elife-56679-fig4-data1.zip › Figure4 - Source Data1/GFP-PSEN1 N-cadherin/roi masks/007_SIM_PS_bg-1.tif - watershed (h=1404,00, T=4213,00, %=20, n=2330).tif]

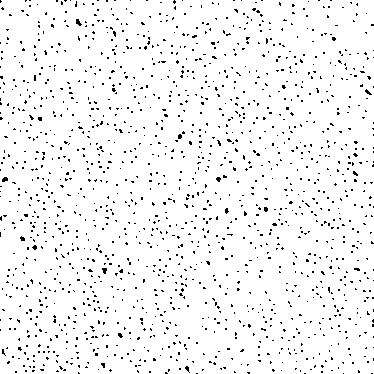

Supplement: Figure 4—source data 1. [file elife-56679-fig4-data1.zip › Figure4 - Source Data1/GFP-PSEN1 N-cadherin/roi masks/008_SIM_Ncadh_bg-1.tif - watershed (h=1404,00, T=4213,00, %=20, n=1557).tif]

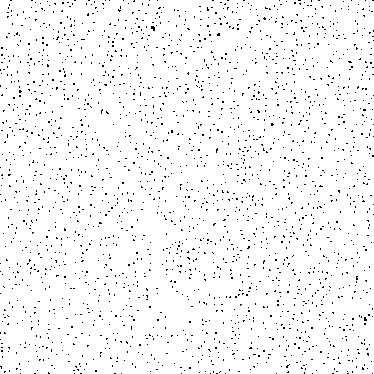

Supplement: Figure 4—source data 1. [file elife-56679-fig4-data1.zip › Figure4 - Source Data1/GFP-PSEN1 N-cadherin/roi masks/009_SIM_PS_bg-1.tif - watershed (h=1404,00, T=4213,00, %=20, n=2242).tif]

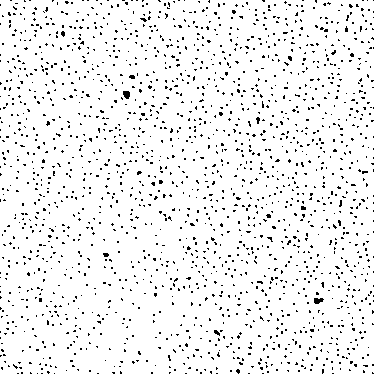

Supplement: Figure 4—source data 1. [file elife-56679-fig4-data1.zip › Figure4 - Source Data1/GFP-PSEN1 N-cadherin/roi masks/010_SIM_Ncadh_bg-1.tif - watershed (h=1404,00, T=4213,00, %=20, n=1904).tif]

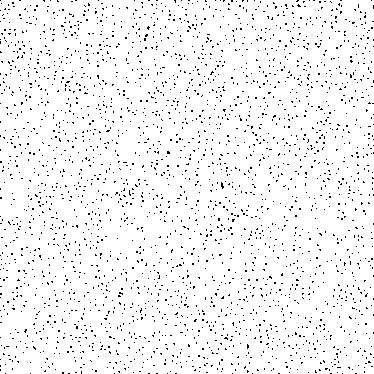

Supplement: Figure 4—source data 1. [file elife-56679-fig4-data1.zip › Figure4 - Source Data1/GFP-PSEN1 N-cadherin/roi masks/011_SIM_PS_bg-1.tif - watershed (h=1404,00, T=4213,00, %=20, n=2715).tif]

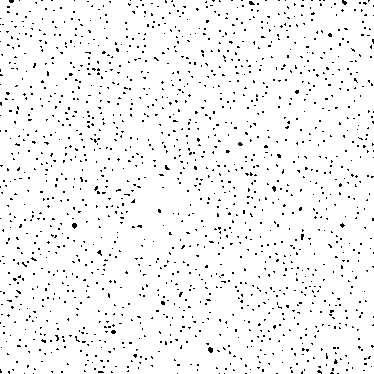

Supplement: Figure 4—source data 1. [file elife-56679-fig4-data1.zip › Figure4 - Source Data1/GFP-PSEN1 N-cadherin/roi masks/012_SIM_Ncadh_bg-1.tif - watershed (h=1404,00, T=4213,00, %=20, n=1573).tif]

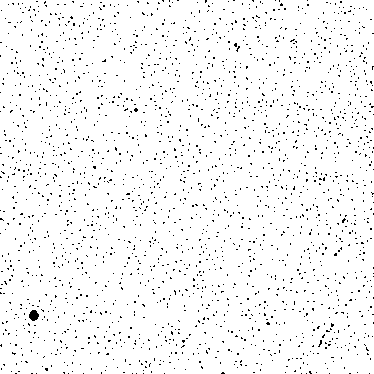

Supplement: Figure 4—source data 1. [file elife-56679-fig4-data1.zip › Figure4 - Source Data1/GFP-PSEN1 N-cadherin/roi masks/013_SIM_PS_bg-1.tif - watershed (h=1404,00, T=4213,00, %=20, n=2203).tif]

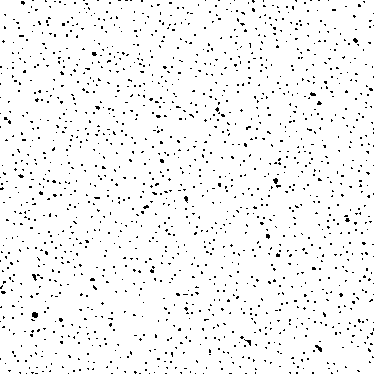

Supplement: Figure 4—source data 1. [file elife-56679-fig4-data1.zip › Figure4 - Source Data1/GFP-PSEN1 N-cadherin/roi masks/014_SIM_Ncadh_bg-1.tif - watershed (h=1404,00, T=4213,00, %=20, n=1542).tif]

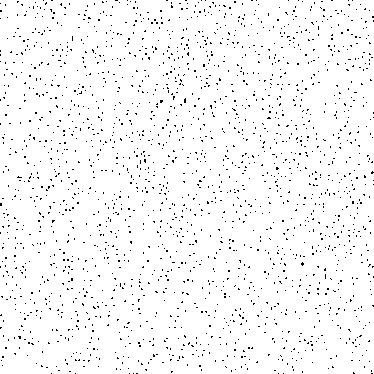

Supplement: Figure 4—source data 1. [file elife-56679-fig4-data1.zip › Figure4 - Source Data1/GFP-PSEN1 N-cadherin/roi masks/015_SIM_PS-1.tif - watershed (h=1404,00, T=4213,00, %=20, n=2077).tif]

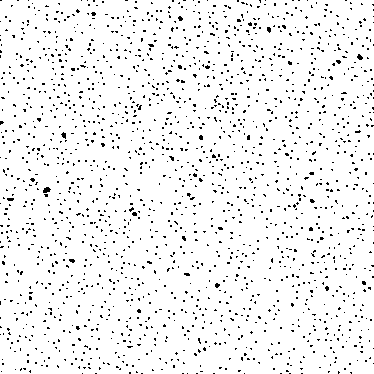

Supplement: Figure 4—source data 1. [file elife-56679-fig4-data1.zip › Figure4 - Source Data1/GFP-PSEN1 N-cadherin/roi masks/016_SIM_Ncadh_bg-1.tif - watershed (h=1404,00, T=4213,00, %=20, n=1815).tif]

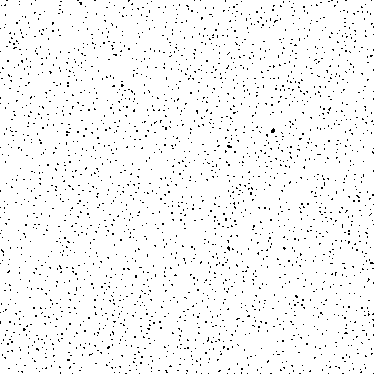

Supplement: Figure 4—source data 1. [file elife-56679-fig4-data1.zip › Figure4 - Source Data1/GFP-PSEN1 N-cadherin/roi masks/017_SIM_PS_bg-1.tif - watershed (h=1404,00, T=4213,00, %=20, n=2388).tif]

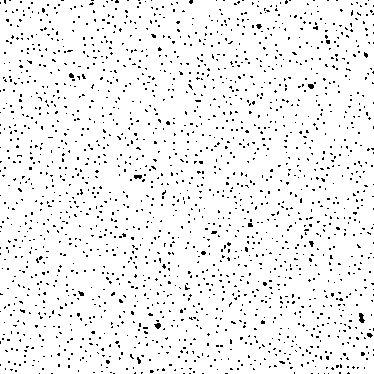

Supplement: Figure 4—source data 1. [file elife-56679-fig4-data1.zip › Figure4 - Source Data1/GFP-PSEN1 N-cadherin/roi masks/018_SIM_Ncadh_bg-1.tif - watershed (h=1404,00, T=4213,00, %=20, n=1931).tif]

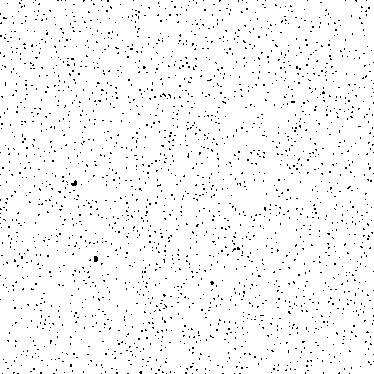

Supplement: Figure 4—source data 1. [file elife-56679-fig4-data1.zip › Figure4 - Source Data1/GFP-PSEN1 N-cadherin/roi masks/019_SIM_PS_bg-1.tif - watershed (h=1404,00, T=4213,00, %=20, n=2232).tif]

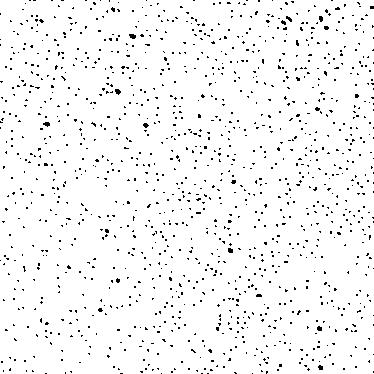

Supplement: Figure 4—source data 1. [file elife-56679-fig4-data1.zip › Figure4 - Source Data1/GFP-PSEN1 N-cadherin/roi masks/020_SIM_Ncadh_bg-1.tif - watershed (h=1404,00, T=4213,00, %=20, n=1305).tif]

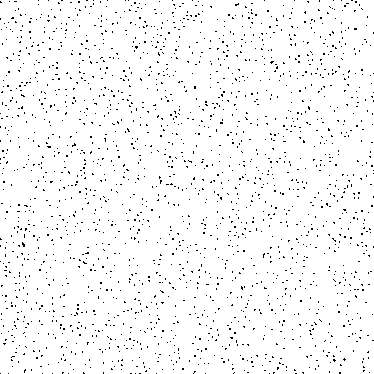

Supplement: Figure 4—source data 1. [file elife-56679-fig4-data1.zip › Figure4 - Source Data1/GFP-PSEN1 N-cadherin/roi masks/021_SIM_PS_bg-1.tif - watershed (h=1404,00, T=4213,00, %=20, n=2070).tif]

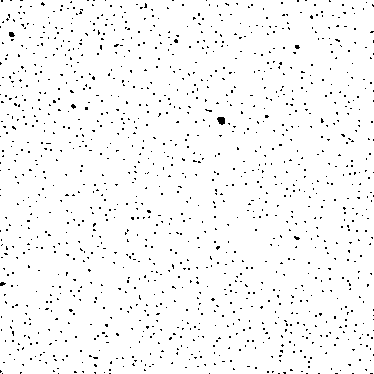

Supplement: Figure 4—source data 1. [file elife-56679-fig4-data1.zip › Figure4 - Source Data1/GFP-PSEN1 N-cadherin/roi masks/022_SIM_Ncadh_bg-1.tif - watershed (h=1404,00, T=4213,00, %=20, n=1280).tif]

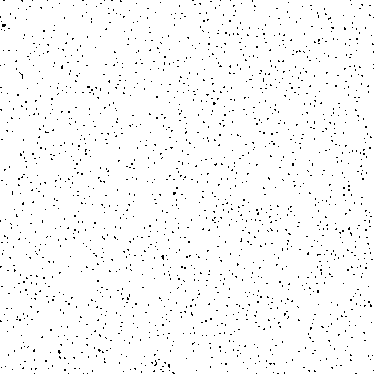

Supplement: Figure 4—source data 1. [file elife-56679-fig4-data1.zip › Figure4 - Source Data1/GFP-PSEN1 N-cadherin/roi masks/023_SIM_PS_bg-1.tif - watershed (h=1404,00, T=4213,00, %=20, n=1704).tif]

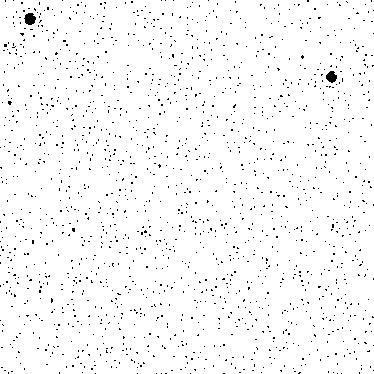

Supplement: Figure 4—source data 1. [file elife-56679-fig4-data1.zip › Figure4 - Source Data1/GFP-PSEN1 N-cadherin/roi masks/023_SIM_PS_bg-2.tif - watershed (h=1404,00, T=4213,00, %=20, n=1640).tif]

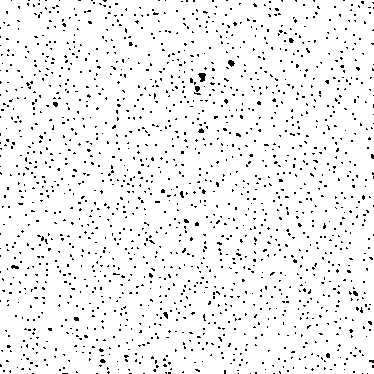

Supplement: Figure 4—source data 1. [file elife-56679-fig4-data1.zip › Figure4 - Source Data1/GFP-PSEN1 N-cadherin/roi masks/024_SIM_Ncadh_bg-1.tif - watershed (h=1404,00, T=4213,00, %=20, n=1710).tif]

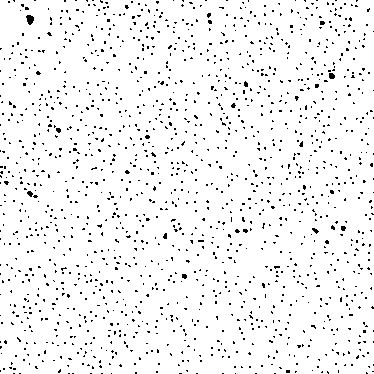

Supplement: Figure 4—source data 1. [file elife-56679-fig4-data1.zip › Figure4 - Source Data1/GFP-PSEN1 N-cadherin/roi masks/024_SIM_Ncadh_bg-2.tif - watershed (h=1404,00, T=4213,00, %=20, n=1539).tif]

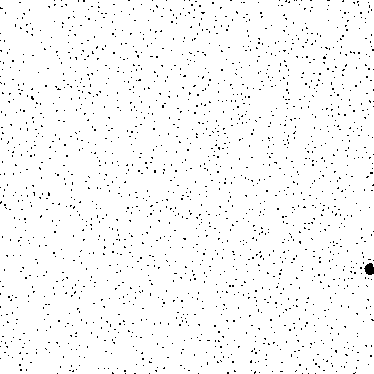

Supplement: Figure 4—source data 1. [file elife-56679-fig4-data1.zip › Figure4 - Source Data1/GFP-PSEN1 N-cadherin/roi masks/025_SIM_PS_bg-1.tif - watershed (h=1404,00, T=4213,00, %=20, n=1841).tif]

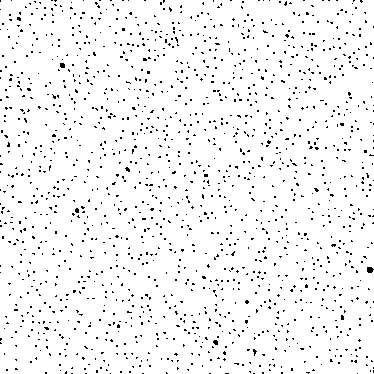

Supplement: Figure 4—source data 1. [file elife-56679-fig4-data1.zip › Figure4 - Source Data1/GFP-PSEN1 N-cadherin/roi masks/026_SIM_Ncadh_bg-1.tif - watershed (h=1404,00, T=4213,00, %=20, n=1577).tif]

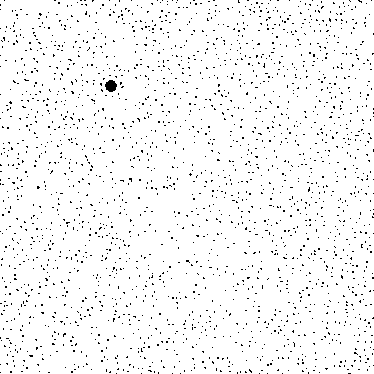

Supplement: Figure 4—source data 1. [file elife-56679-fig4-data1.zip › Figure4 - Source Data1/GFP-PSEN1 N-cadherin/roi masks/027_SIM_PS_bg-1.tif - watershed (h=1404,00, T=4213,00, %=20, n=2226).tif]

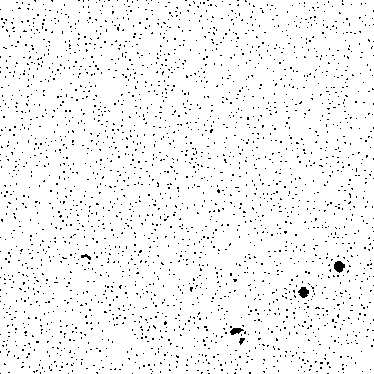

Supplement: Figure 4—source data 1. [file elife-56679-fig4-data1.zip › Figure4 - Source Data1/GFP-PSEN1 N-cadherin/roi masks/027_SIM_PS_bg-2.tif - watershed (h=1404,00, T=4213,00, %=20, n=2584).tif]

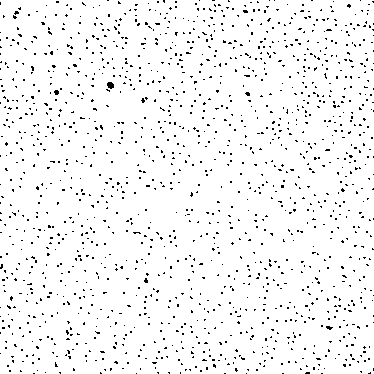

Supplement: Figure 4—source data 1. [file elife-56679-fig4-data1.zip › Figure4 - Source Data1/GFP-PSEN1 N-cadherin/roi masks/028_SIM_Ncadh_bg-1.tif - watershed (h=1404,00, T=4213,00, %=20, n=1529).tif]

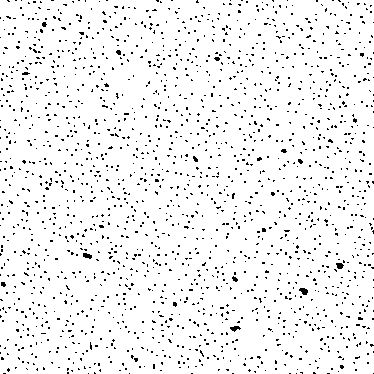

Supplement: Figure 4—source data 1. [file elife-56679-fig4-data1.zip › Figure4 - Source Data1/GFP-PSEN1 N-cadherin/roi masks/028_SIM_Ncadh_bg-2.tif - watershed (h=1404,00, T=4213,00, %=20, n=1840).tif]

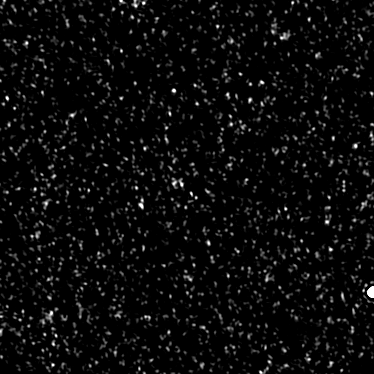

Supplement: Figure 4—source data 1. [file elife-56679-fig4-data1.zip › Figure4 - Source Data1/GFP-PSEN1 N-cadherin/rois/005_SIM_PS_bg-1.tif]

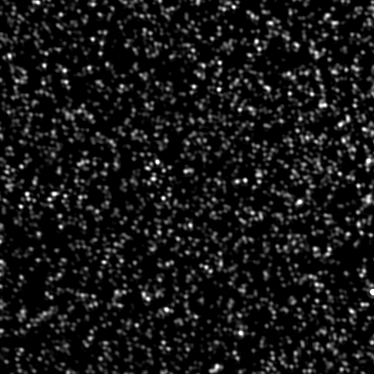

Supplement: Figure 4—source data 1. [file elife-56679-fig4-data1.zip › Figure4 - Source Data1/GFP-PSEN1 N-cadherin/rois/006_SIM_Ncadh_bg-1.tif]

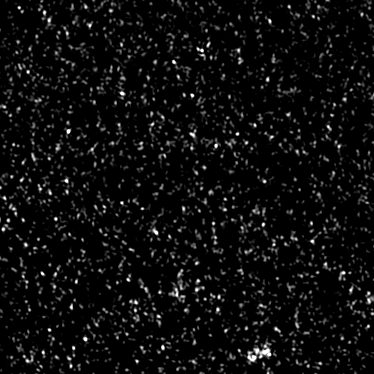

Supplement: Figure 4—source data 1. [file elife-56679-fig4-data1.zip › Figure4 - Source Data1/GFP-PSEN1 N-cadherin/rois/007_SIM_PS_bg-1.tif]

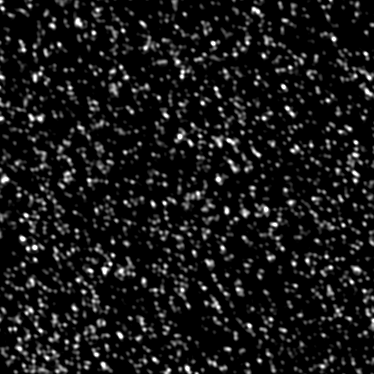

Supplement: Figure 4—source data 1. [file elife-56679-fig4-data1.zip › Figure4 - Source Data1/GFP-PSEN1 N-cadherin/rois/008_SIM_Ncadh_bg-1.tif]

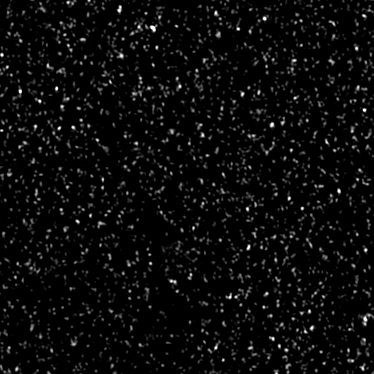

Supplement: Figure 4—source data 1. [file elife-56679-fig4-data1.zip › Figure4 - Source Data1/GFP-PSEN1 N-cadherin/rois/009_SIM_PS_bg-1.tif]

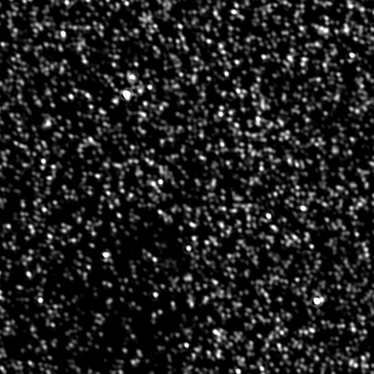

Supplement: Figure 4—source data 1. [file elife-56679-fig4-data1.zip › Figure4 - Source Data1/GFP-PSEN1 N-cadherin/rois/010_SIM_Ncadh_bg-1.tif]

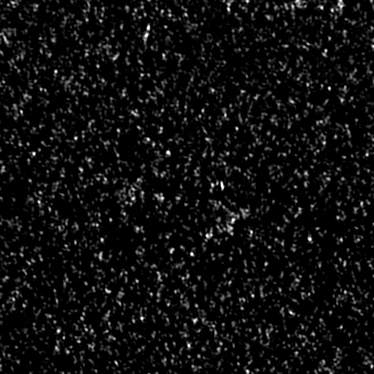

Supplement: Figure 4—source data 1. [file elife-56679-fig4-data1.zip › Figure4 - Source Data1/GFP-PSEN1 N-cadherin/rois/011_SIM_PS_bg-1.tif]
