# Supplementary figures and images for: Super-resolution microscopy reveals majorly mono- and dimeric presenilin1/γ-secretase at the cell surface (part 3 of 4)
Source: eLife. 2020 Jul 7;9:e56679. doi: 10.7554/eLife.56679 (PMC7340497; doi:10.7554/eLife.56679)

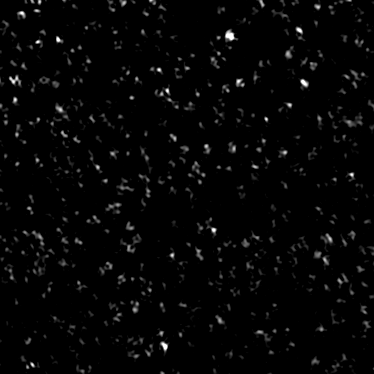

Supplement: Figure 4—source data 1. [file elife-56679-fig4-data1.zip › Figure4 - Source Data1/GFP-PSEN1 ADAM10/rois/34_A10-3.tif]

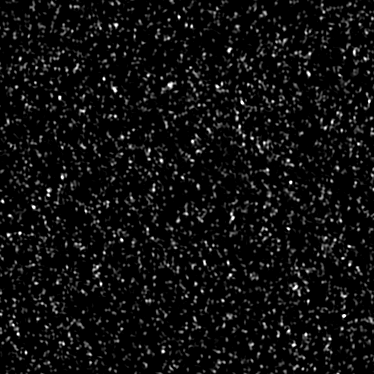

Supplement: Figure 4—source data 1. [file elife-56679-fig4-data1.zip › Figure4 - Source Data1/GFP-PSEN1 ADAM10/rois/35_PS-1.tif]

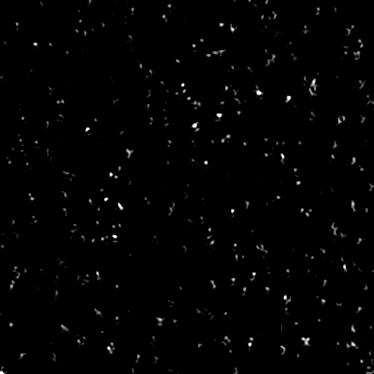

Supplement: Figure 4—source data 1. [file elife-56679-fig4-data1.zip › Figure4 - Source Data1/GFP-PSEN1 ADAM10/rois/36_A10-1.tif]

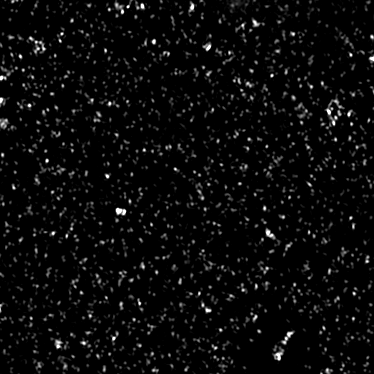

Supplement: Figure 4—source data 1. [file elife-56679-fig4-data1.zip › Figure4 - Source Data1/GFP-PSEN1 ADAM10/rois/37_PS-1.tif]

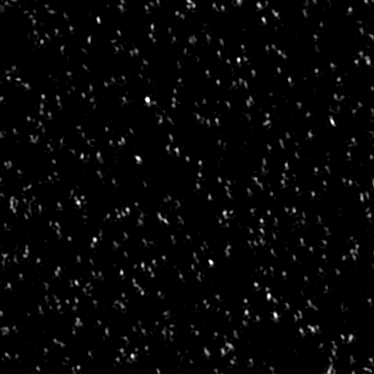

Supplement: Figure 4—source data 1. [file elife-56679-fig4-data1.zip › Figure4 - Source Data1/GFP-PSEN1 ADAM10/rois/38_A10-1.tif]

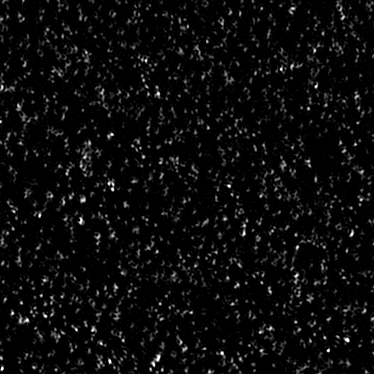

Supplement: Figure 4—source data 1. [file elife-56679-fig4-data1.zip › Figure4 - Source Data1/GFP-PSEN1 ADAM10/rois/39_PS-1.tif]

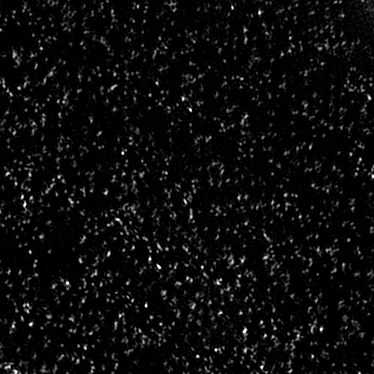

Supplement: Figure 4—source data 1. [file elife-56679-fig4-data1.zip › Figure4 - Source Data1/GFP-PSEN1 ADAM10/rois/39_PS-2.tif]

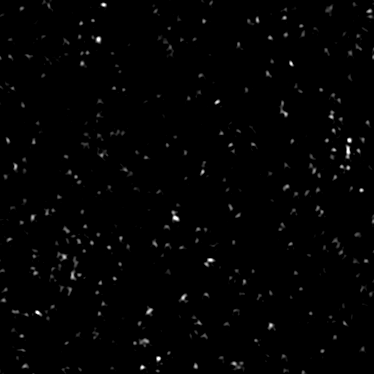

Supplement: Figure 4—source data 1. [file elife-56679-fig4-data1.zip › Figure4 - Source Data1/GFP-PSEN1 ADAM10/rois/40_A10-1.tif]

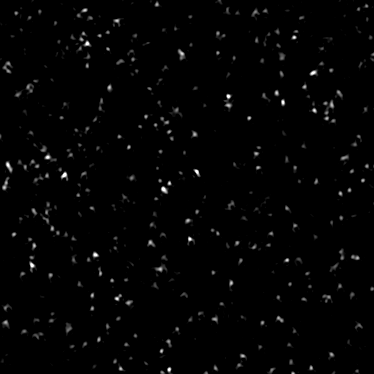

Supplement: Figure 4—source data 1. [file elife-56679-fig4-data1.zip › Figure4 - Source Data1/GFP-PSEN1 ADAM10/rois/40_A10-2.tif]

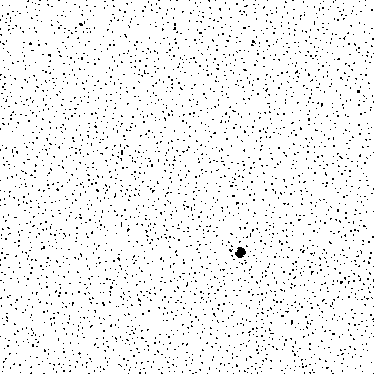

Supplement: Figure 4—source data 1. [file elife-56679-fig4-data1.zip › Figure4 - Source Data1/GFP-PSEN1 APP/roi masks/001_PS_SIM_Image 1-1.tif - watershed (h=1404,00, T=4213,00, %=20, n=2989).tif]

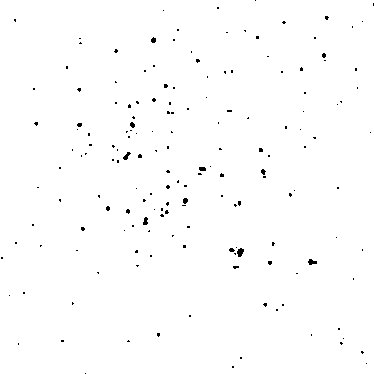

Supplement: Figure 4—source data 1. [file elife-56679-fig4-data1.zip › Figure4 - Source Data1/GFP-PSEN1 APP/roi masks/002_APP_SIM_Image 2-1.tif - watershed (h= 0,00, T=20000,00, %=50, n=168).tif]

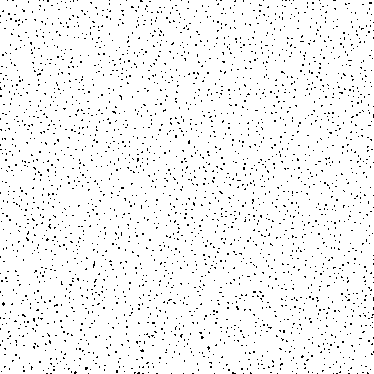

Supplement: Figure 4—source data 1. [file elife-56679-fig4-data1.zip › Figure4 - Source Data1/GFP-PSEN1 APP/roi masks/003_PS_SIM_Image 3-1.tif - watershed (h=1404,00, T=4213,00, %=20, n=2734).tif]

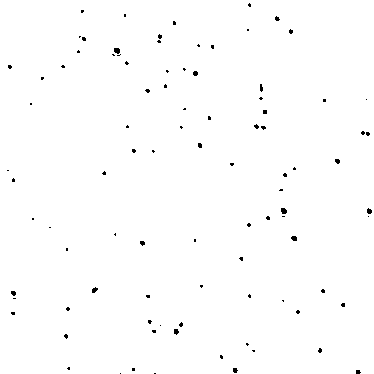

Supplement: Figure 4—source data 1. [file elife-56679-fig4-data1.zip › Figure4 - Source Data1/GFP-PSEN1 APP/roi masks/004_APP_SIM_Image 4-1.tif - watershed (h= 0,00, T=20000,00, %=50, n=93).tif]

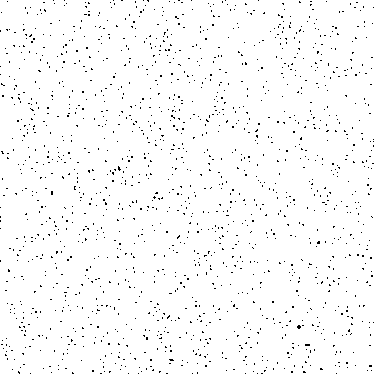

Supplement: Figure 4—source data 1. [file elife-56679-fig4-data1.zip › Figure4 - Source Data1/GFP-PSEN1 APP/roi masks/005_PS_SIM_Image 5-1.tif - watershed (h=1404,00, T=4213,00, %=20, n=1556).tif]

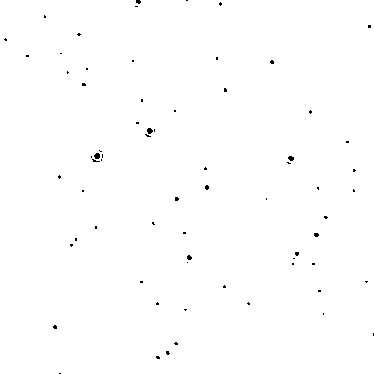

Supplement: Figure 4—source data 1. [file elife-56679-fig4-data1.zip › Figure4 - Source Data1/GFP-PSEN1 APP/roi masks/006_APP_SIM_Image 6-1.tif - watershed (h= 0,00, T=20000,00, %=50, n=69).tif]

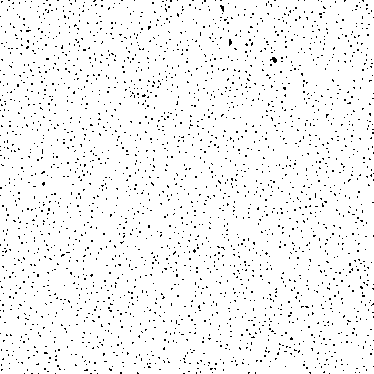

Supplement: Figure 4—source data 1. [file elife-56679-fig4-data1.zip › Figure4 - Source Data1/GFP-PSEN1 APP/roi masks/007_PS_SIM_Image 7-1.tif - watershed (h=1404,00, T=4213,00, %=20, n=2510).tif]

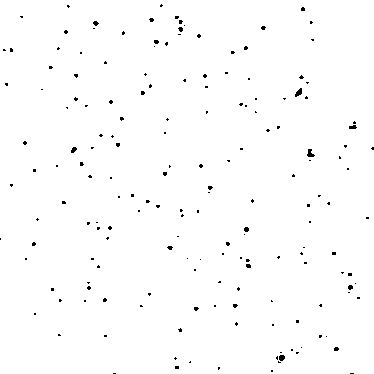

Supplement: Figure 4—source data 1. [file elife-56679-fig4-data1.zip › Figure4 - Source Data1/GFP-PSEN1 APP/roi masks/008_APP_SIM_Image 8-1.tif - watershed (h= 0,00, T=20000,00, %=50, n=175).tif]

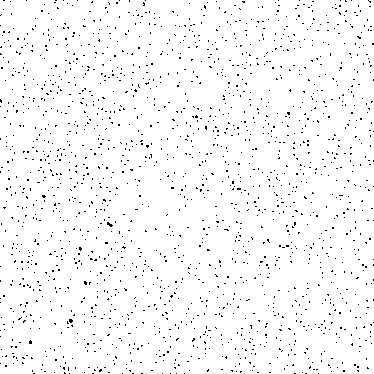

Supplement: Figure 4—source data 1. [file elife-56679-fig4-data1.zip › Figure4 - Source Data1/GFP-PSEN1 APP/roi masks/009_PS_SIM_Image 9-1.tif - watershed (h=1404,00, T=4213,00, %=20, n=1835).tif]

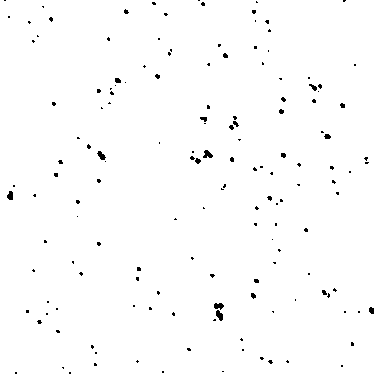

Supplement: Figure 4—source data 1. [file elife-56679-fig4-data1.zip › Figure4 - Source Data1/GFP-PSEN1 APP/roi masks/010_APP_SIM_Image 10-1.tif - watershed (h= 0,00, T=20000,00, %=50, n=152).tif]

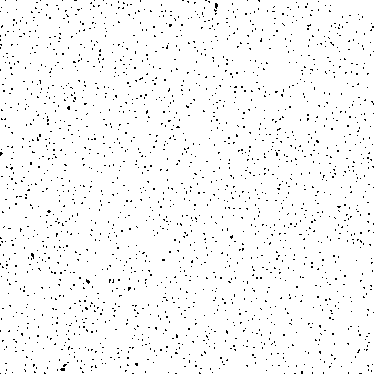

Supplement: Figure 4—source data 1. [file elife-56679-fig4-data1.zip › Figure4 - Source Data1/GFP-PSEN1 APP/roi masks/011_PS_SIM_Image 11-1.tif - watershed (h=1404,00, T=4213,00, %=20, n=1985).tif]

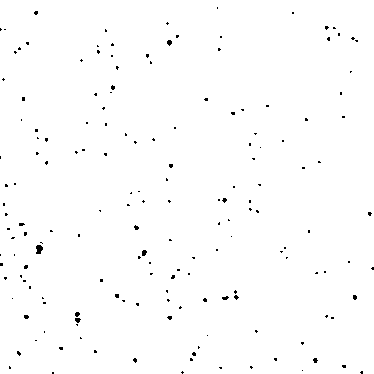

Supplement: Figure 4—source data 1. [file elife-56679-fig4-data1.zip › Figure4 - Source Data1/GFP-PSEN1 APP/roi masks/012_APP_SIM_Image 12-1.tif - watershed (h= 0,00, T=20000,00, %=50, n=168).tif]

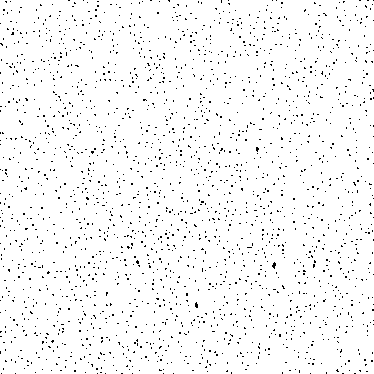

Supplement: Figure 4—source data 1. [file elife-56679-fig4-data1.zip › Figure4 - Source Data1/GFP-PSEN1 APP/roi masks/015_PS_SIM_Image 15-1.tif - watershed (h=1404,00, T=4213,00, %=20, n=2131).tif]

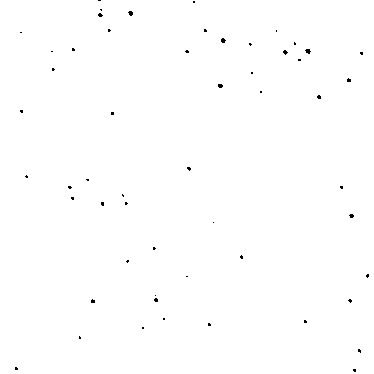

Supplement: Figure 4—source data 1. [file elife-56679-fig4-data1.zip › Figure4 - Source Data1/GFP-PSEN1 APP/roi masks/016_APP_SIM_Image 16-1.tif - watershed (h= 0,00, T=20000,00, %=50, n=55).tif]

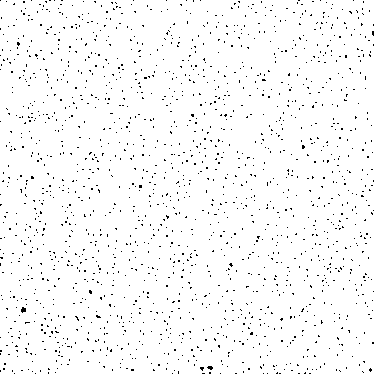

Supplement: Figure 4—source data 1. [file elife-56679-fig4-data1.zip › Figure4 - Source Data1/GFP-PSEN1 APP/roi masks/020_PS_SIM_Image 20-1.tif - watershed (h=1404,00, T=4213,00, %=20, n=1734).tif]

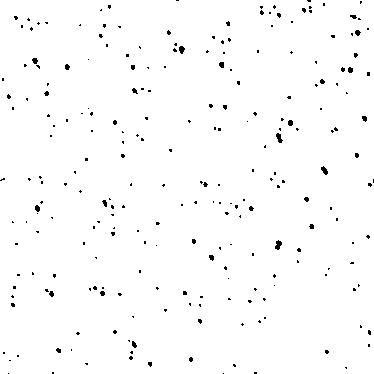

Supplement: Figure 4—source data 1. [file elife-56679-fig4-data1.zip › Figure4 - Source Data1/GFP-PSEN1 APP/roi masks/021_APP_SIM_Image 21-1.tif - watershed (h= 0,00, T=20000,00, %=50, n=241).tif]

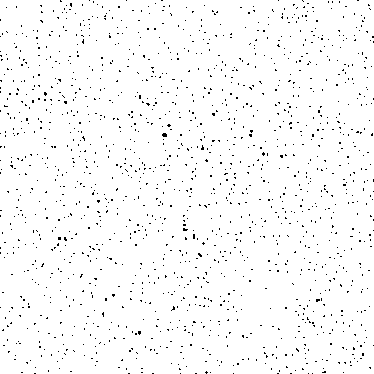

Supplement: Figure 4—source data 1. [file elife-56679-fig4-data1.zip › Figure4 - Source Data1/GFP-PSEN1 APP/roi masks/022_PS_SIM_Image 22-1.tif - watershed (h=1404,00, T=4213,00, %=20, n=1525).tif]

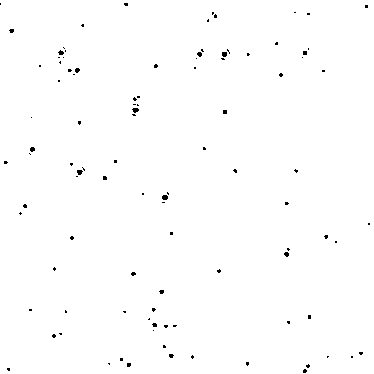

Supplement: Figure 4—source data 1. [file elife-56679-fig4-data1.zip › Figure4 - Source Data1/GFP-PSEN1 APP/roi masks/023_APP_SIM_Image 23-1.tif - watershed (h= 0,00, T=20000,00, %=50, n=101).tif]

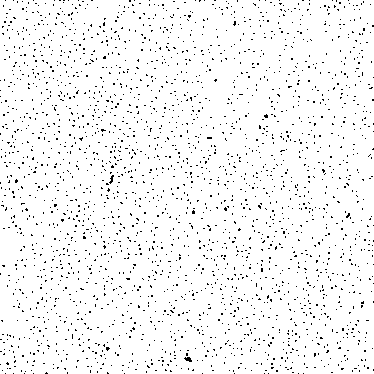

Supplement: Figure 4—source data 1. [file elife-56679-fig4-data1.zip › Figure4 - Source Data1/GFP-PSEN1 APP/roi masks/026_PS_SIM_Image 26-1.tif - watershed (h=1404,00, T=4213,00, %=20, n=2527).tif]

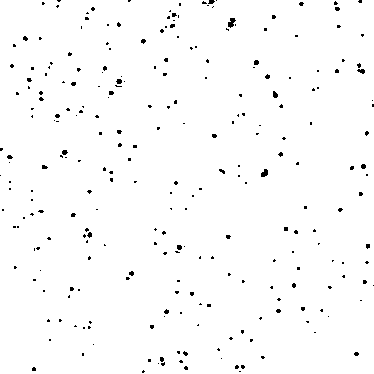

Supplement: Figure 4—source data 1. [file elife-56679-fig4-data1.zip › Figure4 - Source Data1/GFP-PSEN1 APP/roi masks/027_APP_SIM_Image 27-1.tif - watershed (h= 0,00, T=20000,00, %=50, n=258).tif]

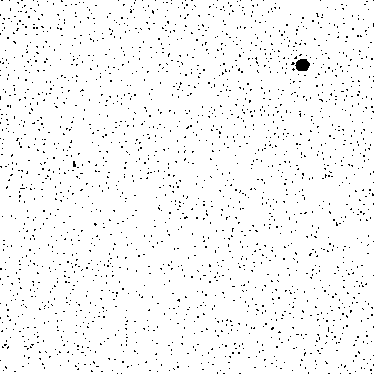

Supplement: Figure 4—source data 1. [file elife-56679-fig4-data1.zip › Figure4 - Source Data1/GFP-PSEN1 APP/roi masks/028_PS_SIM_Image 28-1.tif - watershed (h=1404,00, T=4213,00, %=20, n=2088).tif]

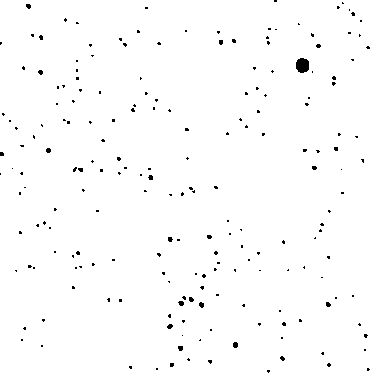

Supplement: Figure 4—source data 1. [file elife-56679-fig4-data1.zip › Figure4 - Source Data1/GFP-PSEN1 APP/roi masks/029_APP_SIM_Image 29-1.tif - watershed (h= 0,00, T=20000,00, %=50, n=201).tif]

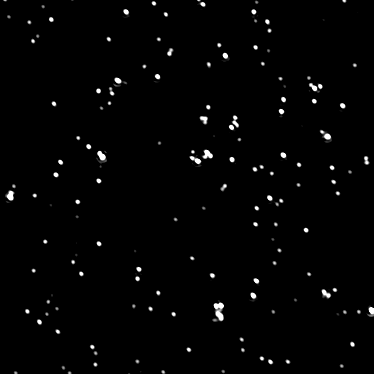

Supplement: Figure 4—source data 1. [file elife-56679-fig4-data1.zip › Figure4 - Source Data1/GFP-PSEN1 APP/rois/APP_SIM_Image 10-1.tif]

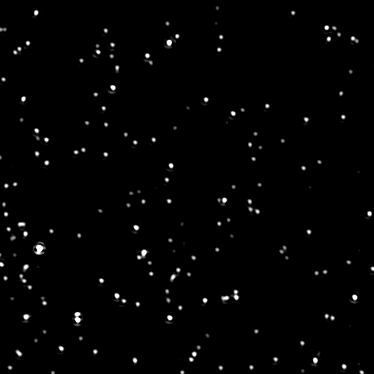

Supplement: Figure 4—source data 1. [file elife-56679-fig4-data1.zip › Figure4 - Source Data1/GFP-PSEN1 APP/rois/APP_SIM_Image 12-1.tif]

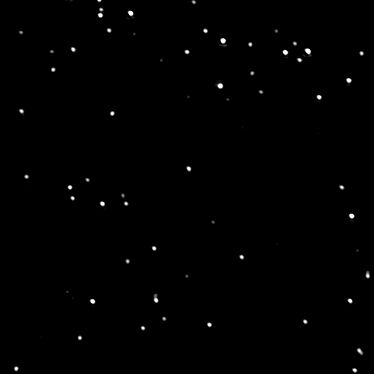

Supplement: Figure 4—source data 1. [file elife-56679-fig4-data1.zip › Figure4 - Source Data1/GFP-PSEN1 APP/rois/APP_SIM_Image 16-1.tif]

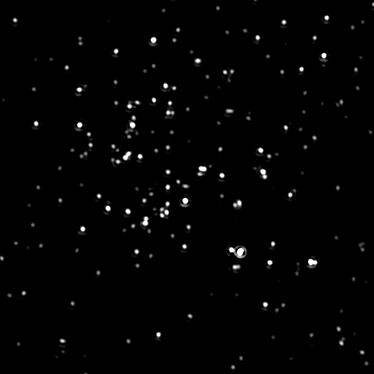

Supplement: Figure 4—source data 1. [file elife-56679-fig4-data1.zip › Figure4 - Source Data1/GFP-PSEN1 APP/rois/APP_SIM_Image 2-1.tif]

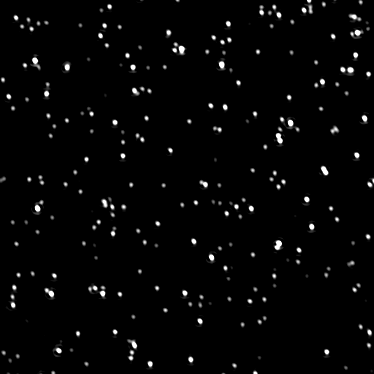

Supplement: Figure 4—source data 1. [file elife-56679-fig4-data1.zip › Figure4 - Source Data1/GFP-PSEN1 APP/rois/APP_SIM_Image 21-1.tif]

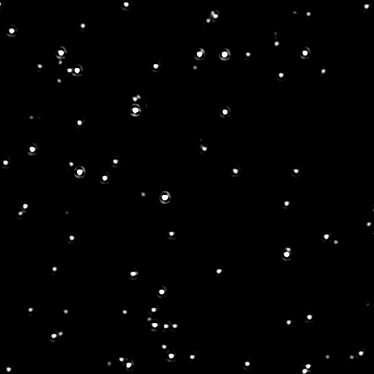

Supplement: Figure 4—source data 1. [file elife-56679-fig4-data1.zip › Figure4 - Source Data1/GFP-PSEN1 APP/rois/APP_SIM_Image 23-1.tif]

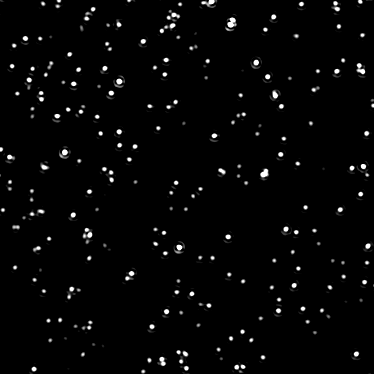

Supplement: Figure 4—source data 1. [file elife-56679-fig4-data1.zip › Figure4 - Source Data1/GFP-PSEN1 APP/rois/APP_SIM_Image 27-1.tif]

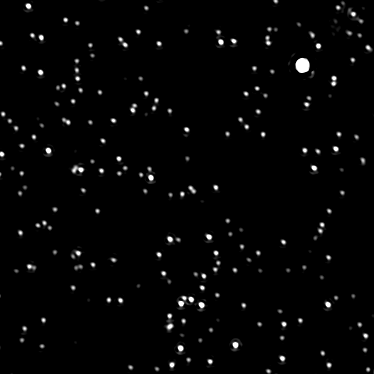

Supplement: Figure 4—source data 1. [file elife-56679-fig4-data1.zip › Figure4 - Source Data1/GFP-PSEN1 APP/rois/APP_SIM_Image 29-1.tif]

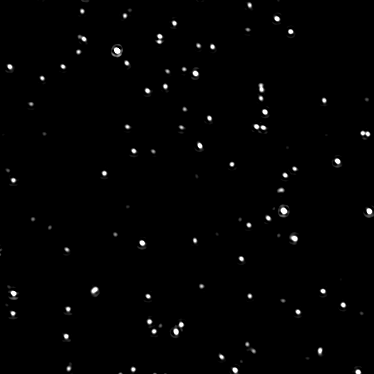

Supplement: Figure 4—source data 1. [file elife-56679-fig4-data1.zip › Figure4 - Source Data1/GFP-PSEN1 APP/rois/APP_SIM_Image 4-1.tif]

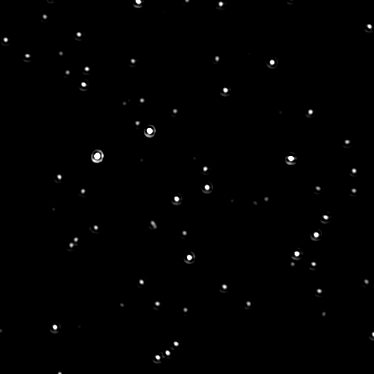

Supplement: Figure 4—source data 1. [file elife-56679-fig4-data1.zip › Figure4 - Source Data1/GFP-PSEN1 APP/rois/APP_SIM_Image 6-1.tif]

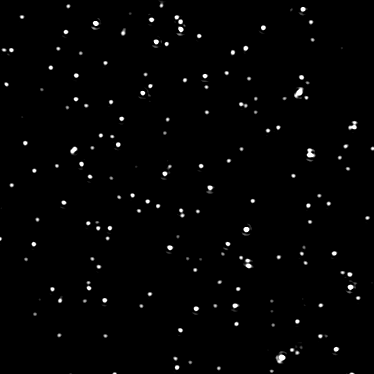

Supplement: Figure 4—source data 1. [file elife-56679-fig4-data1.zip › Figure4 - Source Data1/GFP-PSEN1 APP/rois/APP_SIM_Image 8-1.tif]

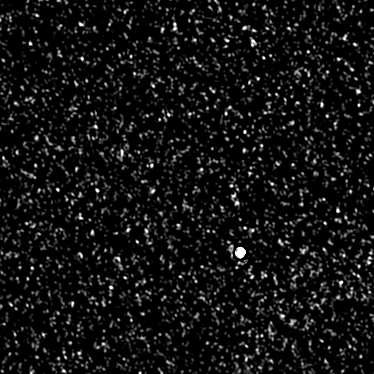

Supplement: Figure 4—source data 1. [file elife-56679-fig4-data1.zip › Figure4 - Source Data1/GFP-PSEN1 APP/rois/PS_SIM_Image 1-1.tif]

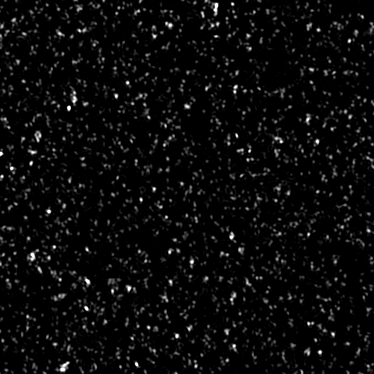

Supplement: Figure 4—source data 1. [file elife-56679-fig4-data1.zip › Figure4 - Source Data1/GFP-PSEN1 APP/rois/PS_SIM_Image 11-1.tif]

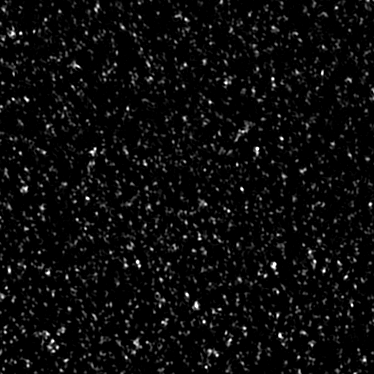

Supplement: Figure 4—source data 1. [file elife-56679-fig4-data1.zip › Figure4 - Source Data1/GFP-PSEN1 APP/rois/PS_SIM_Image 15-1.tif]

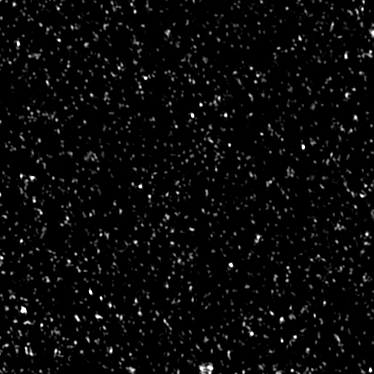

Supplement: Figure 4—source data 1. [file elife-56679-fig4-data1.zip › Figure4 - Source Data1/GFP-PSEN1 APP/rois/PS_SIM_Image 20-1.tif]

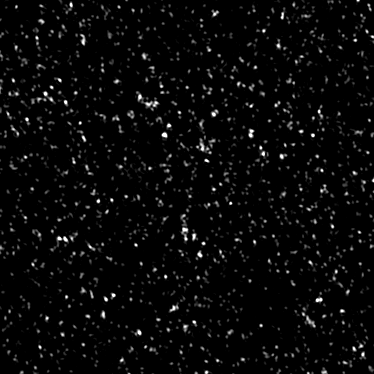

Supplement: Figure 4—source data 1. [file elife-56679-fig4-data1.zip › Figure4 - Source Data1/GFP-PSEN1 APP/rois/PS_SIM_Image 22-1.tif]

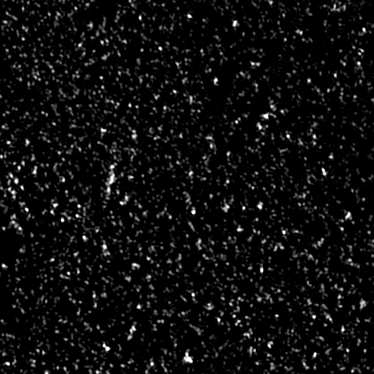

Supplement: Figure 4—source data 1. [file elife-56679-fig4-data1.zip › Figure4 - Source Data1/GFP-PSEN1 APP/rois/PS_SIM_Image 26-1.tif]

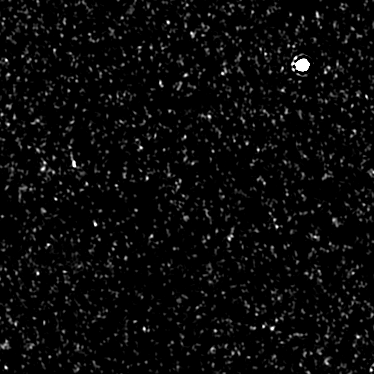

Supplement: Figure 4—source data 1. [file elife-56679-fig4-data1.zip › Figure4 - Source Data1/GFP-PSEN1 APP/rois/PS_SIM_Image 28-1.tif]

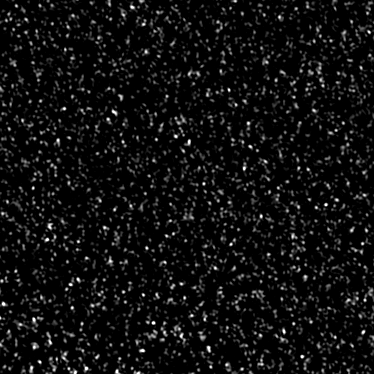

Supplement: Figure 4—source data 1. [file elife-56679-fig4-data1.zip › Figure4 - Source Data1/GFP-PSEN1 APP/rois/PS_SIM_Image 3-1.tif]

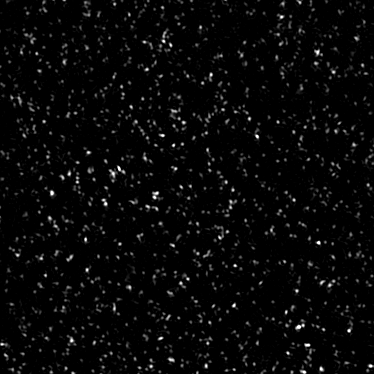

Supplement: Figure 4—source data 1. [file elife-56679-fig4-data1.zip › Figure4 - Source Data1/GFP-PSEN1 APP/rois/PS_SIM_Image 5-1.tif]

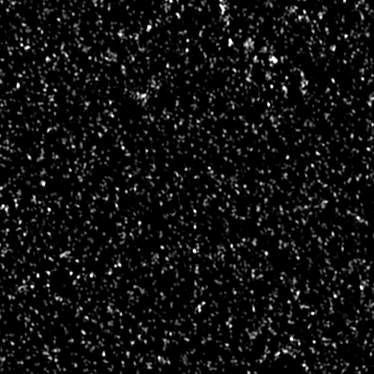

Supplement: Figure 4—source data 1. [file elife-56679-fig4-data1.zip › Figure4 - Source Data1/GFP-PSEN1 APP/rois/PS_SIM_Image 7-1.tif]

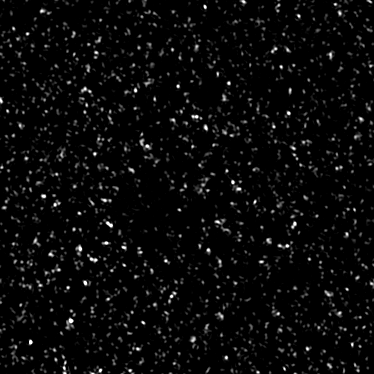

Supplement: Figure 4—source data 1. [file elife-56679-fig4-data1.zip › Figure4 - Source Data1/GFP-PSEN1 APP/rois/PS_SIM_Image 9-1.tif]

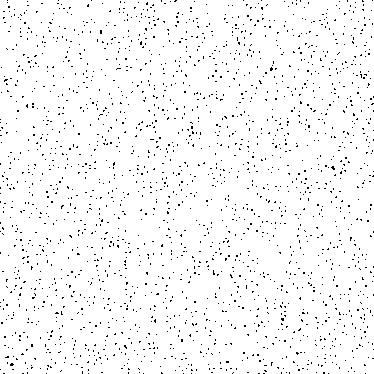

Supplement: Figure 4—source data 1. [file elife-56679-fig4-data1.zip › Figure4 - Source Data1/GFP-PSEN1 BACE1/roi masks/41-ps-2.tif - watershed (h=1404,00, T=4213,00, %=20, n=2130).tif]

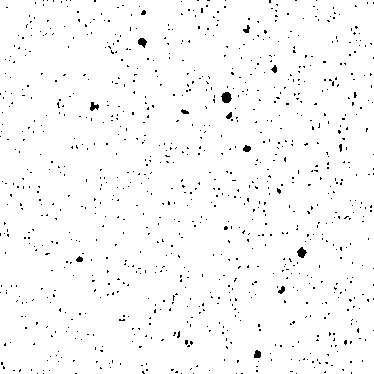

Supplement: Figure 4—source data 1. [file elife-56679-fig4-data1.zip › Figure4 - Source Data1/GFP-PSEN1 BACE1/roi masks/42-bace-2.tif - watershed (h=1404,00, T=4213,00, %=20, n=945).tif]

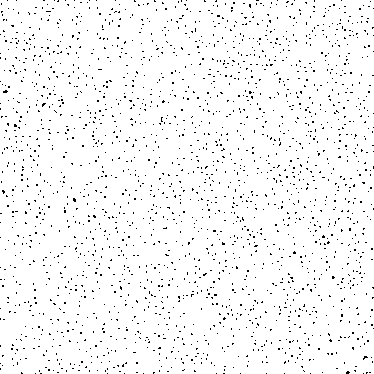

Supplement: Figure 4—source data 1. [file elife-56679-fig4-data1.zip › Figure4 - Source Data1/GFP-PSEN1 BACE1/roi masks/47-ps-1.tif - watershed (h=1404,00, T=4213,00, %=20, n=2194).tif]

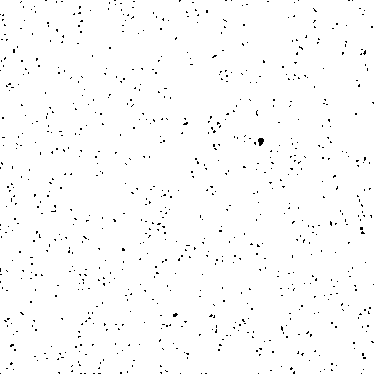

Supplement: Figure 4—source data 1. [file elife-56679-fig4-data1.zip › Figure4 - Source Data1/GFP-PSEN1 BACE1/roi masks/48-bace-1.tif - watershed (h=1404,00, T=4213,00, %=20, n=781).tif]

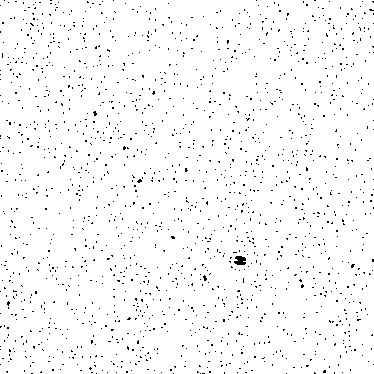

Supplement: Figure 4—source data 1. [file elife-56679-fig4-data1.zip › Figure4 - Source Data1/GFP-PSEN1 BACE1/roi masks/50-ps-1.tif - watershed (h=1404,00, T=4213,00, %=20, n=1711).tif]

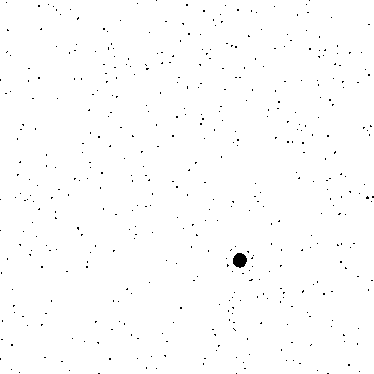

Supplement: Figure 4—source data 1. [file elife-56679-fig4-data1.zip › Figure4 - Source Data1/GFP-PSEN1 BACE1/roi masks/51-bace-1.tif - watershed (h=1404,00, T=4213,00, %=20, n=440).tif]

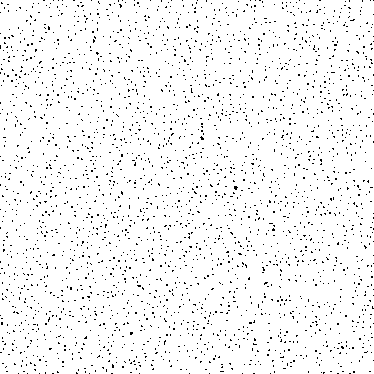

Supplement: Figure 4—source data 1. [file elife-56679-fig4-data1.zip › Figure4 - Source Data1/GFP-PSEN1 BACE1/roi masks/53-ps-1.tif - watershed (h=1404,00, T=4213,00, %=20, n=2690).tif]

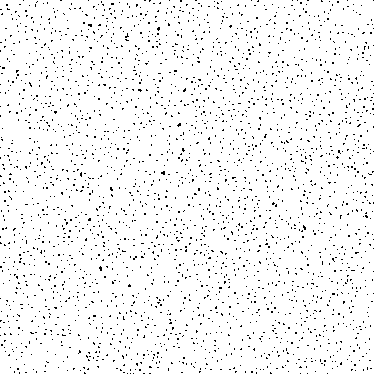

Supplement: Figure 4—source data 1. [file elife-56679-fig4-data1.zip › Figure4 - Source Data1/GFP-PSEN1 BACE1/roi masks/53-ps-2.tif - watershed (h=1404,00, T=4213,00, %=20, n=2676).tif]

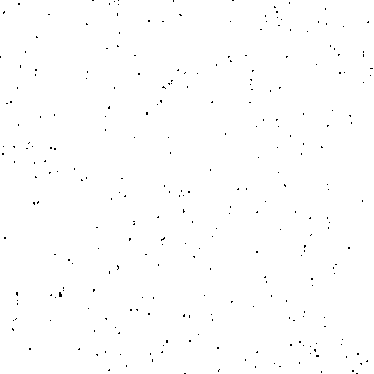

Supplement: Figure 4—source data 1. [file elife-56679-fig4-data1.zip › Figure4 - Source Data1/GFP-PSEN1 BACE1/roi masks/54-bace-1.tif - watershed (h=1404,00, T=4213,00, %=20, n=271).tif]

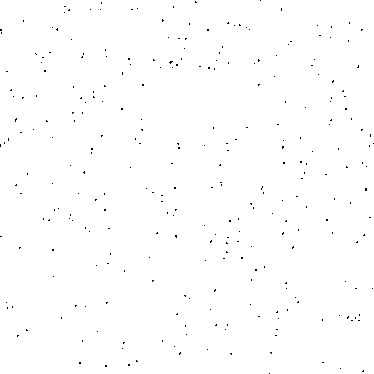

Supplement: Figure 4—source data 1. [file elife-56679-fig4-data1.zip › Figure4 - Source Data1/GFP-PSEN1 BACE1/roi masks/54-bace-2.tif - watershed (h=1404,00, T=4213,00, %=20, n=284).tif]

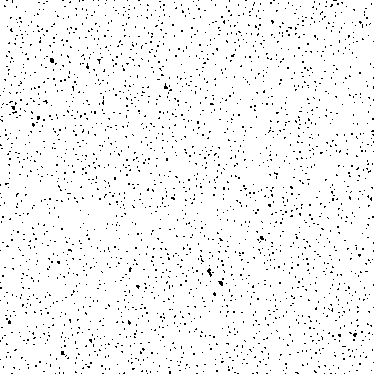

Supplement: Figure 4—source data 1. [file elife-56679-fig4-data1.zip › Figure4 - Source Data1/GFP-PSEN1 BACE1/roi masks/56-ps-1.tif - watershed (h=1404,00, T=4213,00, %=20, n=2399).tif]

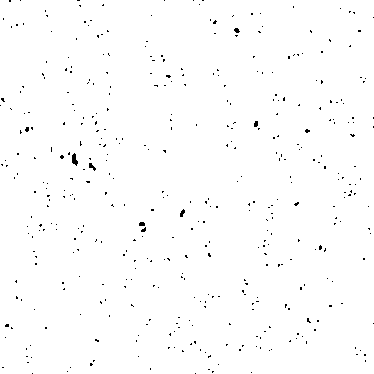

Supplement: Figure 4—source data 1. [file elife-56679-fig4-data1.zip › Figure4 - Source Data1/GFP-PSEN1 BACE1/roi masks/57-bace-1.tif - watershed (h=1404,00, T=4213,00, %=20, n=417).tif]

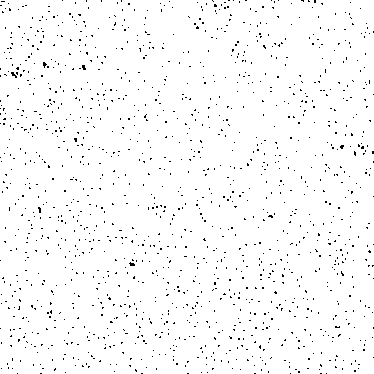

Supplement: Figure 4—source data 1. [file elife-56679-fig4-data1.zip › Figure4 - Source Data1/GFP-PSEN1 BACE1/roi masks/59-ps-1.tif - watershed (h=1404,00, T=4213,00, %=20, n=1440).tif]

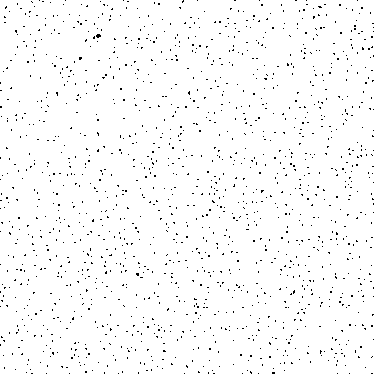

Supplement: Figure 4—source data 1. [file elife-56679-fig4-data1.zip › Figure4 - Source Data1/GFP-PSEN1 BACE1/roi masks/59-ps-2.tif - watershed (h=1404,00, T=4213,00, %=20, n=1484).tif]

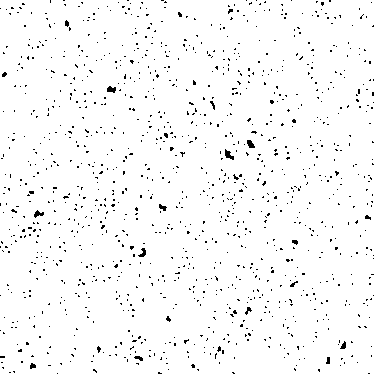

Supplement: Figure 4—source data 1. [file elife-56679-fig4-data1.zip › Figure4 - Source Data1/GFP-PSEN1 BACE1/roi masks/60-bace-1.tif - watershed (h=1404,00, T=4213,00, %=20, n=1178).tif]

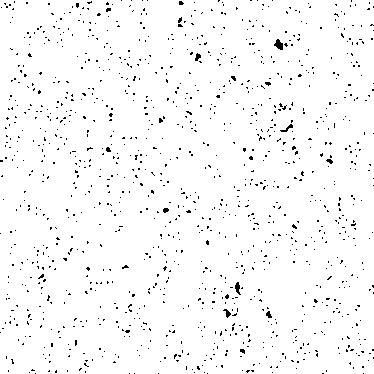

Supplement: Figure 4—source data 1. [file elife-56679-fig4-data1.zip › Figure4 - Source Data1/GFP-PSEN1 BACE1/roi masks/60-bace-2.tif - watershed (h=1404,00, T=4213,00, %=20, n=1189).tif]

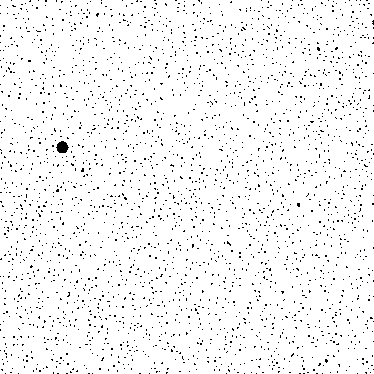

Supplement: Figure 4—source data 1. [file elife-56679-fig4-data1.zip › Figure4 - Source Data1/GFP-PSEN1 BACE1/roi masks/65-ps-1.tif - watershed (h=1404,00, T=4213,00, %=20, n=2626).tif]

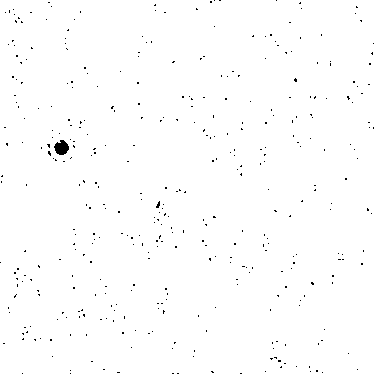

Supplement: Figure 4—source data 1. [file elife-56679-fig4-data1.zip › Figure4 - Source Data1/GFP-PSEN1 BACE1/roi masks/66-bace-1.tif - watershed (h=1404,00, T=4213,00, %=20, n=384).tif]

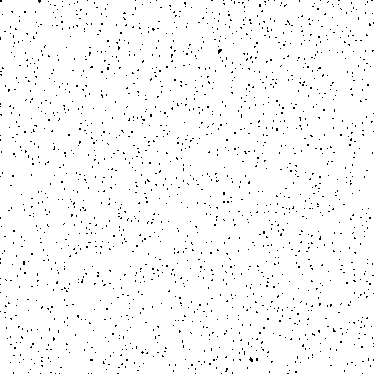

Supplement: Figure 4—source data 1. [file elife-56679-fig4-data1.zip › Figure4 - Source Data1/GFP-PSEN1 BACE1/roi masks/68-ps-1.tif - watershed (h=1404,00, T=4213,00, %=20, n=1740).tif]

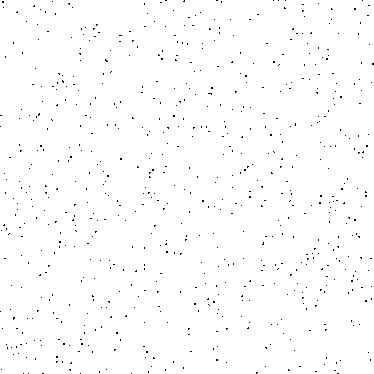

Supplement: Figure 4—source data 1. [file elife-56679-fig4-data1.zip › Figure4 - Source Data1/GFP-PSEN1 BACE1/roi masks/69-bace-1.tif - watershed (h=1404,00, T=4213,00, %=20, n=683).tif]

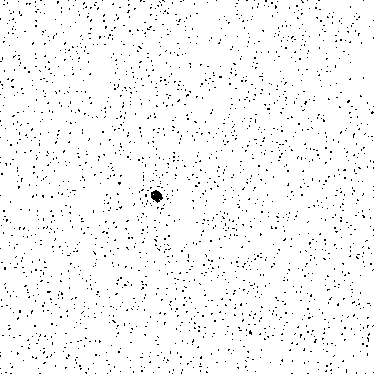

Supplement: Figure 4—source data 1. [file elife-56679-fig4-data1.zip › Figure4 - Source Data1/GFP-PSEN1 BACE1/roi masks/71-ps-1.tif - watershed (h=1404,00, T=4213,00, %=20, n=2116).tif]

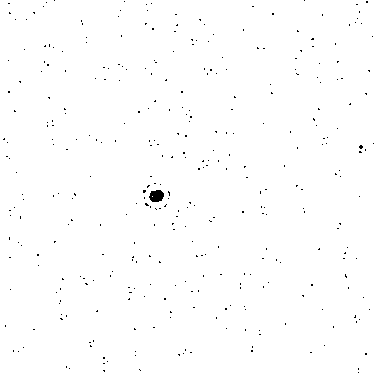

Supplement: Figure 4—source data 1. [file elife-56679-fig4-data1.zip › Figure4 - Source Data1/GFP-PSEN1 BACE1/roi masks/72-bace-1.tif - watershed (h=1404,00, T=4213,00, %=20, n=346).tif]

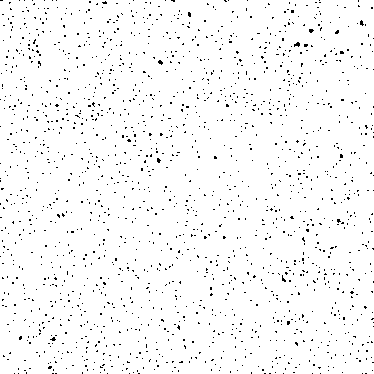

Supplement: Figure 4—source data 1. [file elife-56679-fig4-data1.zip › Figure4 - Source Data1/GFP-PSEN1 BACE1/roi masks/75-ps-1.tif - watershed (h=1404,00, T=4213,00, %=20, n=1718).tif]

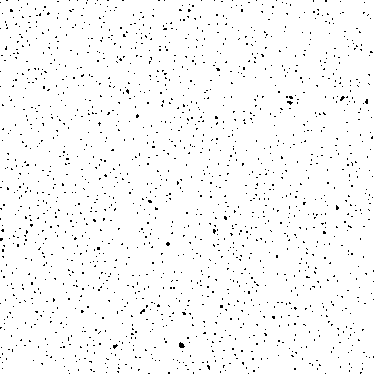

Supplement: Figure 4—source data 1. [file elife-56679-fig4-data1.zip › Figure4 - Source Data1/GFP-PSEN1 BACE1/roi masks/75-ps-2.tif - watershed (h=1404,00, T=4213,00, %=20, n=1658).tif]

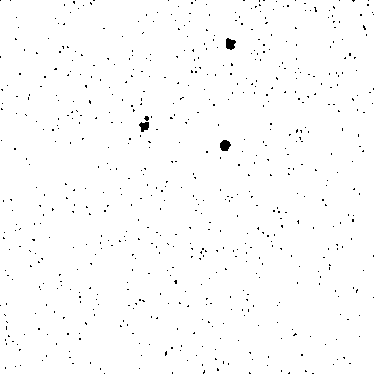

Supplement: Figure 4—source data 1. [file elife-56679-fig4-data1.zip › Figure4 - Source Data1/GFP-PSEN1 BACE1/roi masks/76-bace-1.tif - watershed (h=1404,00, T=4213,00, %=20, n=629).tif]

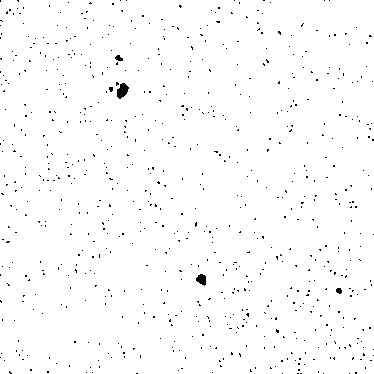

Supplement: Figure 4—source data 1. [file elife-56679-fig4-data1.zip › Figure4 - Source Data1/GFP-PSEN1 BACE1/roi masks/76-bace-2.tif - watershed (h=1404,00, T=4213,00, %=20, n=728).tif]

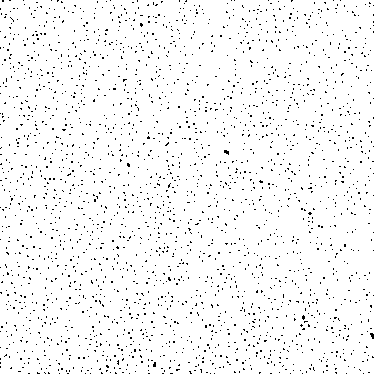

Supplement: Figure 4—source data 1. [file elife-56679-fig4-data1.zip › Figure4 - Source Data1/GFP-PSEN1 BACE1/roi masks/78-ps-1.tif - watershed (h=1404,00, T=4213,00, %=20, n=2370).tif]

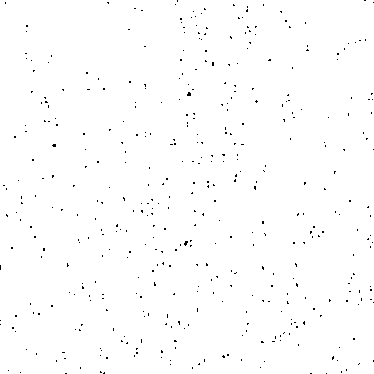

Supplement: Figure 4—source data 1. [file elife-56679-fig4-data1.zip › Figure4 - Source Data1/GFP-PSEN1 BACE1/roi masks/79-bace-1.tif - watershed (h=1404,00, T=4213,00, %=20, n=428).tif]

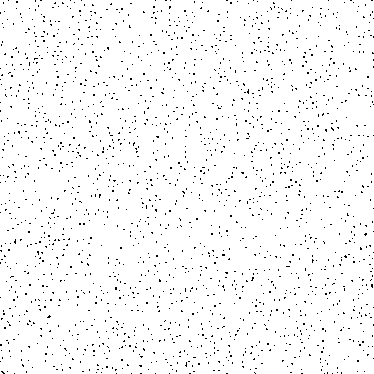

Supplement: Figure 4—source data 1. [file elife-56679-fig4-data1.zip › Figure4 - Source Data1/GFP-PSEN1 BACE1/roi masks/84-ps-1.tif - watershed (h=1404,00, T=4213,00, %=20, n=1979).tif]

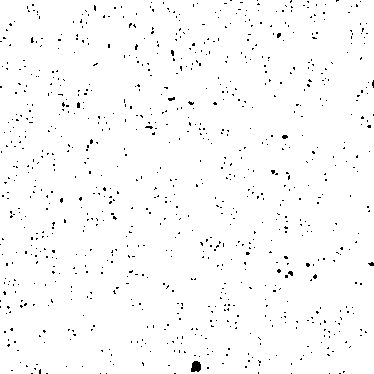

Supplement: Figure 4—source data 1. [file elife-56679-fig4-data1.zip › Figure4 - Source Data1/GFP-PSEN1 BACE1/roi masks/85-bace-1.tif - watershed (h=1404,00, T=4213,00, %=20, n=868).tif]

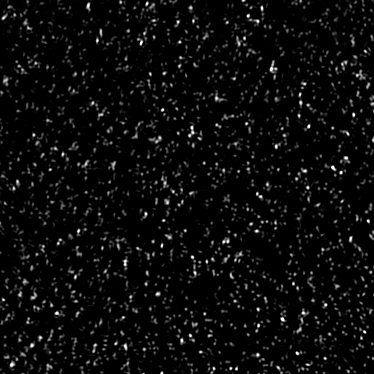

Supplement: Figure 4—source data 1. [file elife-56679-fig4-data1.zip › Figure4 - Source Data1/GFP-PSEN1 BACE1/rois/41-ps-2.tif]

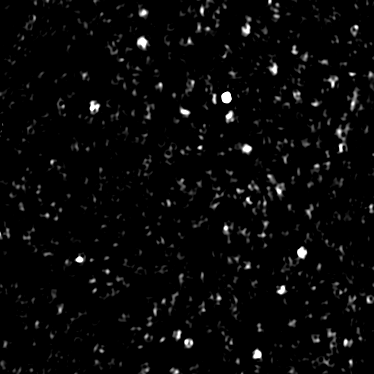

Supplement: Figure 4—source data 1. [file elife-56679-fig4-data1.zip › Figure4 - Source Data1/GFP-PSEN1 BACE1/rois/42-bace-2.tif]

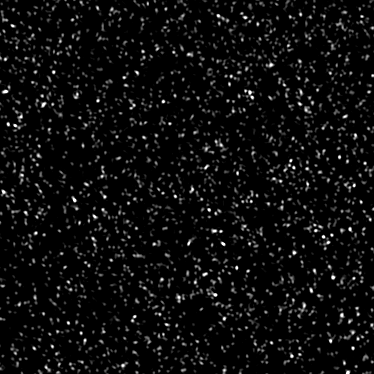

Supplement: Figure 4—source data 1. [file elife-56679-fig4-data1.zip › Figure4 - Source Data1/GFP-PSEN1 BACE1/rois/47-ps-1.tif]

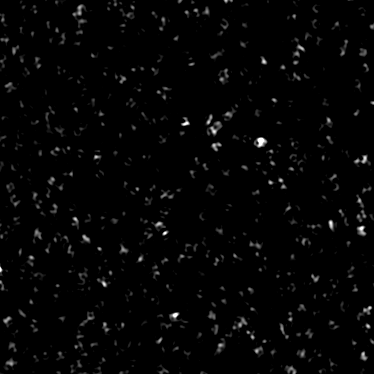

Supplement: Figure 4—source data 1. [file elife-56679-fig4-data1.zip › Figure4 - Source Data1/GFP-PSEN1 BACE1/rois/48-bace-1.tif]

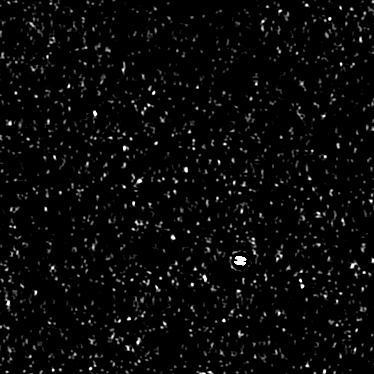

Supplement: Figure 4—source data 1. [file elife-56679-fig4-data1.zip › Figure4 - Source Data1/GFP-PSEN1 BACE1/rois/50-ps-1.tif]

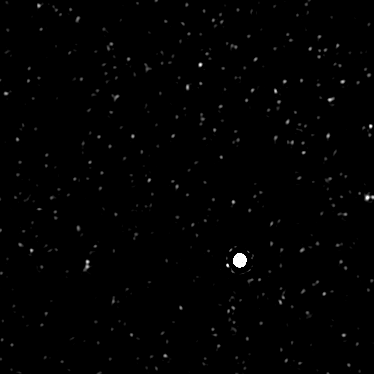

Supplement: Figure 4—source data 1. [file elife-56679-fig4-data1.zip › Figure4 - Source Data1/GFP-PSEN1 BACE1/rois/51-bace-1.tif]

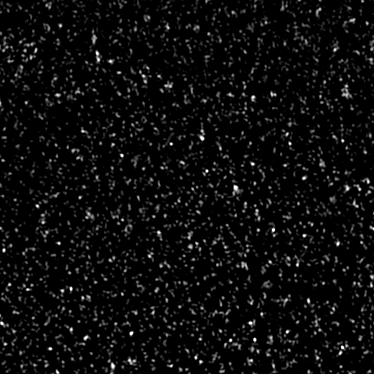

Supplement: Figure 4—source data 1. [file elife-56679-fig4-data1.zip › Figure4 - Source Data1/GFP-PSEN1 BACE1/rois/53-ps-1.tif]

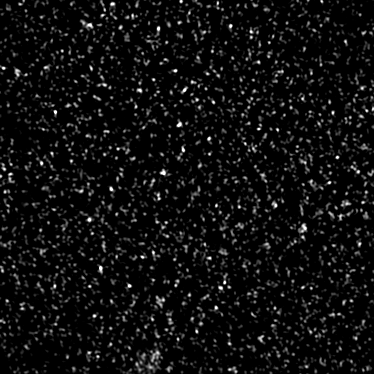

Supplement: Figure 4—source data 1. [file elife-56679-fig4-data1.zip › Figure4 - Source Data1/GFP-PSEN1 BACE1/rois/53-ps-2.tif]

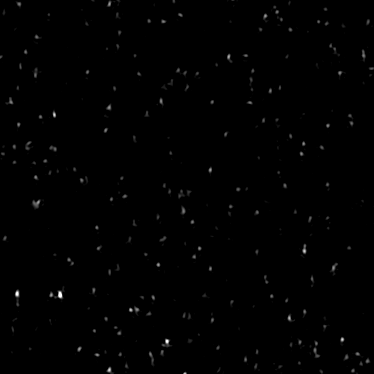

Supplement: Figure 4—source data 1. [file elife-56679-fig4-data1.zip › Figure4 - Source Data1/GFP-PSEN1 BACE1/rois/54-bace-1.tif]

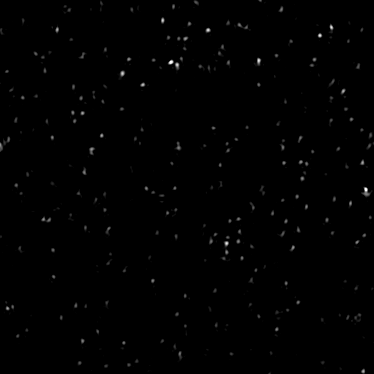

Supplement: Figure 4—source data 1. [file elife-56679-fig4-data1.zip › Figure4 - Source Data1/GFP-PSEN1 BACE1/rois/54-bace-2.tif]

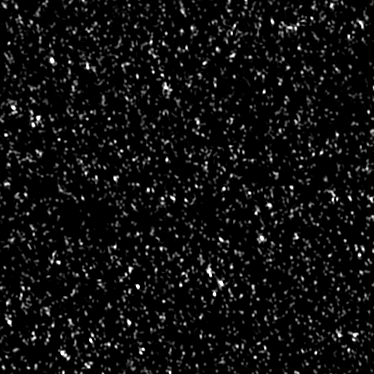

Supplement: Figure 4—source data 1. [file elife-56679-fig4-data1.zip › Figure4 - Source Data1/GFP-PSEN1 BACE1/rois/56-ps-1.tif]

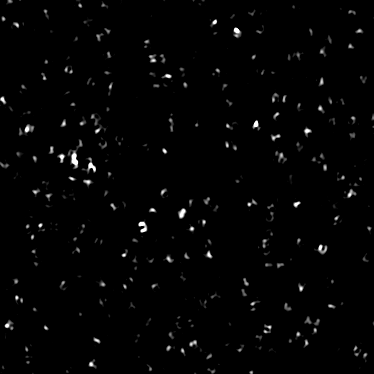

Supplement: Figure 4—source data 1. [file elife-56679-fig4-data1.zip › Figure4 - Source Data1/GFP-PSEN1 BACE1/rois/57-bace-1.tif]

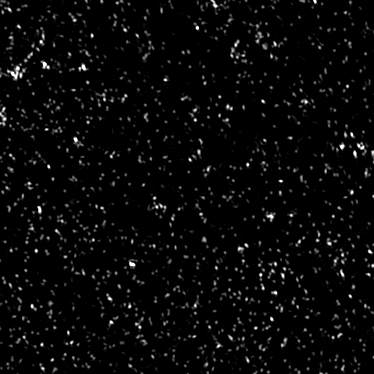

Supplement: Figure 4—source data 1. [file elife-56679-fig4-data1.zip › Figure4 - Source Data1/GFP-PSEN1 BACE1/rois/59-ps-1.tif]

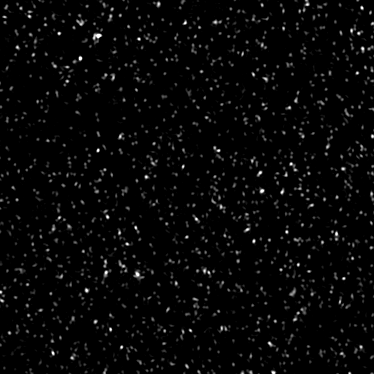

Supplement: Figure 4—source data 1. [file elife-56679-fig4-data1.zip › Figure4 - Source Data1/GFP-PSEN1 BACE1/rois/59-ps-2.tif]

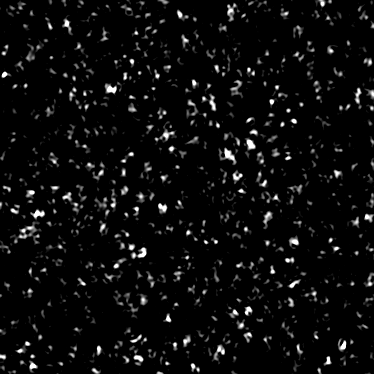

Supplement: Figure 4—source data 1. [file elife-56679-fig4-data1.zip › Figure4 - Source Data1/GFP-PSEN1 BACE1/rois/60-bace-1.tif]

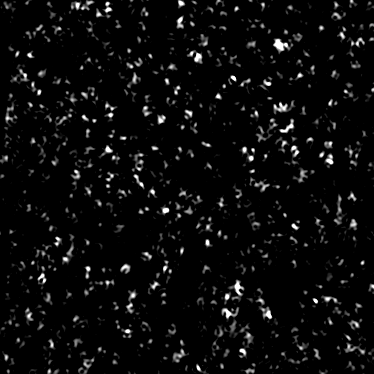

Supplement: Figure 4—source data 1. [file elife-56679-fig4-data1.zip › Figure4 - Source Data1/GFP-PSEN1 BACE1/rois/60-bace-2.tif]

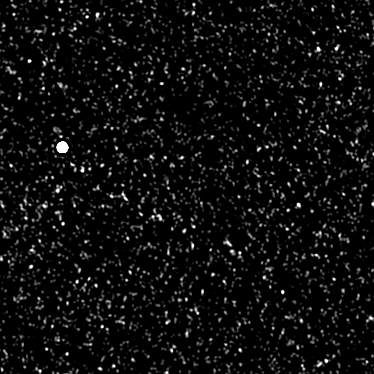

Supplement: Figure 4—source data 1. [file elife-56679-fig4-data1.zip › Figure4 - Source Data1/GFP-PSEN1 BACE1/rois/65-ps-1.tif]
